# Supplementary material for: Development of the simulation-based German albuminuria screening model (S-GASM) for estimating the cost-effectiveness of albuminuria screening in Germany
Source: PLoS One. 2022 Jan 5;17(1):e0262227. doi: 10.1371/journal.pone.0262227 (PMC8730388; doi:10.1371/journal.pone.0262227)
Supplement: S1 File — (DOCX) [file pone.0262227.s001.docx]

**Supplementary Material – Model Development and parameter values**

[Parameters 1](#_Toc78191973)

[**Age and Gender** 1](#_Toc78191974)

[**Body-Mass-Index (BMI)** 2](#_Toc78191975)

[**Systolic blood pressure (SBP)** 4](#_Toc78191976)

[**Therapy of hypertension** 6](#_Toc78191977)

[**Diabetes** 6](#_Toc78191978)

[**Albuminuria and glomerular filtration rate (GFR)** 10](#_Toc78191979)

[**Quality of Life** 14](#_Toc78191980)

[**Testing** 14](#_Toc78191981)

[**Therapy caused by testing and adherence** 14](#_Toc78191982)

[**Costs** 14](#_Toc78191983)

[**Death** 15](#_Toc78191984)

[Sensitivity Analysis 17](#_Toc78191985)

[Validation Analysis 18](#_Toc78191986)

[Citations 20](#_Toc78191987)

# **Parameters**

## **Age and Gender**

For generating the sex and age of individuals in the cohort, we used the age and sex distribution of the German population for 2016 [1] (**Table S1)**. The simulation starts in the year 2016 because that is the year the distribution is referring to. Since the simulation should include only persons aged 30-90 years, we consider only birth years 1927-1998. The cumulative chance is only 82,8% because, in contrast to the data source, the S-GASM only regards individuals from 18 to 90.

We generate a value from the uniform distribution between 0 and the maximum of the cumulative chance of all included ages and then assign the birth year based on the next higher category.

For assigning sex, the model generates a uniformly distributed value between 0 and 1. The S-GASM compares it to the proportion of women of the assigned age (when the simulated value is below the estimated proportion, S-GASM sets female sex, otherwise male sex).

**Fig S1** shows the distribution of age and gender at the start of a simulation with 1 million individuals compared to published data.

| **Table S1** – Age and sex distribution in the German population for 2016 [1] | | | |
| --- | --- | --- | --- |
| Year of birth | Chance | Cumulative chance | Women per 1000 men |
| 1998 | *1,03324275387455* | 1,03324275387455 | 900,265210608424 |
| 1997 | *1,08883664751602* | 2,12207940139057 | 893,686839415366 |
| 1996 | *1,08682868967736* | 3,20890809106793 | 900,085802357976 |
| 1995 | *1,07107767218381* | 4,27998576325174 | 902,942240287980 |
| 1994 | *1,09101910500993* | 5,37100486826167 | 906,595908237103 |
| 1993 | *1,13407447133906* | 6,50507933960072 | 914,225111271109 |
| 1992 | *1,15982528852155* | 7,66490462812227 | 920,644317173898 |
| 1991 | *1,20711469509706* | 8,87201932321933 | 923,645562775788 |
| 1990 | *1,32572841215384* | 10,19774773537320 | 920,608197072424 |
| 1989 | *1,31057239001259* | 11,50832012538580 | 929,327941116132 |
| 1988 | *1,34375761959107* | 12,85207774497680 | 924,616687667705 |
| 1987 | *1,31627877110023* | 14,16835651607710 | 929,577264628399 |
| 1986 | *1,29445177255477* | 15,46280828863180 | 941,273109572038 |
| 1985 | *1,25582554678104* | 16,71863383541290 | 944,377630223627 |
| 1984 | *1,24802032261763* | 17,96665415803050 | 950,225910173722 |
| 1983 | *1,25080019907018* | 19,21745435710070 | 957,969835100319 |
| 1982 | *1,27781735055647* | 20,49527170765710 | 961,162701165565 |
| 1981 | *1,26689536866160* | 21,76216707631870 | 974,110014029801 |
| 1980 | *1,27293863102815* | 23,03510570734690 | 976,606998707288 |
| 1979 | *1,21139599566674* | 24,24650170301360 | 979,858075683726 |
| 1978 | *1,19434107797138* | 25,44084278098500 | 979,633033051128 |
| 1977 | *1,18377536620601* | 26,62461814719100 | 978,288605010976 |
| 1976 | *1,16075837695592* | 27,78537652414690 | 984,871184382570 |
| 1975 | *1,13076140149544* | 28,91613792564240 | 980,127067930838 |
| 1974 | *1,14537695942664* | 30,06151488506900 | 988,262071344729 |
| 1973 | *1,15487749621302* | 31,21639238128200 | 990,484345955429 |
| 1972 | *1,25152364555761* | 32,46791602683970 | 984,768003197835 |
| 1971 | *1,37637814889627* | 33,84429417573590 | 981,797910039939 |
| 1970 | *1,42719147906550* | 35,27148565480140 | 982,570432018961 |
| 1969 | *1,53212030301914* | 36,80360595782060 | 977,241094579336 |
| 1968 | *1,60414988294042* | 38,40775584076100 | 976,798466073525 |
| 1967 | *1,64594133857207* | 40,05369717933300 | 978,589262875140 |
| 1966 | *1,68920149963550* | 41,74289867896850 | 977,687197981959 |
| 1965 | *1,69380150443666* | 43,43670018340520 | 974,874535158413 |
| 1964 | *1,72312471733934* | 45,15982490074450 | 978,845661419251 |
| 1963 | *1,70710589134709* | 46,86693079209160 | 982,571443851170 |
| 1962 | *1,65037169093062* | 48,51730248302220 | 989,624664722691 |
| 1961 | *1,62431065213878* | 50,14161313516100 | 997,722693183580 |
| 1960 | *1,56700690423639* | 51,70862003939740 | 1001,365073616470 |
| 1959 | *1,52027371531203* | 53,22889375470940 | 1003,578974602220 |
| 1958 | *1,43305902997362* | 54,66195278468310 | 1007,131812943510 |
| 1957 | *1,39655345973256* | 56,05850624441560 | 1013,690085128390 |
| 1956 | *1,35437543889238* | 57,41288168330800 | 1022,996557304050 |
| 1955 | *1,30847112333050* | 58,72135280663850 | 1037,263377552540 |
| 1954 | *1,27907883764762* | 60,00043164428610 | 1057,389622970660 |
| 1953 | *1,23258195033975* | 61,23301359462590 | 1071,139573003740 |
| 1952 | *1,22536445070968* | 62,45837804533560 | 1077,092619600270 |
| 1951 | *1,19497969823750* | 63,65335774357310 | 1081,034653768320 |
| 1950 | *1,18833901691232* | 64,84169676048540 | 1086,150998787410 |
| 1949 | *1,14218143448968* | 65,98387819497510 | 1085,142579032370 |
| 1948 | *1,03677152468092* | 67,02064971965600 | 1084,151831389400 |
| 1947 | *0,96755090448806* | 67,98820062414410 | 1100,375913148020 |
| 1946 | *0,83634170537035* | 68,82454232951440 | 1113,006925351930 |
| 1945 | *0,72895413280197* | 69,55349646231640 | 1145,340875048150 |
| 1944 | *0,96531270404872* | 70,51880916636510 | 1145,292078821290 |
| 1943 | *0,96993694491311* | 71,48874611127820 | 1153,203005415240 |
| 1942 | *0,92851872465521* | 72,41726483593340 | 1166,679862685990 |
| 1941 | *1,11181728267125* | 73,52908211860470 | 1198,929163750710 |
| 1940 | *1,14816410669815* | 74,67724622530280 | 1228,802495360010 |
| 1939 | *1,10609999535516* | 75,78334622065800 | 1256,727990169780 |
| 1938 | *1,00115784156675* | 76,78450406222470 | 1280,888422621920 |
| 1937 | *0,90356769755933* | 77,68807175978410 | 1318,204853176640 |
| 1936 | *0,83916157132723* | 78,52723333111130 | 1359,991684530160 |
| 1935 | *0,77137814968394* | 79,29861148079520 | 1409,108799975780 |
| 1934 | *0,67540091568452* | 79,97401239647970 | 1459,542469815720 |
| 1933 | *0,50876707474583* | 80,48277947122560 | 1527,058667742070 |
| 1932 | *0,47106666658750* | 80,95384613781300 | 1606,542977262520 |
| 1931 | *0,44104909047326* | 81,39489522828630 | 1694,510457153430 |
| 1930 | *0,42220676311465* | 81,81710199140090 | 1800,762064003730 |
| 1929 | *0,36788041558014* | 82,18498240698110 | 1904,136453211400 |
| 1928 | *0,32101877552065* | 82,50600118250170 | 1995,499570310730 |
| 1927 | *0,26232872480148* | 82,76832990730320 | 2218,620833209430 |

**Fig S1 –** Distribution of Age (left) and Gender (right) at start of a simulation with 1 million individuals


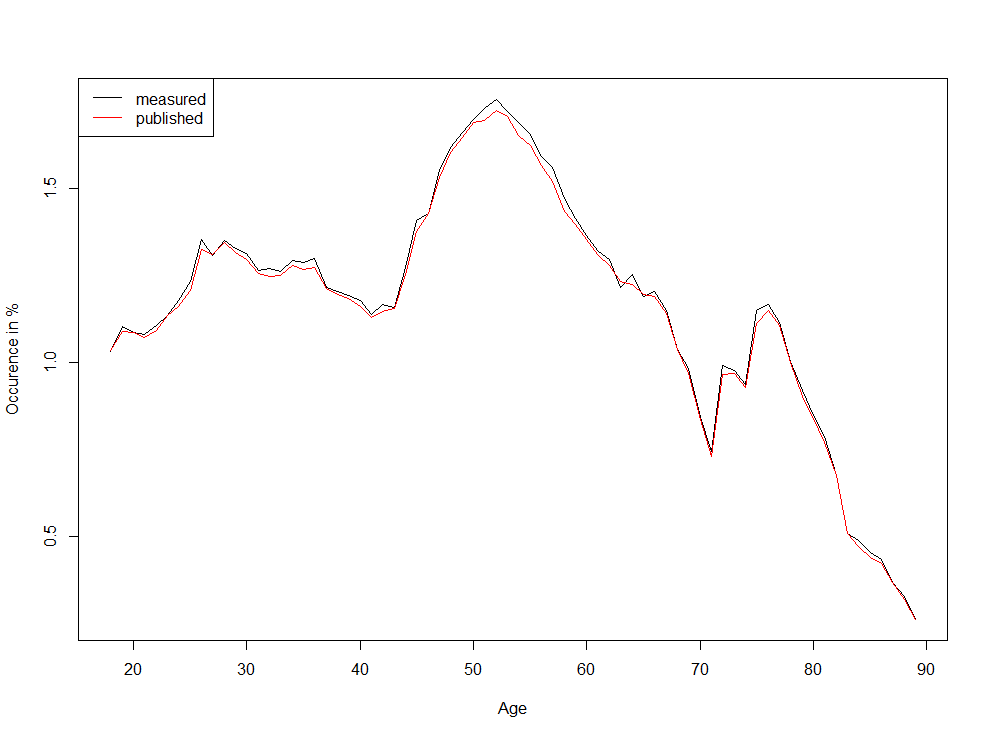

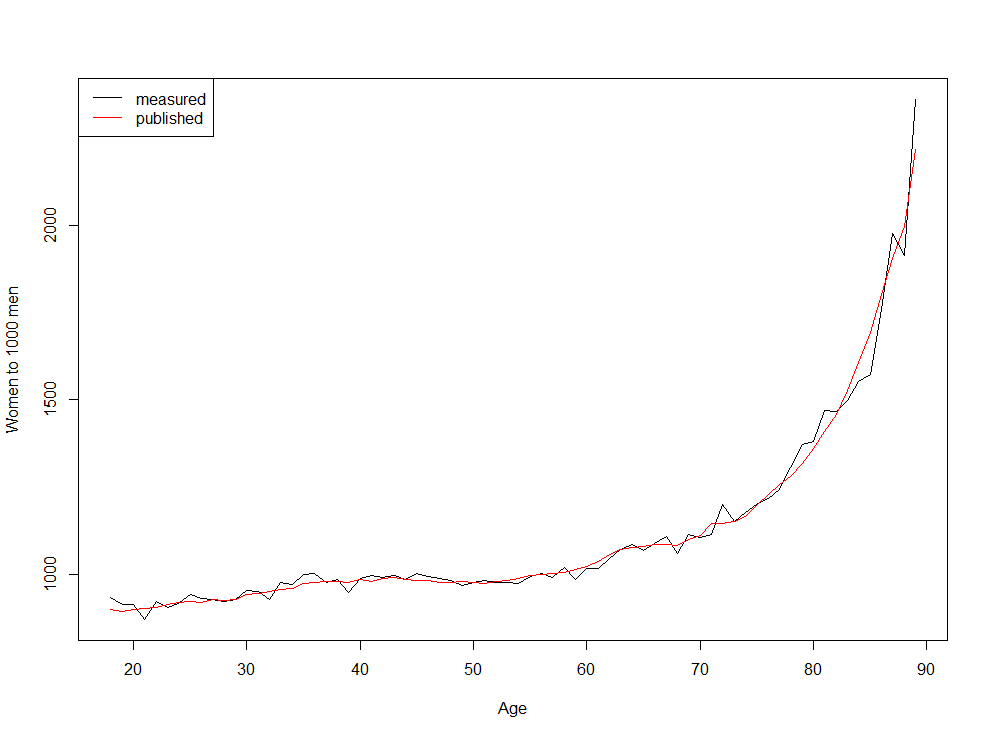


## **Body-Mass-Index (BMI)**

The model utilizes German BMI percentiles [2] to generate a BMI of an individuum, depending on age and sex. These percentiles are given in the Lambda-Mu-Sigma-(LMS)-format [3]. Lambda, Mu, and Sigma define the distribution of percentiles, while a standard deviation score (SDS value) defines an individual's percentile. These SDS values are, by definition, normally distributed regarding the population they describe. That enables the simulation of a normally distributed SDS value with a mean of 0 and a standard deviation of 1 for an individual. With Lambda, Mu, and Sigma corresponding to age and gender, the S-GASM assigns the BMI. As individuals progress in the simulation, the SDS value does not change, while Lambda, Mu, and Sigma change correspondingly, and so individuals remain at the initially assigned percentiles while their BMI changes. This is allowing a reasoned, age-dependent change.

The cited source only gives percentiles for the ages up to 79. So, we had to extrapolate the available BMI percentiles using the 3rd-grade polynomial for the ages of 80 to 90. **Fig S2** shows the initial percentiles and extrapolation. **Table S2** shows the values used in the S-GASM. An additional benefit of using the LMS format is that it allows measuring the corresponding parameter distribution by measuring the mean and standard deviation of SDS values. **Fig S3** shows the mean and standard deviation of SDS values of BMI at the start of a simulation with 1 million individuals.


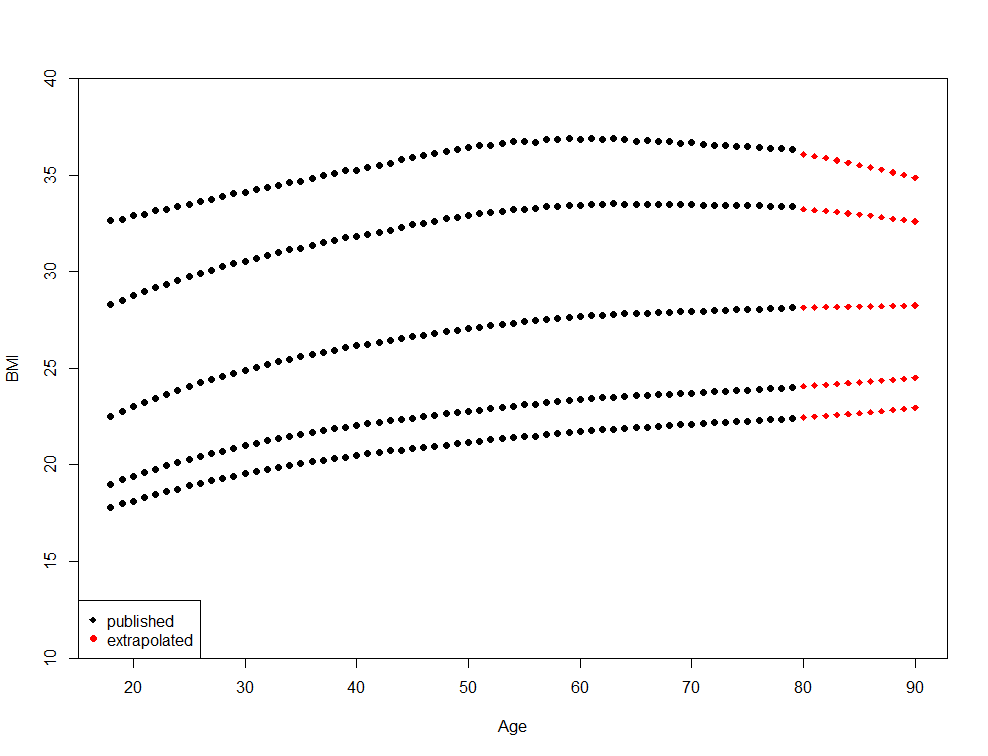

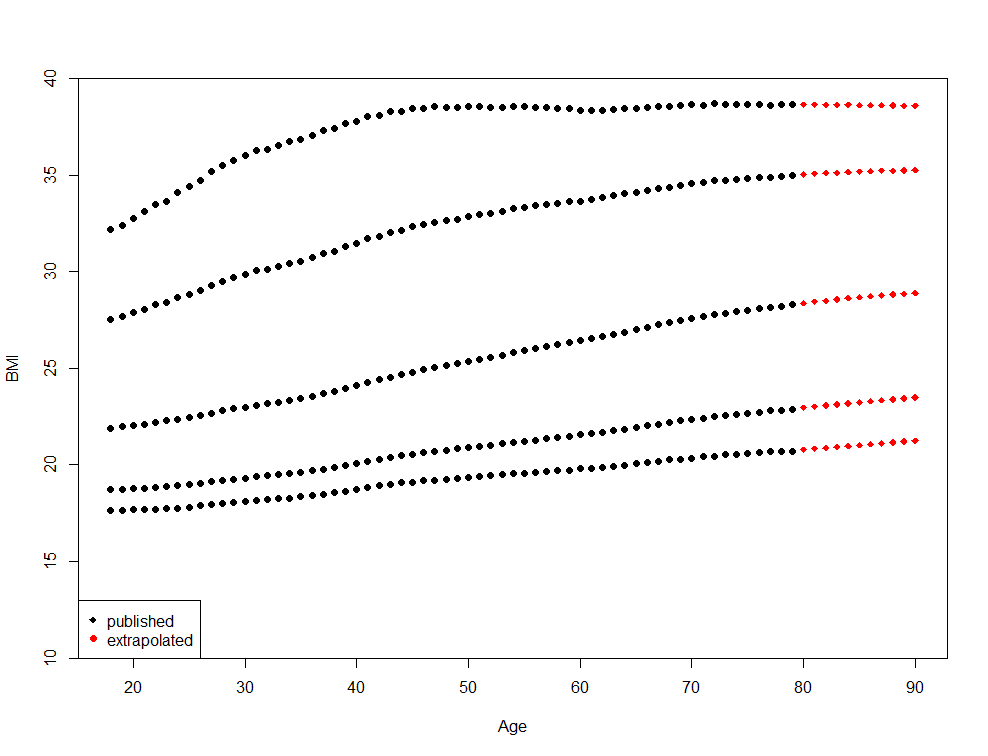


**Figure S2** – 3^rd^, 10^th^, 50^th^, 90^th^,97^th^ BMI percentiles for females (left) and males (right) with published and extrapolated data

| **Table S2** – German BMI percentiles [2] expanded for Ages 80-90 | | | | | | |
| --- | --- | --- | --- | --- | --- | --- |
| Age | Women | | | Men | | |
|  | Lambda | Sigma | Mu | Lambda | Sigma | Mu |
| 18 | -1.95 | 0.144 | 21.9 | -1.51 | 0.151 | 22.53 |
| 19 | -1.941 | 0.145 | 21.98 | -1.444 | 0.15 | 22.78 |
| 20 | -1.933 | 0.147 | 22.05 | -1.381 | 0.15 | 23.01 |
| 21 | -1.927 | 0.149 | 22.12 | -1.319 | 0.149 | 23.24 |
| 22 | -1.924 | 0.151 | 22.2 | -1.262 | 0.149 | 23.46 |
| 23 | -1.896 | 0.152 | 22.28 | -1.208 | 0.148 | 23.67 |
| 24 | -1.919 | 0.154 | 22.36 | -1.158 | 0.148 | 23.87 |
| 25 | -1.917 | 0.155 | 22.46 | -1.111 | 0.147 | 24.07 |
| 26 | -1.914 | 0.156 | 22.57 | -1.068 | 0.147 | 24.25 |
| 27 | -1.91 | 0.158 | 22.69 | -1.028 | 0.146 | 24.43 |
| 28 | -1.904 | 0.159 | 22.8 | -0.992 | 0.146 | 24.6 |
| 29 | -1.896 | 0.16 | 22.91 | -0.959 | 0.146 | 24.76 |
| 30 | -1.886 | 0.161 | 23 | -0.929 | 0.145 | 24.91 |
| 31 | -1.874 | 0.162 | 23.1 | -0.902 | 0.145 | 25.06 |
| 32 | -1.86 | 0.162 | 23.18 | -0.878 | 0.144 | 25.21 |
| 33 | -1.845 | 0.163 | 23.26 | -0.857 | 0.144 | 25.34 |
| 34 | -1.829 | 0.164 | 23.35 | -0.838 | 0.144 | 25.48 |
| 35 | -1.811 | 0.164 | 23.45 | -0.821 | 0.143 | 25.6 |
| 36 | -1.792 | 0.165 | 23.56 | -0.805 | 0.143 | 25.72 |
| 37 | -1.771 | 0.166 | 23.68 | -0.792 | 0.143 | 25.84 |
| 38 | -1.749 | 0.166 | 23.81 | -0.779 | 0.143 | 25.95 |
| 39 | -1.724 | 0.167 | 23.96 | -0.768 | 0.143 | 26.06 |
| 40 | -1.697 | 0.167 | 24.12 | -0.758 | 0.142 | 26.16 |
| 41 | -1.677 | 0.168 | 24.27 | -0.749 | 0.142 | 26.26 |
| 42 | -1.635 | 0.168 | 24.42 | -0.741 | 0.142 | 26.36 |
| 43 | -1.601 | 0.169 | 24.55 | -0.734 | 0.142 | 26.45 |
| 44 | -1.564 | 0.169 | 24.68 | -0.728 | 0.143 | 26.54 |
| 45 | -1.526 | 0.17 | 24.8 | -0.722 | 0.143 | 26.64 |
| 46 | -1.485 | 0.17 | 24.92 | -0.717 | 0.143 | 26.72 |
| 47 | -1.442 | 0.171 | 25.03 | -0.713 | 0.143 | 26.81 |
| 48 | -1.397 | 0.171 | 25.13 | -0.708 | 0.143 | 26.9 |
| 49 | -1.35 | 0.171 | 25.24 | -0.704 | 0.143 | 26.98 |
| 50 | -1.302 | 0.172 | 25.35 | -0.7 | 0.143 | 27.06 |
| 51 | -1.253 | 0.172 | 25.46 | -0.696 | 0.143 | 27.14 |
| 52 | -1.202 | 0.172 | 25.57 | -0.691 | 0.142 | 27.21 |
| 53 | -1.15 | 0.172 | 25.69 | -0.686 | 0.142 | 27.28 |
| 54 | -1.097 | 0.173 | 25.8 | -0.682 | 0.142 | 27.35 |
| 55 | -1.043 | 0.173 | 25.91 | -0.677 | 0.141 | 27.42 |
| 56 | -0.989 | 0.173 | 26.01 | -0.614 | 0.141 | 27.48 |
| 57 | -0.934 | 0.173 | 26.12 | -0.666 | 0.141 | 27.54 |
| 58 | -0.88 | 0.173 | 26.22 | -0.666 | 0.14 | 27.59 |
| 59 | -0.828 | 0.173 | 26.32 | -0.654 | 0.14 | 27.64 |
| 60 | -0.776 | 0.172 | 26.42 | -0.647 | 0.139 | 27.68 |
| 61 | -0.725 | 0.172 | 26.53 | -0.64 | 0.139 | 27.72 |
| 62 | -0.676 | 0.172 | 26.64 | -0.633 | 0.138 | 27.76 |
| 63 | -0.629 | 0.172 | 26.76 | -0.625 | 0.138 | 27.79 |
| 64 | -0.582 | 0.172 | 26.88 | -0.617 | 0.137 | 27.82 |
| 65 | -0.538 | 0.171 | 27 | -0.609 | 0.136 | 27.84 |
| 66 | -0.495 | 0.171 | 27.13 | -0.6 | 0.136 | 27.86 |
| 67 | -0.453 | 0.171 | 27.25 | -0.592 | 0.135 | 27.89 |
| 68 | -0.413 | 0.17 | 27.36 | -0.583 | 0.135 | 27.91 |
| 69 | -0.373 | 0.17 | 27.47 | -0.574 | 0.134 | 27.93 |
| 70 | -0.335 | 0.17 | 27.58 | -0.565 | 0.134 | 27.95 |
| 71 | -0.291 | 0.169 | 27.67 | -0.556 | 0.133 | 27.97 |
| 72 | -0.26 | 0.169 | 27.77 | -0.547 | 0.132 | 27.99 |
| 73 | -0.224 | 0.168 | 27.85 | -0.538 | 0.132 | 28.01 |
| 74 | -0.188 | 0.168 | 27.93 | -0.529 | 0.131 | 28.03 |
| 75 | -0.153 | 0.167 | 28.01 | -0.521 | 0.131 | 28.05 |
| 76 | -0.118 | 0.167 | 28.08 | -0.512 | 0.13 | 28.07 |
| 77 | -0.087 | 0.166 | 28.15 | -0.503 | 0.129 | 28.09 |
| 78 | -0.05 | 0.166 | 28.22 | -0.495 | 0.129 | 28.11 |
| 79 | -0.016 | 0.166 | 28.29 | -0.486 | 0.128 | 28.13 |
| 80 | 0.023628857478332 | 0.16486526723373 | 28.37703672167 | -0.417463013919637 | 0.125576719339602 | 28.1366095420771 |
| 81 | 0.0510254649162233 | 0.164276208316954 | 28.4447788022701 | -0.391324166680538 | 0.124415814428739 | 28.1494202135855 |
| 82 | 0.0764637482239265 | 0.163669885766826 | 28.5092703858471 | -0.363214605716983 | 0.123198275149182 | 28.1617759469476 |
| 83 | 0.0998565246622788 | 0.163046851702294 | 28.5704145464959 | -0.333055513025465 | 0.12192256893203 | 28.1737690941769 |
| 84 | 0.121116611492118 | 0.162407658242307 | 28.6281143583118 | -0.300768070602476 | 0.120587163208385 | 28.1854920072869 |
| 85 | 0.140156825974281 | 0.161752857505812 | 28.6822728953899 | -0.266273460444508 | 0.119190525409349 | 28.197037038291 |
| 86 | 0.156889985369605 | 0.161083001611759 | 28.7327932318252 | -0.229492864548055 | 0.117731122966021 | 28.2084965392029 |
| 87 | 0.171228906938927 | 0.160398642679096 | 28.7795784417129 | -0.190347464909608 | 0.116207423309503 | 28.2199628620359 |
| 88 | 0.183086407943086 | 0.159700332826772 | 28.822531599148 | -0.14875844352566 | 0.114617893870896 | 28.2315283588037 |
| 89 | 0.192375305642917 | 0.158988624173736 | 28.8615557782258 | -0.104646982392703 | 0.112961002081302 | 28.2432853815197 |
| 90 | **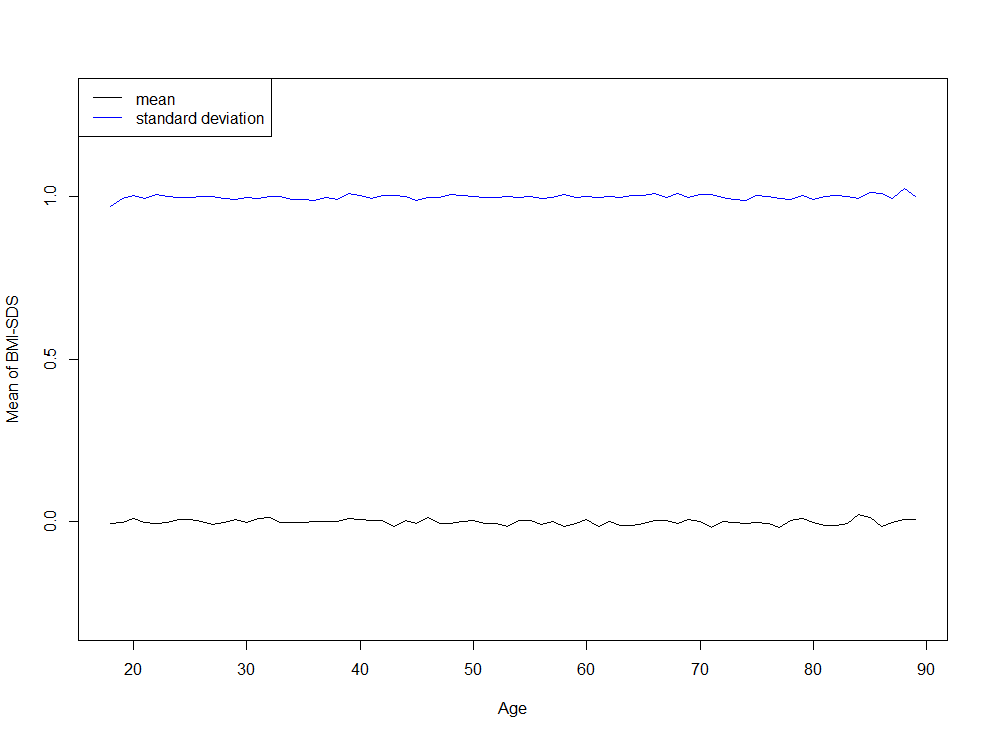**0.19900841729926 | 0.158264068838936 | 28.8965540530414 | -0.0579342635072297 | 0.111235215371821 | 28.2553262821974 |

**Fig S3 –** Mean and standard deviation of BMI-SDS values at start of a simulation with 1 million individuals

## **Systolic blood pressure (SBP)**

For allowing the SBP to be distributed among the population and to change over the simulation, we decided to take the same approach we chose for the BMI. We extrapolated existing German blood pressure age and sex percentiles, given in steps of 5 years,[4] using a 3rd-grade polynomial for steady changes and converted them to the LMS format. **Fig S4** shows original data, extrapolation, and the LMS-formatted percentiles.

For the SBP being BMI-dependent, we decided to use published data stating how the SBP increases depending on a specific increase of BMI.[5] **Table S3** shows the published data. These data only give values for an increase of 5 in the BMI. We assumed the effect to be steady for values between these steps. The S-GASM starts by creating an SDS value and the corresponding SBP and then raises this SBP BMI-dependent. Afterward, the new SDS value is calculated, and the individual keeps this adjusted SDS value.

If the simulation of SDS values were done with a mean of 0 and a standard deviation of 1, the adjustment for the BMI would lead to a right-shift of the normal distribution of SDS values. To compensate for this, we measured this right-shift age- and sex-dependent and decreased the mean of SDS values on creation. As a result, the mean of simulated SDS values is 0, and the distribution of the SBP matches the underlying data. **Table S4** shows the LMS data of SBP percentiles and the mean used to create SDS values. **Fig S5** shows the mean of measured SDS values for SBP at the start of a simulation of 1 million individuals, where statistical fluctuations are visible, but the mean of SDS-values is close to 0 while BMI influences SBP.

**Figure S4** – 5^th^, 25^th^, 50^th^, 75^th^, 95^th^ SBP percentiles for females (left) and males (right) with published, extrapolated and LMS-formatted data


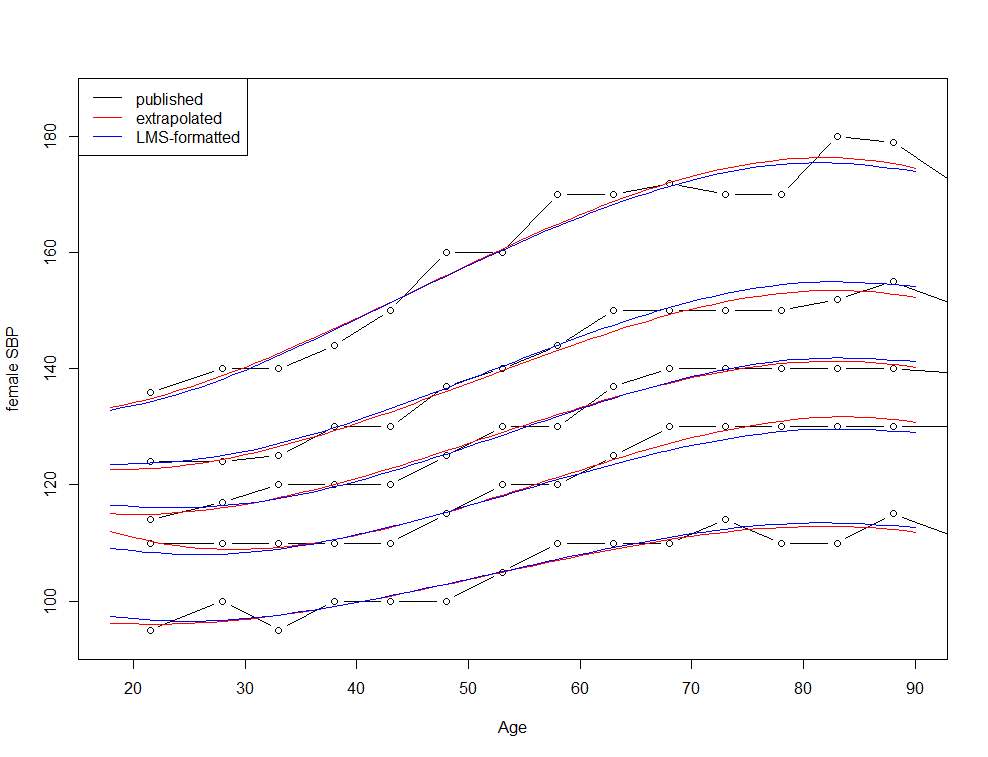

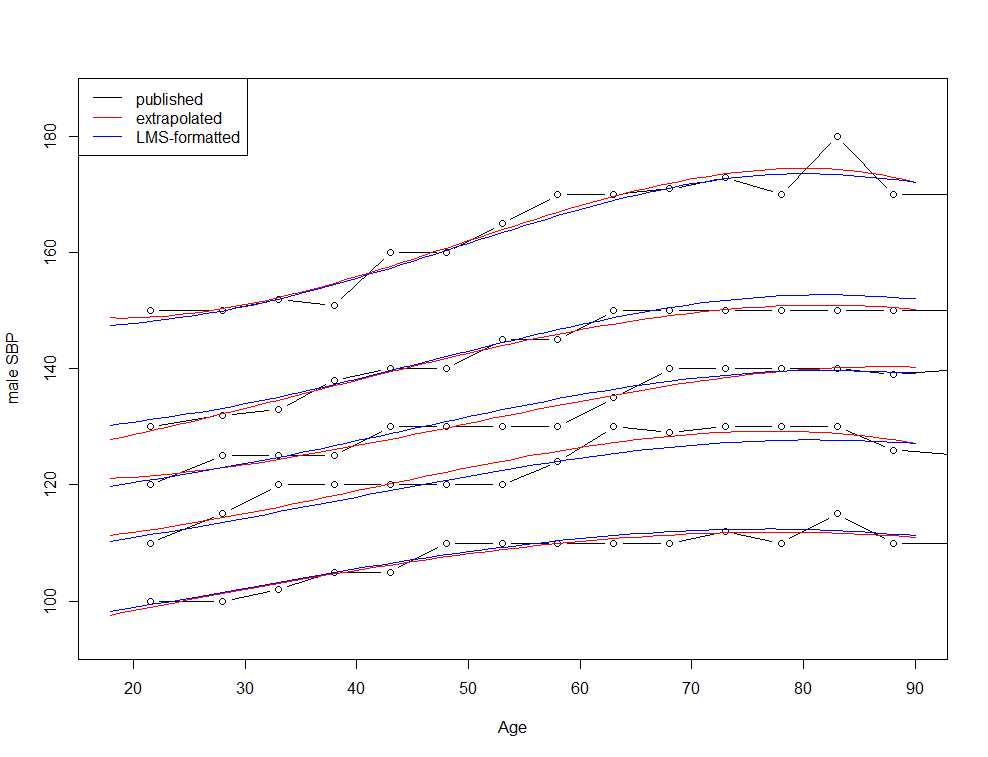


| **Table S3** – Increase of SBP depending on the increase of BMI [5] | | |
| --- | --- | --- |
| BMI Increase | Increase of SBP for women | Increase of SBP for men |
| >=20 to <25 | 7,6 | 3,8 |
| >=25 to <30 | 4,8 | 2,7 |
| >=30 to <35 | 2 | 1,7 |


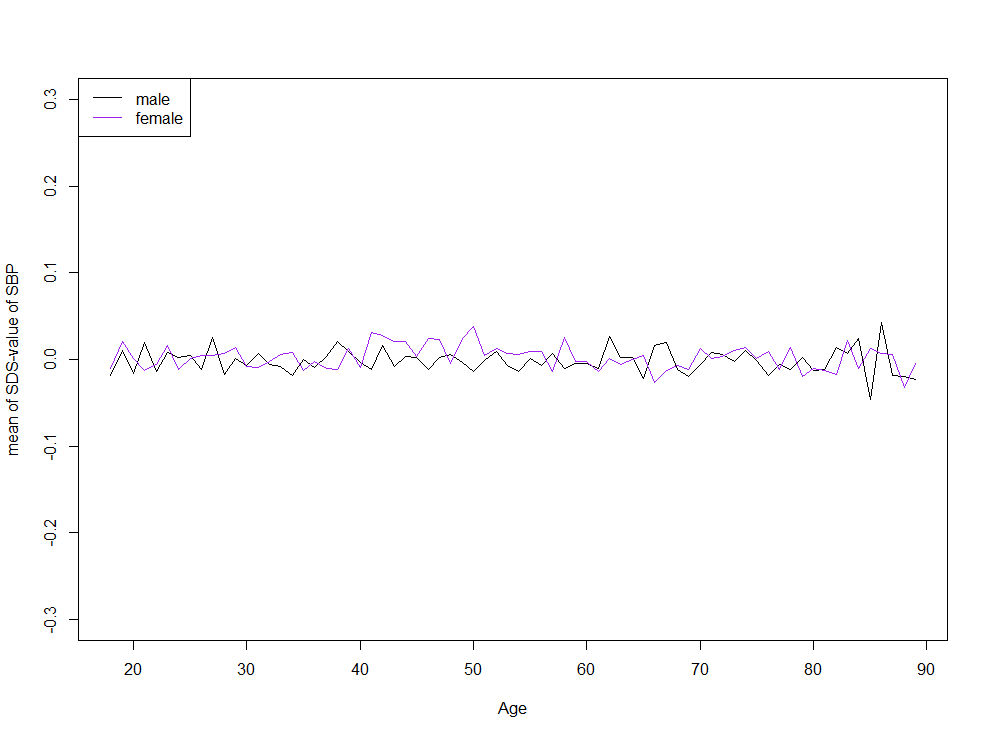


**Fig S5** – Mean of SDS-values of SBP at start of a simulation of 1 million individuals

| **Table S4** – German SBP percentiles [4] converted to LMS-Format and mean values for generating SDS-values when combined with BMI-depending increase | | | | | | | | |
| --- | --- | --- | --- | --- | --- | --- | --- | --- |
| Age | Men | | | Women | | | Mean used for  generating a normally  distributed SDS value  for men | Mean used for  generating a normally  distributed SDS value  for women |
|  | Lambda | Mu | Sigma | Lambda | Mu | Sigma |  |  |
| 18 | -0.202841376123964 | 119.806632600881 | 0.123644089044203 | 2.03581641622003 | 116.497152588036 | 0.0917963211996379 | -0.165797707124811 | -0.370484129024525 |
| 19 | -0.202197514052263 | 120.097897377293 | 0.123029784535853 | 1.85771341297017 | 116.390732551046 | 0.0936167382118071 | -0.175930300041522 | -0.373558115711263 |
| 20 | -0.201583279932025 | 120.390286041321 | 0.122419700936935 | 1.68031494161921 | 116.289328109327 | 0.0954295388981162 | -0.185630131047518 | -0.376673000285778 |
| 21 | -0.201041292617595 | 120.685273917849 | 0.121819727550185 | 1.50461093681351 | 116.199509339233 | 0.0972244827317283 | -0.19490572348862 | -0.379826619640114 |
| 22 | -0.200625004850552 | 120.984431325379 | 0.121236927855777 | 1.33181683190942 | 116.128278253022 | 0.0989900922517616 | -0.203765600710647 | -0.383016810666311 |
| 23 | -0.200389742684284 | 121.289263340306 | 0.120678495031546 | 1.16318711940679 | 116.082363088908 | 0.100715062201266 | -0.212218286059421 | -0.386241410256414 |
| 24 | -0.200389559588234 | 121.600975040368 | 0.12015123976567 | 0.999955306476906 | 116.067217318508 | 0.102389593480145 | -0.22027230288076 | -0.389498255302462 |
| 25 | -0.200672927585966 | 121.920482290464 | 0.119661219999623 | 0.843258494801927 | 116.087073831168 | 0.104005641804199 | -0.227936174520484 | -0.392785182696498 |
| 26 | -0.201276219582697 | 122.248292602571 | 0.119213137886809 | 0.694038457029642 | 116.144462128863 | 0.105557902149695 | -0.235218424324413 | -0.396100029330563 |
| 27 | -0.202229365655216 | 122.58473818831 | 0.118811034449413 | 0.553135944078131 | 116.241185528142 | 0.107042313733523 | -0.242127575638367 | -0.3994406320967 |
| 28 | -0.203547065496585 | 122.929813672147 | 0.118457495529416 | 0.421157553538329 | 116.377659231156 | 0.108457367161952 | -0.248672151808166 | -0.402804827886953 |
| 29 | -0.205233209256236 | 123.283339572666 | 0.118154190329707 | 0.298550348889463 | 116.553594493118 | 0.109803003439723 | -0.254860676179629 | -0.406190453593361 |
| 30 | -0.207276539848663 | 123.644955413509 | 0.117901634175686 | 0.185543639095256 | 116.767985361422 | 0.111080795999455 | -0.260701672098575 | -0.409595346107966 |
| 31 | -0.209659119178558 | 124.014234519132 | 0.117699861737985 | 0.0822709063041306 | 117.019567310453 | 0.112292939913192 | -0.266203662910826 | -0.413017342322811 |
| 32 | -0.212349926695088 | 124.390652465928 | 0.1175480317954 | -0.0113213776089478 | 117.306704653502 | 0.11344262088955 | -0.271375171962201 | -0.416454279129938 |
| 33 | -0.215310478891283 | 124.773650119139 | 0.117444855503348 | -0.0953937046778421 | 117.627641328314 | 0.114533401258298 | -0.27622472259852 | -0.41990399342139 |
| 34 | -0.218493741453005 | 125.162644653375 | 0.117388572020416 | -0.170233235513594 | 117.98055754217 | 0.115569065879196 | -0.280760838165601 | -0.423364322089206 |
| 35 | -0.221849457704718 | 125.557047742044 | 0.117377246997229 | -0.236177690400468 | 118.363626147938 | 0.116553423833906 | -0.284992042009265 | -0.42683310202543 |
| 36 | -0.225322329873064 | 125.956272408034 | 0.117408688329647 | -0.293651255173327 | 118.775056312031 | 0.117490158396158 | -0.288926857475331 | -0.430308170122103 |
| 37 | -0.228855245364162 | 126.359737264724 | 0.1174806116857 | -0.34311851653089 | 119.21310167449 | 0.118382753679395 | -0.29257380790962 | -0.433787363271267 |
| 38 | -0.232390489269637 | 126.766869240953 | 0.117590679048252 | -0.38508083977298 | 119.676082835289 | 0.119234318599874 | -0.295941416657952 | -0.437268518364965 |
| 39 | -0.235870686058372 | 127.17709928396 | 0.117736547604797 | -0.420050454114786 | 120.162351694047 | 0.120047769969057 | -0.299038207066145 | -0.440749472295238 |
| 40 | -0.239240515164817 | 127.589867257313 | 0.117915909939641 | -0.448548352969223 | 120.670326354198 | 0.120825585973857 | -0.301872702480021 | -0.444228061954129 |
| 41 | -0.242447005519435 | 128.004616365172 | 0.11812650561679 | -0.471091194634833 | 121.198459192233 | 0.121569951851813 | -0.304453426245399 | -0.447702124233681 |
| 42 | -0.245441581212935 | 128.420790740079 | 0.118366156747022 | -0.488178672135185 | 121.745236581576 | 0.122282659667127 | -0.306788901708097 | -0.451169496025931 |
| 43 | -0.248178003415439 | 128.837834766418 | 0.118632728705536 | -0.50029940450782 | 122.309158803944 | 0.122965327915079 | -0.308887652213936 | -0.454628014222925 |
| 44 | -0.250615517323583 | 129.255192958471 | 0.118924187645846 | -0.507912047270293 | 122.888750799233 | 0.123619235254858 | -0.310758201108738 | -0.458075515716706 |
| 45 | -0.252717277058153 | 129.672308054728 | 0.119238564706186 | -0.511451781141145 | 123.482545688335 | 0.124245467988677 | -0.312409071738319 | -0.461509837399312 |
| 46 | -0.254452104100946 | 130.088618365308 | 0.119573976417322 | -0.51131757169409 | 124.089079242155 | 0.124844885099649 | -0.3138487874485 | -0.464928816162788 |
| 47 | -0.255791457227854 | 130.503560597476 | 0.119928579051954 | -0.507890637852979 | 124.706888538474 | 0.125418252226441 | -0.315085871585103 | -0.468330288899175 |
| 48 | -0.256712264644105 | 130.916569089098 | 0.120300611256762 | -0.50151345036906 | 125.334513364147 | 0.12596616894985 | -0.316128847493945 | -0.471712092500515 |
| 49 | -0.257194834412446 | 131.327076017812 | 0.120688359180474 | -0.492503167379265 | 125.970491550442 | 0.126489152849516 | -0.316986238520847 | -0.475072063858849 |
| 50 | -0.25722378230978 | 131.734509893705 | 0.121090167191013 | -0.481142398256249 | 126.613353971043 | 0.126987637274323 | -0.317666568011628 | -0.478408039866221 |
| 51 | -0.256785556971817 | 132.138298268839 | 0.121504406552284 | -0.467697944980419 | 127.261629490177 | 0.127462024048205 | -0.318178359312108 | -0.481717857414671 |
| 52 | -0.255870034272419 | 132.537868310021 | 0.121929503671339 | -0.452404460688746 | 127.913845383699 | 0.127912666785907 | -0.31853013576811 | -0.484999353396243 |
| 53 | -0.254468867373475 | 132.932646707956 | 0.122363915876218 | -0.435477208443092 | 128.568526001398 | 0.128339902233231 | -0.318730420725448 | -0.488250364702976 |
| 54 | -0.25257548439856 | 133.322059120477 | 0.122806137552744 | -0.417106638869796 | 129.224189117484 | 0.128744058915062 | -0.318787737529945 | -0.491468728226915 |
| 55 | -0.250183908178481 | 133.705531498732 | 0.123254680572556 | -0.397472257183007 | 129.879351083803 | 0.129125464705486 | -0.318710609527421 | -0.4946522808601 |
| 56 | -0.247288893539131 | 134.082492066388 | 0.123708093446094 | -0.376732235664695 | 130.532528812289 | 0.129484453292972 | -0.318507560063696 | -0.497798859494573 |
| 57 | -0.243885141087477 | 134.452370305699 | 0.124164944429842 | -0.355032580072135 | 131.182238339861 | 0.129821368265005 | -0.318187112484587 | -0.500906301022378 |
| 58 | -0.239966515722014 | 134.814597044998 | 0.124623823062005 | -0.332504456039634 | 131.826992667407 | 0.130136573192175 | -0.317757790135918 | -0.503972442335555 |
| 59 | -0.235526283878842 | 135.16860443824 | 0.125083328579238 | -0.309272624948112 | 132.465305096125 | 0.130430441609787 | -0.317228116363505 | -0.506995120326145 |
| 60 | -0.230556018481376 | 135.51382890284 | 0.125542079868291 | -0.285449710717495 | 133.095693262369 | 0.130703369244981 | -0.31660661451317 | -0.509972171886194 |
| 61 | -0.225045722875675 | 135.849709196049 | 0.125998702952658 | -0.261141699420797 | 133.716676303472 | 0.130955768268953 | -0.315901807930732 | -0.512901433907739 |
| 62 | -0.21898259765101 | 136.17568628808 | 0.126451823883618 | -0.236446718146607 | 134.326772630726 | 0.13118807581066 | -0.31512221996201 | -0.515780743282825 |
| 63 | -0.212352424104306 | 136.491202499704 | 0.126900067339055 | -0.211459488110051 | 134.92450130096 | 0.131400740181179 | -0.314276373952825 | -0.518607936903493 |
| 64 | -0.205137790504407 | 136.795703691546 | 0.127342052201496 | -0.186268257539916 | 135.508384949928 | 0.131594231930934 | -0.313372793248997 | -0.521380851661786 |
| 65 | -0.197318959597886 | 137.088636422439 | 0.127776385086172 | -0.160957693002842 | 136.076944727037 | 0.13176903652763 | -0.312420001196345 | -0.524097324449745 |
| 66 | -0.188872763646992 | 137.369444479281 | 0.128201642039271 | -0.1356079711377 | 136.628690861794 | 0.131925660935033 | -0.311426521140688 | -0.526755192159411 |
| 67 | -0.179774514008823 | 137.63756965153 | 0.128616383439935 | -0.110297151492521 | 137.16212713202 | 0.132064621421604 | -0.310400876427847 | -0.529352291682828 |
| 68 | -0.16999652097459 | 137.892448134569 | 0.129019133939198 | -0.0850988831710151 | 137.675741181479 | 0.132186451970838 | -0.309351590403643 | -0.531886459912038 |
| 69 | -0.159509356979038 | 138.133508136331 | 0.129408387761596 | -0.0600839208004577 | 138.167999893472 | 0.132291698586271 | -0.308287186413892 | -0.53435553373908 |
| 70 | -0.148281836534835 | 138.360158801565 | 0.129782591254824 | -0.0353185359345107 | 138.637322100311 | 0.132380925678153 | -0.307216187804418 | -0.536757350055998 |
| 71 | -0.136282341083194 | 138.571799139367 | 0.130140172369702 | -0.0108666109646944 | 139.082101227083 | 0.132454706015491 | -0.306147117921038 | -0.539089745754835 |
| 72 | -0.123479159243685 | 138.767804235946 | 0.130479528508346 | 0.0132135408160176 | 139.500671132525 | 0.132513629127231 | -0.305088500109573 | -0.541350557727632 |
| 73 | -0.109841501410307 | 138.947529395164 | 0.13079904697564 | 0.0368679152271749 | 139.891315536182 | 0.132558297734643 | -0.304048857715841 | -0.54353762286643 |
| 74 | -0.0953418238690664 | 139.11030163009 | 0.131097134867896 | 0.0600499224973197 | 140.252242148731 | 0.132589338283794 | -0.303036714085664 | -0.545648778063272 |
| 75 | -0.0799548499833022 | 139.255439345836 | 0.131372228229743 | 0.0827169491480388 | 140.581632124147 | 0.132607389257073 | -0.302060592564862 | -0.547681860210199 |
| 76 | -0.0636615982659363 | 139.382251780702 | 0.131622863883213 | 0.104835723800307 | 140.87762814983 | 0.132613116086694 | -0.301129016499252 | -0.549634706199255 |
| 77 | -0.0464490584197126 | 139.490058625076 | 0.13184769823073 | 0.126380272258153 | 141.138379336122 | 0.132607207275435 | -0.300250509234656 | -0.551505152922479 |
| 78 | -0.028315725533095 | 139.578245989624 | 0.132045668729151 | 0.147336801194193 | 141.362149450951 | 0.132590393532533 | -0.299433594116893 | -0.553291037271916 |
| 79 | -0.00926636751177587 | 139.646256979037 | 0.132215886422338 | 0.167697708496975 | 141.547311262254 | 0.132563426898889 | -0.298686794491784 | -0.554990196139606 |
| 80 | 0.0106792795330383 | 139.693706912321 | 0.132357912684608 | 0.187469339645146 | 141.692578644517 | 0.132527107291403 | -0.298018633705148 | -0.556600466417593 |
| 81 | 0.0314896261100929 | 139.720400673086 | 0.132471710710403 | 0.206668239639239 | 141.797052831584 | 0.132482272080732 | -0.297437635102802 | -0.558119684997914 |
| 82 | 0.053113905701983 | 139.726564525025 | 0.132557978618055 | 0.225326379411939 | 141.86071619147 | 0.132429817121122 | -0.296952322030571 | -0.559545688772617 |
| 83 | 0.0754922157681997 | 139.712669042818 | 0.132617795234745 | 0.243482820280897 | 141.88406899009 | 0.132370663316563 | -0.296571217834271 | -0.56087631463374 |
| 84 | 0.0985451677514743 | 139.679796980145 | 0.132653115284568 | 0.261191188807879 | 141.86892097689 | 0.132305779525936 | -0.296302845859722 | -0.562109399473326 |
| 85 | 0.122180826471665 | 139.629540462414 | 0.132666531319411 | 0.278513944995556 | 141.818184990486 | 0.132236158905784 | -0.296155729452746 | -0.563242780183418 |
| 86 | 0.146292953292275 | 139.564293175039 | 0.132661501536088 | 0.295522927021645 | 141.736537574209 | 0.132162799950595 | -0.296138391959161 | -0.564274293656057 |
| 87 | 0.170771142309092 | 139.486782114263 | 0.132641804539625 | 0.312292495567705 | 141.629401136707 | 0.132086686527264 | -0.296259356724786 | -0.565201776783285 |
| 88 | 0.195501571760544 | 139.40035590381 | 0.132611717152487 | 0.328898320715309 | 141.503619541123 | 0.132008735353436 | -0.296527147095444 | -0.566023066457145 |
| 89 | 0.220376663254256 | 139.308533080152 | 0.132575504972131 | 0.345411051620923 | 141.366483177666 | 0.131929764172608 | -0.296950286416952 | -0.566735999569678 |
| 90 | 0.24531553411344 | 139.214254732865 | 0.13253654903491 | 0.361882968849318 | 141.224162487461 | 0.131850352091563 | NA | NA |

## **Therapy of hypertension**

Individuals with an SBP exceeding 140 mmHg are considered hypertensive and receive treatment based on the drug distribution for anti-hypertensive therapy [6]. The assigned drug will be permanent, and getting no treatment is also an option. Because only Angiotensin-converting-enzyme inhibitors (ACEI) and Angiotensin-II-receptor blockers (ARB) have effects in the S-GASM, we decided only to assign these while all other medication is summarized to "others." Being treated with ACEI excludes being treated with ARB and vice versa. **Table S5** shows the values used in the model. **Table S6** shows the effects of the medication. Medication assigned because of hypertension is not dependent on adherence because the published data on the distribution of therapy do not consider it.

| **Table S5** – Distribution of Therapy caused by hypertension based on [6] | |
| --- | --- |
| Percent of individuals with hypertension getting treatment | 71.6 |
| Percent of treated individuals getting ACEI | 45 |
| Percent of treated individuals getting ARB | 30 |

| **Table S6** – Effects of Medication used in the S-GASM | |
| --- | --- |
| Reduced GFR loss due to ACEI or ARB[7] | 36% |
| The relative risk for the transition from micro- to macroalbuminuria due to ACEI or ARB[8] | 45% |
| The relative risk for death due to ACEI[8] when an individual has albuminuria | 79% |

## **Diabetes**

We decided to simulate the initial presence and incidence of Type 2 diabetes mellitus using age- and sex-dependent BMI prevalence and incidence data [9], multiplied with the relative risk for a BMI above 30 or hypertension [10]. The cited source only gives odds ratios, so we calculated relative risks out of it. **Table S7** shows the relative risks used. Because the relative risks are published out of the univariate analysis, only the higher risk applies to individuals with both criteria.

We expanded the prevalence and incidence data with regression for getting steady age-depended risk changes. Because of better results on analyzing residuals compared to 3^rd^-grade polynomial regression, we decided to use 9^th^-grade polynomial regression combined with linear regression for the ages 18-30. **Fig S6** shows initial values and regression.

When combining prevalence and incidence with relative risks, the S-GASM would overestimate both parameters. We measured this overestimation and lowered the chances for the final risk to match the original expanded data while taking the relative risks into account. **Table S8** shows the prevalence and incidence values used in the simulation. **Fig S7** shows the prevalence and incidence in a simulation for 1 million individuals with visible statistical fluctuations but values overall close to the previously extrapolated data.

We lowered the risks with the following considerations:

$$overall risk=\sum_{n=1}^{k} ({base risk\times relative risk}_{n}\times{probability of occurence}_{n})$$

Even without knowing the exact probabilities of occurrences or specific relative risks, we could say:

$$overall risk=base risk\times\sum_{n=1}^{k} {(relative risk}_{n}\times{probability of occurence}_{n})$$

When we use a specific risk (used risk) and measure an overall risk higher than the risk we wanted, we calculate a factor of overestimation (foo):

with $overall risk=wanted risk \times foo$ and $base risk=used risk$

$$wanted risk \times foo =used risk\times\sum_{n=1}^{k} {(relative risk}_{n}\times{probability of occurence}_{n})$$

We are now able to transform to:

$$wanted risk=\frac{used risk}{foo}\times\sum_{n=1}^{k} {(relative risk}_{n}\times{probability of occurence}_{n})\mathrm{with}\frac{used risk}{foo}=risk to use$$

We measured the overestimation for every parameter and every age with these considerations and reduced the risk accordingly.

| **Table S7** – Relative Risks for Diabetes calculated from published odds ratios | |
| --- | --- |
| Relative Risk with BMI>30 | 2,319 |
| Relative Risk with Hypertension | 2,048 |

| **Table S8** – Prevalence and incidence of Diabetes used in S-GASM (for combination with relative risks) | | | | |
| --- | --- | --- | --- | --- |
| Age | Prevalence for men | Prevalence for women | Incidence for men | Incidence for women |
| 18 | 0.000959510078498551 | 0.00115044796040089 | 0.000165733333333333 | 0.000173533333333333 |
| 19 | 0.000997956960423156 | 0.00137778706274571 | 0.000175133333333333 | 0.000183233333333333 |
| 20 | 0.0010679483840781 | 0.00154809026958036 | 0.000184533333333333 | 0.000192933333333333 |
| 21 | 0.00115403469947364 | 0.00169491886816975 | 0.000193933333333333 | 0.000202633333333333 |
| 22 | 0.00123110059051695 | 0.0018629904797834 | 0.000203333333333333 | 0.000212333333333333 |
| 23 | 0.00131634308284067 | 0.00205065124489279 | 0.000212733333333333 | 0.000222033333333333 |
| 24 | 0.00139189227633914 | 0.00223892525196449 | 0.000222133333333333 | 0.000231733333333333 |
| 25 | 0.00145671286574674 | 0.00242977197817121 | 0.000231533333333333 | 0.000241433333333333 |
| 26 | 0.00154423476982684 | 0.0025710684889856 | 0.000240933333333333 | 0.000251133333333333 |
| 27 | 0.00164273233570301 | 0.00273906900985722 | 0.000250333333333333 | 0.000260833333333333 |
| 28 | 0.00174192766060885 | 0.00293049527973934 | 0.000259733333333333 | 0.000270533333333333 |
| 29 | 0.00182631397568753 | 0.00307105328450179 | 0.000269133333333333 | 0.000280233333333333 |
| 30 | 0.00308782365877027 | 0.00569682475021598 | 0.00036609174047437 | 0.000453056557795781 |
| 31 | 0.00349445276291924 | 0.00553790194367617 | 0.000317899820000904 | 0.000498880722897477 |
| 32 | 0.00382351914524611 | 0.0052717459149276 | 0.00041121653018077 | 0.000536016308214478 |
| 33 | 0.0041353439509977 | 0.00495559492421957 | 0.000498514607097428 | 0.000559314794489107 |
| 34 | 0.00439856486801363 | 0.00460109032579775 | 0.000569801670773161 | 0.000565072158935957 |
| 35 | 0.00466488262934672 | 0.00430754908348053 | 0.000625635376391275 | 0.000560594155177776 |
| 36 | 0.00494960624259441 | 0.0040847532485086 | 0.000663330227164691 | 0.000544011900015103 |
| 37 | 0.00529386693730113 | 0.00396719626117108 | 0.000685163803038577 | 0.000518542489057944 |
| 38 | 0.00580081199275761 | 0.00402539589395086 | 0.000702970532977264 | 0.00049436326587109 |
| 39 | 0.00647455752371009 | 0.00422759811688537 | 0.000717017764382009 | 0.000469725539481112 |
| 40 | 0.00738090369459066 | 0.00459747213785331 | 0.000736385757954956 | 0.000450745626950411 |
| 41 | 0.00845675819275057 | 0.00512907148422679 | 0.000758035385616085 | 0.000440198533283142 |
| 42 | 0.00974077658742872 | 0.00585275659969188 | 0.000790377112948308 | 0.000443658725936127 |
| 43 | 0.01131468978136 | 0.00674417015592706 | 0.000843126932898516 | 0.000461425753087982 |
| 44 | 0.0131040977029946 | 0.00780886302728629 | 0.000912439587076843 | 0.000495822477771934 |
| 45 | 0.0151357807992221 | 0.00909819065036772 | 0.0010017698538044 | 0.000551122460687594 |
| 46 | 0.0174373412788095 | 0.0105072738141416 | 0.00111337845869991 | 0.000621454378159261 |
| 47 | 0.0201384031377926 | 0.0120339269392177 | 0.00125505950544583 | 0.000706973774924 |
| 48 | 0.0231382747340712 | 0.0137630302315163 | 0.00141953795466954 | 0.000812294560209179 |
| 49 | 0.0262417172979653 | 0.0156096482822911 | 0.00159395343189014 | 0.000931663460001818 |
| 50 | 0.0296967282981438 | 0.0174593828039055 | 0.00179216968575461 | 0.0010570124828517 |
| 51 | 0.0334888448504644 | 0.019425980031063 | 0.00201162249953643 | 0.00119354003663164 |
| 52 | 0.037321738186868 | 0.021438034071265 | 0.00223280144807385 | 0.00133503661629332 |
| 53 | 0.0412469922655429 | 0.0234817665222422 | 0.00245716700497319 | 0.0014784991144895 |
| 54 | 0.0451314031084953 | 0.0255417548633317 | 0.00267525199142346 | 0.00162070872163548 |
| 55 | 0.0494094619829948 | 0.0276661353174226 | 0.00291142808231039 | 0.00176253693548105 |
| 56 | 0.0536232700183824 | 0.0298930656812969 | 0.0031374729249244 | 0.00190444311663417 |
| 57 | 0.0579010597210681 | 0.0320480687825619 | 0.00336030367806583 | 0.00203365375834995 |
| 58 | 0.0621435322009233 | 0.034435570661891 | 0.0035739601607572 | 0.00216827838430778 |
| 59 | 0.0660728589143807 | 0.0368009302775159 | 0.00376302044874979 | 0.00229150256060356 |
| 60 | 0.0700587878203753 | 0.0392652448489518 | 0.00394974318964855 | 0.00241093274608686 |
| 61 | 0.0737321238063676 | 0.0417662143728873 | 0.00411483579033954 | 0.00252339063933729 |
| 62 | 0.0774735224608605 | 0.0443775701686358 | 0.00428176424330418 | 0.00263471375023565 |
| 63 | 0.0807407235756113 | 0.0470307514386912 | 0.00442308464909069 | 0.00274278292217097 |
| 64 | 0.0838799084523109 | 0.0497535388730415 | 0.00456098996472843 | 0.00285184563207298 |
| 65 | 0.0869730220209792 | 0.0524537421891347 | 0.00470301853684576 | 0.00295971253187267 |
| 66 | 0.0895416146965034 | 0.0553225028871983 | 0.0048265484263913 | 0.0030805504226797 |
| 67 | 0.0918557015343022 | 0.05823878826724 | 0.00494934254099513 | 0.00321094692778294 |
| 68 | 0.0940141160358677 | 0.0614040301264296 | 0.00507943028306368 | 0.00336545578148509 |
| 69 | 0.0960012797139662 | 0.0643520893976693 | 0.00521812986694255 | 0.00352187979872452 |
| 70 | 0.0976984891972227 | 0.0673028507652189 | 0.00536032174107899 | 0.00369539968346327 |
| 71 | 0.0994607556064594 | 0.0704454651534127 | 0.00552588475864508 | 0.00389910773878639 |
| 72 | 0.101040762574419 | 0.0735914845845257 | 0.00570062488033605 | 0.00412496170033187 |
| 73 | 0.102568351579913 | 0.0765665650872595 | 0.00588989058158098 | 0.00436474180938587 |
| 74 | 0.10426573343948 | 0.0793990149253699 | 0.00610360230526241 | 0.00462059472817754 |
| 75 | 0.105934850710448 | 0.0822335303635875 | 0.00632633371441145 | 0.00490088682885823 |
| 76 | 0.107494029044303 | 0.0850299159198938 | 0.00654766577034088 | 0.00520271991600188 |
| 77 | 0.108832011787349 | 0.08751741396074 | 0.00675404660037594 | 0.00550762655151222 |
| 78 | 0.110052437564966 | 0.0896993984419048 | 0.00694457319520777 | 0.00581200037403899 |
| 79 | 0.111781416831976 | 0.0916729314566191 | 0.00715242181368967 | 0.00611751848128476 |
| 80 | 0.114151340367067 | 0.0933315812783045 | 0.00738151875533069 | 0.00641196224850662 |
| 81 | 0.115960267242141 | 0.0950088639559998 | 0.00755018033056708 | 0.00671331166423434 |
| 82 | 0.117245345841997 | 0.0965995248015918 | 0.00765862921476149 | 0.00701127452834682 |
| 83 | 0.118695284553535 | 0.0979031175078075 | 0.00775495255328385 | 0.00729018729404685 |
| 84 | 0.119932239037744 | 0.0989704125667199 | 0.00782468616012527 | 0.00755670176647087 |
| 85 | 0.120356447510264 | 0.0998894926557858 | 0.00784914862911072 | 0.00782775747673829 |
| 86 | 0.120736357096585 | 0.101048087131139 | 0.00791361417547078 | 0.0081540531799557 |
| 87 | 0.119538635440736 | 0.101855635318083 | 0.00797296644831613 | 0.00851763014269325 |
| 88 | 0.117543784518394 | 0.103501961764567 | 0.0081608662616791 | 0.00905112196705719 |
| 89 | 0.1148437847105 | 0.105115319849367 | 0.00860870322782829 | 0.00969534045768592 |
| 90 | 0.137288654782546 | 0.127788648881821 | 0.00877078509352468 | 0.0102780337906152 |

**Fig S7** – Prevalence (top) and incidence (bottom) of diabetes mellitus type 2 for females (left) and males (right) with previously extrapolated and measured data out of a simulation of 1 million individuals


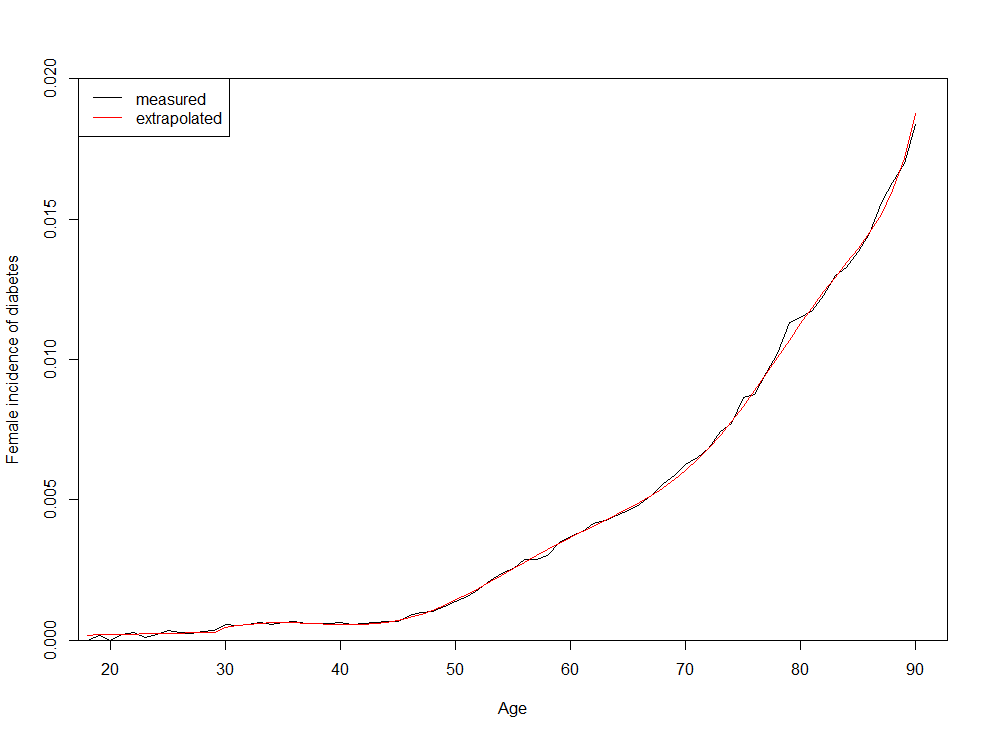

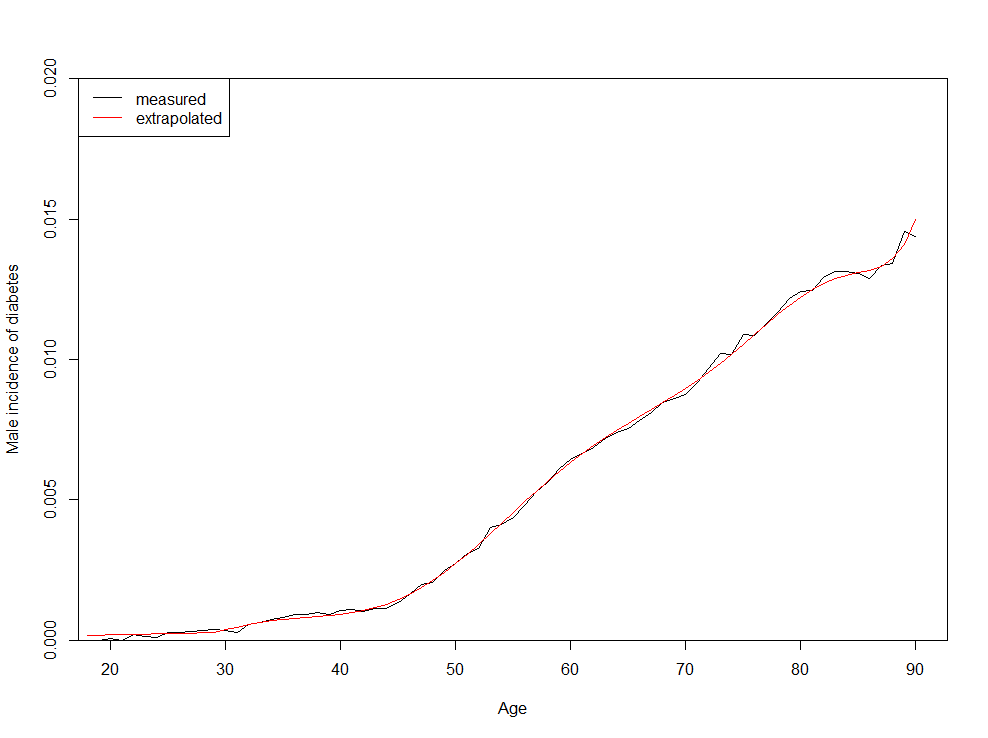

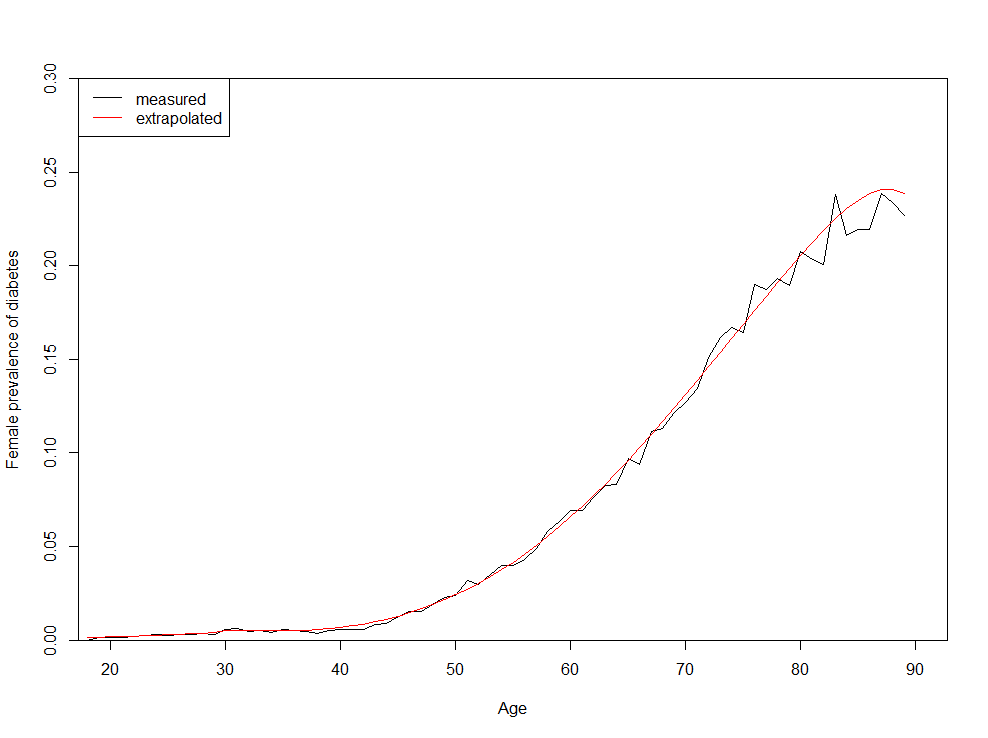

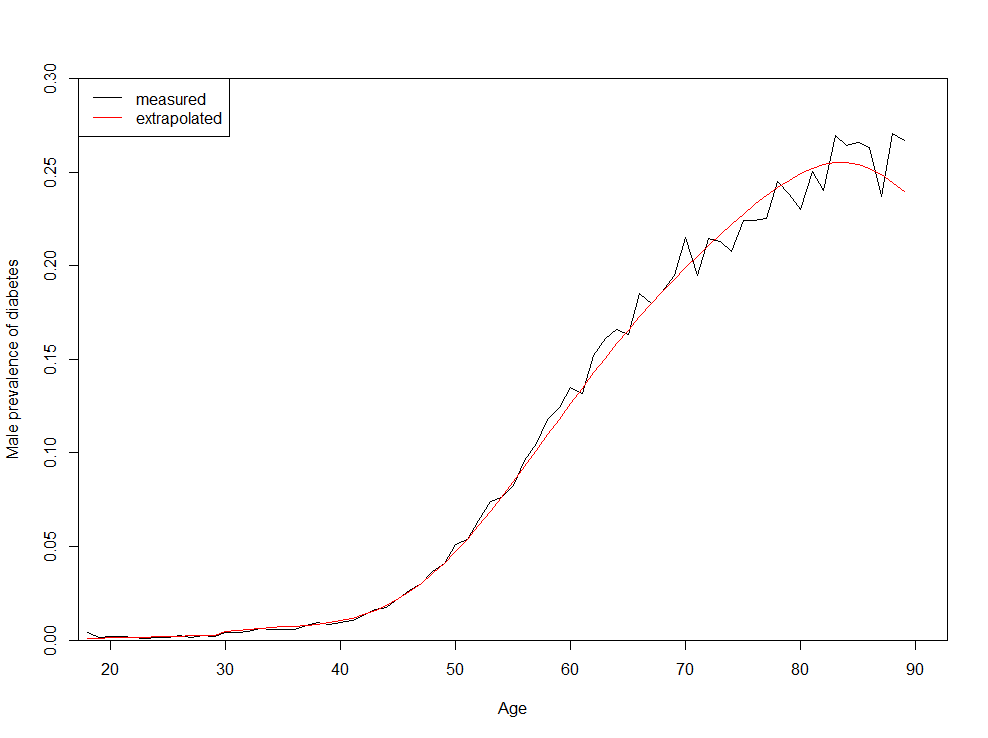

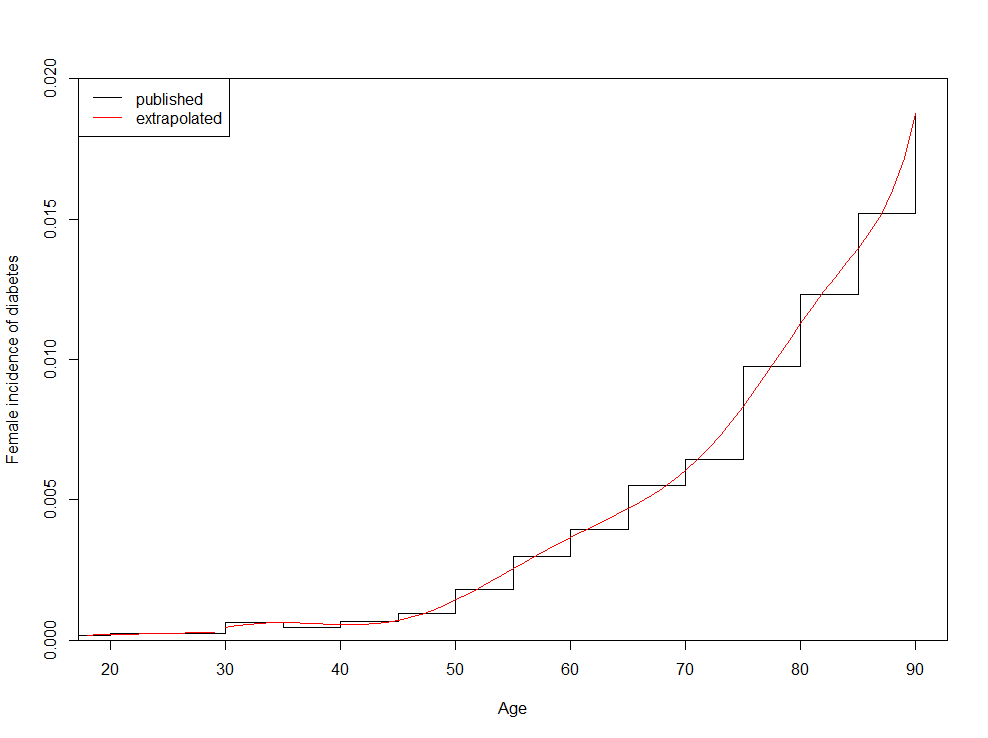

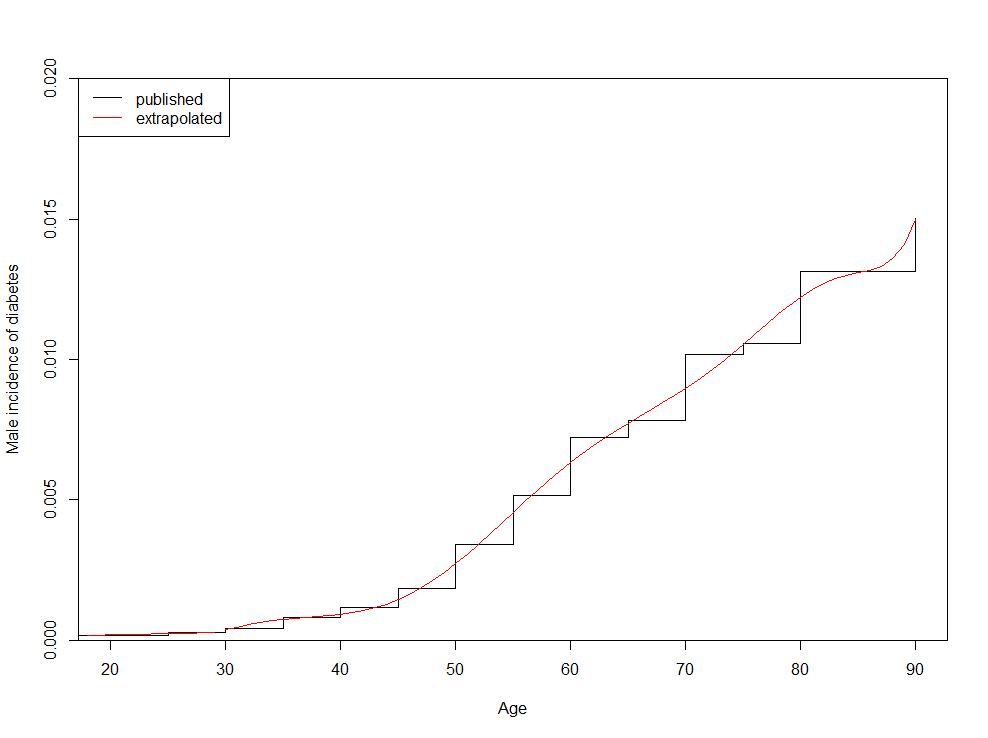

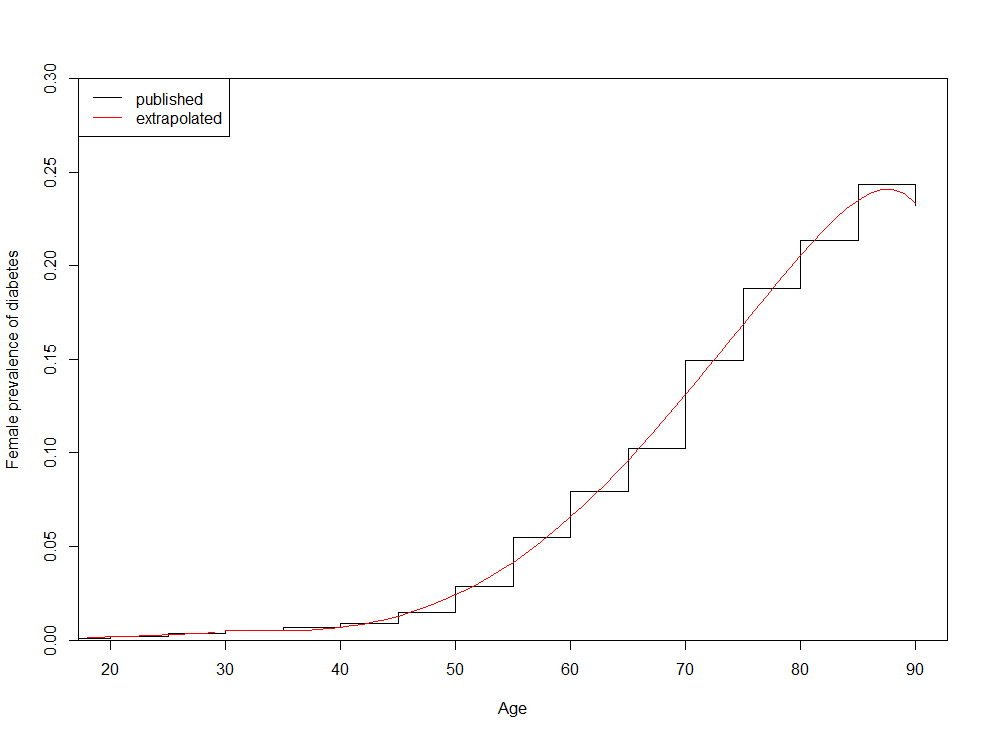

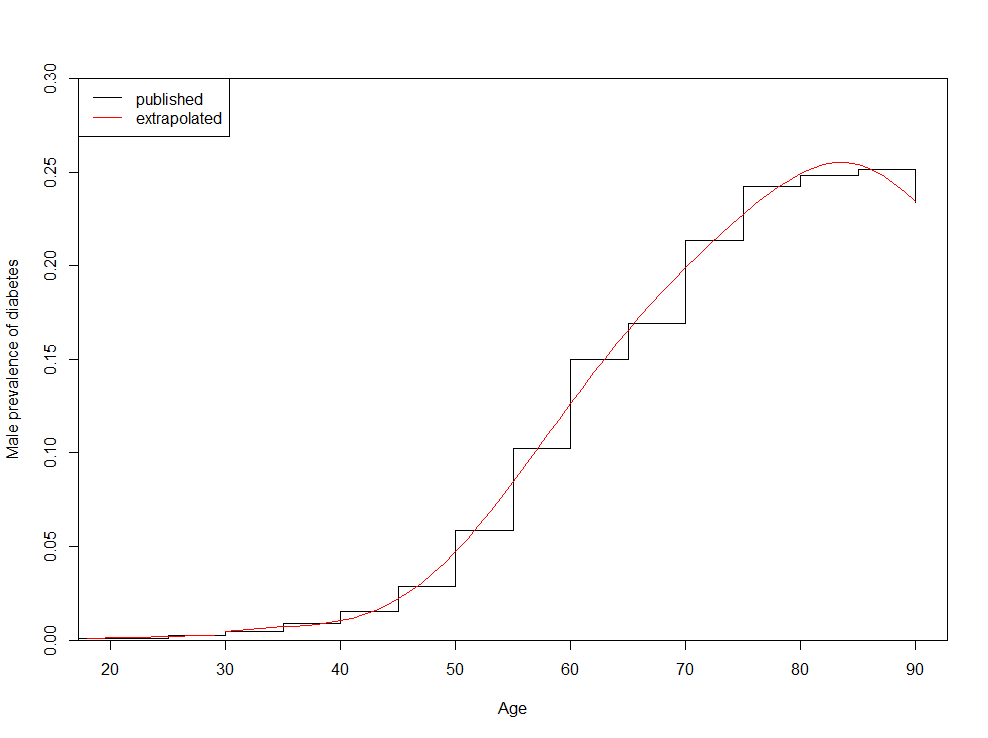


**Fig S6** – Prevalence (top) and incidence (bottom) of diabetes mellitus type 2 for females (left) and males (right) with published and extrapolated data

## **Albuminuria and glomerular filtration rate (GFR)**

We could not find suitable data from Germany for the simulation of albuminuria and GFR and had, therefore, to use data from a US-American simulation model [11], which can be found in the associated supplementary material of the cited source. Their data are based on the National Health and Nutrition Examination Survey (NHANES).

These data include prevalences for the age of 30 and incidences from 31-90 for albuminuria, depending on BMI and hypertension. We simulated 1 million individuals with the initial age of 30 years and derived age-, sex-, BMI-, and hypertension-dependent own prevalence data. We assumed for our model that there is no incidence for albuminuria for the ages 18-30, and the prevalences in the ages 18-29 match the prevalence given for the age of 30. With these assumptions, we derived the data for albuminuria we use in the S-GASM shown in **Table S9**.

The cited US-American simulation model includes the data for the annual GFR-loss we took over to the S-GASM, shown in **Table S10**. Also, we took over the values for the incidence and progression of albuminuria. As these data include values for the incidence of albuminuria for individuals older than 30, we assume no incidence or progression to happen before. The data used for the incidence and progression of albuminuria are presented in **Table S11**.

We used the data for GFR loss and the data provided for the mean and standard deviation of GFR for the age of 30 to derive own mean values and standard deviations for older age groups depending on age, sex, hypertension, diabetes, and albuminuria by simulating 1 million individuals starting with the age of 30. We assumed the mean and standard deviation for younger age groups to match the provided values for the age of 30. **Table S12** shows the values the S-GASM uses to assign a GFR at the start of a simulation.

Part of the U.S. simulation is an individual factor for the annual loss of GFR generated out of a triangular distribution from 0 to 2 with a mean of 1. We use this approach also in our model.

We tried to validate the S-GASM with all the data we described and noticed a high overestimation of the incidence of renal replacement therapy. We then decided to adjust the simulation by adjusting the individual factor for the annual loss of GFR. That would not impact the distribution of all risk factors and the relative differences in loss of GFR depending on risk factors. We tested a reduction of this factor in steps of 5% and found the best results in a reduction to 75% when comparing the incidence of renal replacement therapy. **Fig S8** shows the incidence of renal replacement therapy for these steps in comparison to the data of Icks et al.[12]

Every individual with a GFR below 7 ml/min will get renal replacement therapy as dialysis treatment.[13] The dialysis will not impact any factor besides the cost of dialysis.

| **Table S10** – annual GFR decrement [11] | | |
| --- | --- | --- |
| **Diabetes/Hypertension/Albuminuria Status** | **GFR** | **Annual GFR decrement** |
| No diabetes, no hypertension, no albuminuria | ≥ 60 | 0,653 |
|  | < 60 | 0,653 |
| No diabetes, no hypertension, with albuminuria | ≥ 60 | 0,719 |
|  | < 60 | 4,200 |
| No diabetes, with hypertension, no albuminuria | ≥ 60 | 0,719 |
|  | < 60 | 1,400 |
| No diabetes, with hypertension, with albuminuria | ≥ 60 | 0,784 |
|  | < 60 | 3,900 |
| With diabetes, no albuminuria | ≥ 60 | 1,100 |
|  | < 60 | 2,800 |
| With diabetes, with albuminuria | ≥ 60 | 4,100 |
|  | < 60 | 5,200 |


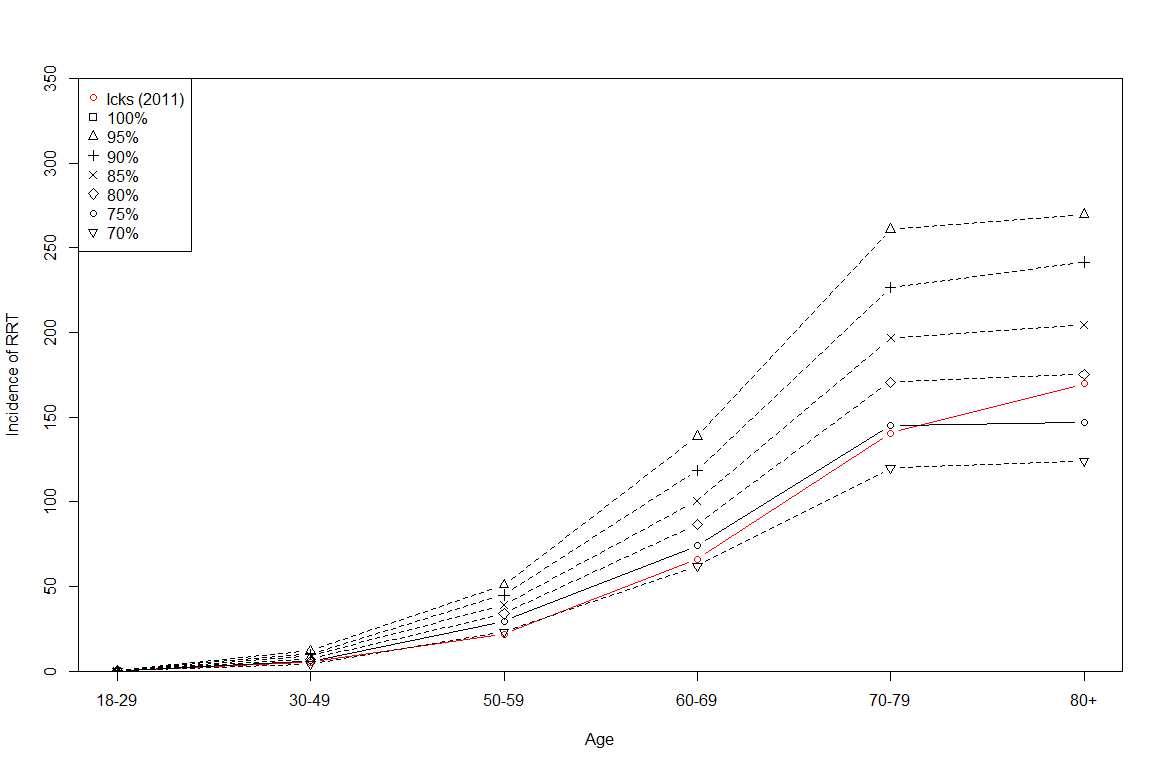


**Fig S8** – Incidence of renal replacement therapy per 100.000 while reducing overall annual loss of GFR in steps of 5% in a simulation of 1 million individuals and comparison to published data by Icks et al.[12]

| **Table S9** – Prevalence of Micro- and Macroalbuminuria used in S-GASM | | | | | | | | | | | | |
| --- | --- | --- | --- | --- | --- | --- | --- | --- | --- | --- | --- | --- |
| **Age** | **Microalbuminuria** | | | | | | **Macroalbuminuria** | | | | | |
|  | **No diabetes, no hypertension** | | **Hypertension** | | **Diabetes** | | **No diabetes, no hypertension** | | **Hypertension** | | **Diabetes** | |
|  | **male** | **female** | **male** | **female** | **male** | **female** | **male** | **female** | **male** | **female** | **male** | **female** |
| 18 | 0.002085 | 0.006918 | 0.010476 | 0.033855 | 0.009675 | 0.071624 | 0.000011 | 0.000011 | 0.000011 | 0.000011 | 0.000011 | 0.000011 |
| 19 | 0.002085 | 0.006918 | 0.010476 | 0.033855 | 0.009675 | 0.071624 | 0.000011 | 0.000011 | 0.000011 | 0.000011 | 0.000011 | 0.000011 |
| 20 | 0.002085 | 0.006918 | 0.010476 | 0.033855 | 0.009675 | 0.071624 | 0.000011 | 0.000011 | 0.000011 | 0.000011 | 0.000011 | 0.000011 |
| 21 | 0.002085 | 0.006918 | 0.010476 | 0.033855 | 0.009675 | 0.071624 | 0.000011 | 0.000011 | 0.000011 | 0.000011 | 0.000011 | 0.000011 |
| 22 | 0.002085 | 0.006918 | 0.010476 | 0.033855 | 0.009675 | 0.071624 | 0.000011 | 0.000011 | 0.000011 | 0.000011 | 0.000011 | 0.000011 |
| 23 | 0.002085 | 0.006918 | 0.010476 | 0.033855 | 0.009675 | 0.071624 | 0.000011 | 0.000011 | 0.000011 | 0.000011 | 0.000011 | 0.000011 |
| 24 | 0.002085 | 0.006918 | 0.010476 | 0.033855 | 0.009675 | 0.071624 | 0.000011 | 0.000011 | 0.000011 | 0.000011 | 0.000011 | 0.000011 |
| 25 | 0.002085 | 0.006918 | 0.010476 | 0.033855 | 0.009675 | 0.071624 | 0.000011 | 0.000011 | 0.000011 | 0.000011 | 0.000011 | 0.000011 |
| 26 | 0.002085 | 0.006918 | 0.010476 | 0.033855 | 0.009675 | 0.071624 | 0.000011 | 0.000011 | 0.000011 | 0.000011 | 0.000011 | 0.000011 |
| 27 | 0.002085 | 0.006918 | 0.010476 | 0.033855 | 0.009675 | 0.071624 | 0.000011 | 0.000011 | 0.000011 | 0.000011 | 0.000011 | 0.000011 |
| 28 | 0.002085 | 0.006918 | 0.010476 | 0.033855 | 0.009675 | 0.071624 | 0.000011 | 0.000011 | 0.000011 | 0.000011 | 0.000011 | 0.000011 |
| 29 | 0.002085 | 0.006918 | 0.010476 | 0.033855 | 0.009675 | 0.071624 | 0.000011 | 0.000011 | 0.000011 | 0.000011 | 0.000011 | 0.000011 |
| 30 | 0.002085 | 0.006918 | 0.010476 | 0.033855 | 0.009675 | 0.071624 | 0.000011 | 0.000011 | 0.000011 | 0.000011 | 0.000011 | 0.000011 |
| 31 | 0.00779486879251786 | 0.0123711416771947 | 0.0153840999120795 | 0.0371537252399708 | 0.0219817684328972 | 0.0782278715676303 | 0.0000850687720517899 | 0.000069230536475843 | 0.000374449469754254 | 0.000577797265153129 | 0.00200376911619916 | 0.00227508822245329 |
| 32 | 0.0120439434847615 | 0.0165876683032086 | 0.0196738711015186 | 0.0400762463780728 | 0.0294140643001353 | 0.0800956013951183 | 0.00040072130166845 | 0.000381195763846103 | 0.00113592083746988 | 0.00132625215526043 | 0.00469149990915188 | 0.00542758006823222 |
| 33 | 0.0169450237230452 | 0.0215107127057689 | 0.0247345512629011 | 0.0435320252557627 | 0.037027036029194 | 0.0822428753722215 | 0.00112393855449619 | 0.00081908557878763 | 0.00216569349858485 | 0.00227771493836388 | 0.00802192874763184 | 0.00911186414982859 |
| 34 | 0.0221670013956462 | 0.0268237169597799 | 0.0302260966933239 | 0.0472770842130853 | 0.0441590104043961 | 0.0846926719690682 | 0.00204852345058462 | 0.00135582840322341 | 0.0034098345226911 | 0.00336732550445865 | 0.0116986324460328 | 0.0130034078227639 |
| 35 | 0.0273850305896573 | 0.0322072952449644 | 0.0357995334977428 | 0.0510670410161101 | 0.0503228839769833 | 0.0874139925107887 | 0.00311930207131958 | 0.0019549188928489 | 0.00479969292231685 | 0.00451824370753535 | 0.0154083988639982 | 0.0168086271778507 |
| 36 | 0.032324340429182 | 0.0373822182506783 | 0.0411440861020673 | 0.0546926434407382 | 0.0552617960965871 | 0.0903151382206414 | 0.00427132269695561 | 0.00257649136440835 | 0.00626362144530655 | 0.00565309063590307 | 0.0188715222973358 | 0.020310472665975 |
| 37 | 0.0367886340209241 | 0.0421386444811704 | 0.0460201286978713 | 0.0580031332910742 | 0.0589565631704985 | 0.0932500240899319 | 0.00543861939514483 | 0.0031827116396208 | 0.00773653732882593 | 0.00670352623518775 | 0.0218771945493573 | 0.0233918050682449 |
| 38 | 0.0406722098866062 | 0.0463503940222729 | 0.0502765118475493 | 0.0609165557777004 | 0.0615928024733826 | 0.0960353532779626 | 0.00656145414090085 | 0.00374192209206809 | 0.00916645731373013 | 0.00761708131261806 | 0.0243017966853684 | 0.0260371329997034 |
| 39 | 0.0439574431270921 | 0.049975430416 | 0.0538533236116362 | 0.0634182522717882 | 0.0635002156285982 | 0.0984750028272252 | 0.00759153570157636 | 0.00423128202010557 | 0.0105177981977003 | 0.0083609262469125 | 0.0261109804960689 | 0.0283165589732407 |
| 40 | 0.0467009024436134 | 0.0530453180410644 | 0.0567728328875616 | 0.0655501722174459 | 0.0650782052917145 | 0.100387312108495 | 0.0084950818324251 | 0.00463789633351167 | 0.0117717293085151 | 0.00892270994386143 | 0.027348558059119 | 0.0303576942070132 |
| 41 | 0.0490121945416013 | 0.0556472756809262 | 0.0591222623562437 | 0.0673943411179619 | 0.066721358651332 | 0.101631019418695 | 0.00925386540576907 | 0.00495860870155319 | 0.0129241864481105 | 0.00930892335952118 | 0.0281163985586124 | 0.0323119701770259 |
| 42 | 0.051029736058055 | 0.0579026461417764 | 0.0610322574111603 | 0.069053922792487 | 0.0687559783567357 | 0.102126205706467 | 0.00986456488805342 | 0.0051987502667471 | 0.0139823195335829 | 0.00954143339031738 | 0.0285498747018507 | 0.0343214228977633 |
| 43 | 0.0528972149103517 | 0.0599452841384008 | 0.062654606396076 | 0.0706349507636859 | 0.071395440231107 | 0.101867599376544 | 0.0103368350583477 | 0.00537018839753523 | 0.0149601735858824 | 0.00965290856910582 | 0.0287930782949109 | 0.0364909330639928 |
| 44 | 0.0547436939094915 | 0.0619026856508721 | 0.0641420930205489 | 0.0722311256121761 | 0.0747183337554285 | 0.100928792595354 | 0.0106905405472717 | 0.00548902256947116 | 0.0158743271491159 | 0.00968183865790443 | 0.0289772336138103 | 0.0388693623714674 |
| 45 | 0.0566693015373836 | 0.0638817964876486 | 0.0656324865645778 | 0.0739132292904796 | 0.0786696332272503 | 0.0994571521843016 | 0.0109525703545961 | 0.00557323912694699 | 0.0167400678112008 | 0.00966776022533771 | 0.0292046762808997 | 0.041441305934687 |
| 46 | 0.0587374014434482 | 0.0659604867687569 | 0.0672377440900744 | 0.075723824981976 | 0.0830819731472426 | 0.0976603375940225 | 0.0111535934083434 | 0.00564057644968926 | 0.0175685035816805 | 0.00964716448556106 | 0.0295396144920341 | 0.0441295159798527 |
| 47 | 0.0609731592799115 | 0.0681847793622703 | 0.0690386292269979 | 0.0776770996376979 | 0.0877117463097536 | 0.0957862577729496 | 0.011325039006607 | 0.00570677904245696 | 0.018364819519584 | 0.00965040524725022 | 0.0300057985596158 | 0.0468066245691731 |
| 48 | 0.0633676260935589 | 0.0705711636732792 | 0.0710842306771435 | 0.0797630451988773 | 0.0922833591837786 | 0.0940989368252944 | 0.011496504335461 | 0.00578434363615423 | 0.0191277134933052 | 0.00969976383790302 | 0.0305903068496632 | 0.0493137274117247 |
| 49 | 0.0658858905505542 | 0.0731127710783803 | 0.0733953442956294 | 0.0819547135876529 | 0.0965345943161026 | 0.0928530797243578 | 0.0116937136010315 | 0.0058817906646697 | 0.0198498992005551 | 0.00980868037056321 | 0.0312519849897223 | 0.0514827538102634 |
| 50 | 0.0684775437737727 | 0.075787864488186 | 0.0759703883663938 | 0.0842170412645648 | 0.100256564647 | 0.092270131800653 | 0.0119370857719933 | 0.00600343618835151 | 0.0205194581037759 | 0.00998203831814029 | 0.0319326861382518 | 0.0531593531329726 |
| 51 | 0.0710876469442989 | 0.0785690045579444 | 0.0787924479490143 | 0.0865157174561073 | 0.103323036320981 | 0.0925183360662013 | 0.0122409136160914 | 0.00614959582894191 | 0.0211217584005668 | 0.010217299219611 | 0.0325693540095075 | 0.0542232434978539 |
| 52 | 0.0736665648417873 | 0.0814313782589965 | 0.0818361639923507 | 0.0888247407726798 | 0.10570572231006 | 0.0936987609239721 | 0.0126131162466453 | 0.00631712476077497 | 0.0216416372036179 | 0.0105062294699386 | 0.0331051391736734 | 0.0546035246553932 |
| 53 | 0.0761773812183797 | 0.0843590729089353 | 0.0850734528282831 | 0.0911316305106821 | 0.107474254006302 | 0.0958385647672881 | 0.0130554995218141 | 0.00650018564104954 | 0.0220655564970835 | 0.0108369409074538 | 0.0334980927416554 | 0.0542872595599673 |
| 54 | 0.0786000850412468 | 0.087348498656602 | 0.0884774079996182 | 0.0934396796092936 | 0.10878166045422 | 0.098891961016922 | 0.0135644409293157 | 0.00669113751281209 | 0.022383486213449 | 0.0111959777050036 | 0.0337264751273964 | 0.0533205684128998 |
| 55 | 0.0809322450960532 | 0.090408646190154 | 0.0920241415012461 | 0.095767099318174 | 0.109838081097797 | 0.102748528416406 | 0.0141319051042717 | 0.00688145016490422 | 0.02259032950754 | 0.0115702180849812 | 0.0337902793609961 | 0.0518024419734319 |
| 56 | 0.0831864094907571 | 0.0935583541085544 | 0.0956927096703182 | 0.0981433549890644 | 0.11087690195035 | 0.107247755145732 | 0.0147466900316155 | 0.00706256665786863 | 0.0226867762412403 | 0.0119484133980177 | 0.0337091268085982 | 0.0498723610971525 |
| 57 | 0.0853849174764568 | 0.0968211958623485 | 0.0994635914129551 | 0.100603377311816 | 0.112118385683001 | 0.112198081058393 | 0.0153958000421255 | 0.00722665811641632 | 0.0226795417760742 | 0.0123222512939857 | 0.0335171832270513 | 0.0476935155004192 |
| 58 | 0.0875531456438815 | 0.100218931733681 | 0.103316405649277 | 0.103180612574132 | 0.113736078109121 | 0.117398266015542 | 0.0160658386472341 | 0.00736723614189176 | 0.0225810118605482 | 0.0126868963434239 | 0.0332561120365678 | 0.0454338777549713 |
| 59 | 0.0897123952340746 | 0.10376467138273 | 0.107227647435793 | 0.105900020610128 | 0.115830803968948 | 0.12265870234437 | 0.0167443119509567 | 0.00747960662259667 | 0.0224083652611774 | 0.0130410235536001 | 0.0329672929438294 | 0.0432475669616515 |
| 60 | 0.0918736450581522 | 0.107456936191695 | 0.111169180747213 | 0.108772126252564 | 0.11841597348825 | 0.127820324266334 | 0.0174207328797643 | 0.007561162473459 | 0.0221822808255043 | 0.0133864120450988 | 0.0326845698537793 | 0.0412588190674391 |
| 61 | 0.0940332462591222 | 0.111275693445241 | 0.115108059228377 | 0.111789081240445 | 0.121416335009857 | 0.132769043732786 | 0.0180874198520118 | 0.00761152106100782 | 0.0219253543884022 | 0.0137272036308509 | 0.0324286545291913 | 0.0395504865898644 |
| 62 | 0.0961713393892006 | 0.115181168422733 | 0.119007978534169 | 0.114923416004772 | 0.124680411943007 | 0.137444136188212 | 0.0187398935305197 | 0.00763251492772936 | 0.0216603551449294 | 0.0140689519528114 | 0.032204023177553 | 0.0381583674693706 |
| 63 | 0.0982533655606687 | 0.119115853697311 | 0.122832332353825 | 0.118129785892469 | 0.128004879367129 | 0.141839667246952 | 0.0193767949524958 | 0.00762804304136058 | 0.0214084444692689 | 0.0144175918925642 | 0.0319987407242337 | 0.0370718832332204 |
| 64 | 0.100234569460478 | 0.1230096691155 | 0.126548495589718 | 0.121349586958668 | 0.131167313227316 | 0.145998830988331 | 0.0199992802373019 | 0.00760378607459075 | 0.0211874675030234 | 0.0147784477241881 | 0.031787184184955 | 0.0362407812163146 |
| 65 | 0.102066909664753 | 0.126787733556232 | 0.130132644170367 | 0.124517883112714 | 0.133962327807752 | 0.150001888449632 | 0.0206098928425987 | 0.00756678562844133 | 0.0210104143706898 | 0.0151553750503746 | 0.0315351731439246 | 0.0355867250844517 |
| 66 | 0.103707362025903 | 0.130379749751289 | 0.133574193076655 | 0.127571703293984 | 0.136235322876353 | 0.153949169020751 | 0.0212109738807221 | 0.00752488652487079 | 0.0208841382385581 | 0.0155501002904749 | 0.0312066154481807 | 0.0350179640759059 |
| 67 | 0.105126284552729 | 0.13372963701871 | 0.13687883771055 | 0.130458487112494 | 0.137908055841051 | 0.157941244192291 | 0.0218027418861111 | 0.00748604581874544 | 0.0208084147223366 | 0.015961787416333 | 0.0307704994673735 | 0.0344448266219038 |
| 68 | 0.106314359242886 | 0.136803830749128 | 0.140069252056828 | 0.133143325747549 | 0.138991124609595 | 0.162058823904199 | 0.0223812503386378 | 0.00745752393314709 | 0.0207754320590253 | 0.0163868299349337 | 0.0302069593459419 | 0.0337936409187713 |
| 69 | 0.107286676144522 | 0.139596649259928 | 0.14318274577503 | 0.135613695236707 | 0.139580183086526 | 0.166345094240156 | 0.0229365056946735 | 0.00744499319968065 | 0.0207698115473442 | 0.0168188414802352 | 0.0295112375529716 | 0.0330168863330176 |
| 70 | 0.108082796402848 | 0.14213133997332 | 0.146265604336866 | 0.137880627486252 | 0.139835187837575 | 0.170793064554009 | 0.0234510889163424 | 0.00745162655048241 | 0.0207692670292989 | 0.0172488043652946 | 0.0286946767942501 | 0.0320979292681454 |
| 71 | 0.108762118557119 | 0.14445586834016 | 0.149364398181172 | 0.139975704315686 | 0.139944942130052 | 0.175340001875487 | 0.0238996548955231 | 0.00747726184361408 | 0.0207460110845494 | 0.0176653339289289 | 0.0277823693456529 | 0.0310495539714662 |
| 72 | 0.109394542061897 | 0.146633188902892 | 0.152515187041138 | 0.141943860275429 | 0.140082290882921 | 0.179870212860034 | 0.0242496701033795 | 0.00751777096059635 | 0.0206689916276552 | 0.0180550271917607 | 0.0268077228529967 | 0.0299065724258296 |
| 73 | 0.110047210086148 | 0.148726592731335 | 0.155732175130515 | 0.143832682605288 | 0.140358048197252 | 0.184226338537181 | 0.024463672108507 | 0.00756479091218176 | 0.0205069815940007 | 0.0184028844556328 | 0.0258048826286456 | 0.0287139493786083 |
| 74 | 0.110768927004442 | 0.150781681270394 | 0.158997879311931 | 0.145679616938147 | 0.14078355295417 | 0.188228045561775 | 0.0245031799149541 | 0.00760598820356871 | 0.0202324328584029 | 0.0186928167675333 | 0.0248005681142966 | 0.027512927920394 |
| 75 | 0.111574566223384 | 0.152807463108616 | 0.162257127758442 | 0.147499117618404 | 0.141252066844943 | 0.191695662443667 | 0.0243341409599871 | 0.00762601742500928 | 0.0198258339403439 | 0.0189082731192956 | 0.0238073016050991 | 0.0263283776365423 |
| 76 | 0.112432275242004 | 0.154759867172844 | 0.165417080306493 | 0.149272198047707 | 0.141547521199984 | 0.194475093241674 | 0.0239334679092124 | 0.00760828919163376 | 0.0192800786110563 | 0.0190330298191779 | 0.0228200893872462 | 0.0251607925210083 |
| 77 | 0.113256406495219 | 0.156531449263179 | 0.168354838874023 | 0.15094091033634 | 0.141385002224754 | 0.19645945194105 | 0.0232958096902885 | 0.00753756989599173 | 0.0186040733984365 | 0.01905217125922 | 0.0218182303520981 | 0.0239858429080692 |
| 78 | 0.113908727394473 | 0.157951060403096 | 0.17093302325297 | 0.152409896432532 | 0.140480739600175 | 0.19760253045243 | 0.0224392518299211 | 0.00740328647209775 | 0.0178245233723956 | 0.018953248388452 | 0.0207729857284271 | 0.0227630017819819 |
| 79 | 0.114209502076451 | 0.158796567279542 | 0.173021924576448 | 0.153556218741562 | 0.138640582856842 | 0.197919684078242 | 0.0214082166970942 | 0.00720319718926757 | 0.0169846077835453 | 0.0187275246587503 | 0.0196603321513475 | 0.0214525065611947 |
| 80 | 0.113958472135922 | 0.158822209444444 | 0.174524631858509 | 0.154247171253256 | 0.135846025251023 | 0.197473221829569 | 0.0202715409629966 | 0.00694681416011802 | 0.0161382059368808 | 0.0183711124724146 | 0.0184760466099471 | 0.0200369562533247 |
| 81 | 0.112963686616847 | 0.15779973370365 | 0.175399139659574 | 0.154363762408146 | 0.132307691339428 | 0.196342118396035 | 0.0191137047821803 | 0.00665763387692717 | 0.0153386354402193 | 0.0178856813506434 | 0.0172482222322257 | 0.0185406279936999 |
| 82 | 0.111073796736846 | 0.155569031592939 | 0.175669400756413 | 0.15382525808733 | 0.128450984796326 | 0.194579939890395 | 0.0180177019098693 | 0.00637287120790048 | 0.0146217744122066 | 0.0172783143844895 | 0.0160405231835737 | 0.0170369520900909 |
| 83 | 0.108207314572657 | 0.152089710134715 | 0.175416405598305 | 0.152607979886328 | 0.124802016852356 | 0.192170299857321 | 0.01703938406666 | 0.00613904073545934 | 0.0139853106046771 | 0.0165600579933853 | 0.0149389224450731 | 0.0156338402067869 |
| 84 | 0.10437120882081 | 0.147480109040436 | 0.174741863325735 | 0.150750129478582 | 0.121760720035853 | 0.188995768526254 | 0.016175702900725 | 0.0060014521463747 | 0.0133691720497723 | 0.0157428393290703 | 0.0140166273072272 | 0.0144296985818102 |
| 85 | 0.0996622275669411 | 0.142025229657709 | 0.17370263821914 | 0.148334745328937 | 0.119291398376005 | 0.18484354605648 | 0.0153336529428852 | 0.00598557977040175 | 0.0126475767602598 | 0.01483484485213 | 0.0132782325590278 | 0.0134427838975169 |
| 86 | 0.0942491321654843 | 0.136130654114402 | 0.172226090001737 | 0.145446403641876 | 0.116641120583293 | 0.179478669243062 | 0.014313586628578 | 0.00606846574019489 | 0.0116514095033144 | 0.0138353442252423 | 0.0125973840563898 | 0.0125369067198414 |
| 87 | 0.0883448300647948 | 0.130196983205274 | 0.170037962550283 | 0.142105882542147 | 0.112329258118371 | 0.172821942576003 | 0.0128308085292963 | 0.00613900568143288 | 0.0102518306950235 | 0.0127315528614771 | 0.0116857606962933 | 0.0114024469082005 |
| 88 | 0.0821971710887627 | 0.124390264324817 | 0.166669464039449 | 0.138203285932412 | 0.104857446295107 | 0.165273560002143 | 0.0106140576410612 | 0.00594738884033893 | 0.00855344993252751 | 0.0115027820866138 | 0.0101693865907247 | 0.00970982904784027 |
| 89 | 0.0761597678590785 | 0.118290561661802 | 0.161663776882856 | 0.133477410514359 | 0.094893871756109 | 0.158222286329136 | 0.00764000510576825 | 0.00504643636002627 | 0.00726967112384216 | 0.0101412537758645 | 0.00790678744790756 | 0.00764144632466374 |
| 90 | 0.0709545186280278 | 0.110416200560465 | 0.155180818253395 | 0.127631696307115 | 0.0901207764308222 | 0.154771117402492 | 0.00459087706553193 | 0.00273150296908968 | 0.00838592021865174 | 0.00870510006832205 | 0.00576937996876431 | 0.00713810892357435 |

| **Table S11** – Incidence of microalbuminuria and progression from micro- to macroalbuminuria used in S-GASM | | | | | | | | | | | | |
| --- | --- | --- | --- | --- | --- | --- | --- | --- | --- | --- | --- | --- |
| Age | Incidence of microalbuminuria | | | | | | Progression from micro- to macroalbuminuria | | | | | |
|  | men | women | men with hypertension | women with hypertension | men with diabetes | women with diabetes | men | women | men with hypertension | women with hypertension | men with diabetes | women with diabetes |
| 18 | 0 | 0 | 0 | 0 | 0 | 0 | 0 | 0 | 0 | 0 | 0 | 0 |
| 19 | 0 | 0 | 0 | 0 | 0 | 0 | 0 | 0 | 0 | 0 | 0 | 0 |
| 20 | 0 | 0 | 0 | 0 | 0 | 0 | 0 | 0 | 0 | 0 | 0 | 0 |
| 21 | 0 | 0 | 0 | 0 | 0 | 0 | 0 | 0 | 0 | 0 | 0 | 0 |
| 22 | 0 | 0 | 0 | 0 | 0 | 0 | 0 | 0 | 0 | 0 | 0 | 0 |
| 23 | 0 | 0 | 0 | 0 | 0 | 0 | 0 | 0 | 0 | 0 | 0 | 0 |
| 24 | 0 | 0 | 0 | 0 | 0 | 0 | 0 | 0 | 0 | 0 | 0 | 0 |
| 25 | 0 | 0 | 0 | 0 | 0 | 0 | 0 | 0 | 0 | 0 | 0 | 0 |
| 26 | 0 | 0 | 0 | 0 | 0 | 0 | 0 | 0 | 0 | 0 | 0 | 0 |
| 27 | 0 | 0 | 0 | 0 | 0 | 0 | 0 | 0 | 0 | 0 | 0 | 0 |
| 28 | 0 | 0 | 0 | 0 | 0 | 0 | 0 | 0 | 0 | 0 | 0 | 0 |
| 29 | 0 | 0 | 0 | 0 | 0 | 0 | 0 | 0 | 0 | 0 | 0 | 0 |
| 30 | 0 | 0 | 0 | 0 | 0 | 0 | 0 | 0 | 0 | 0 | 0 | 0 |
| 31 | 0.00545 | 0.005459 | 0.006129 | 0.006204 | 0.009705 | 0.008158 | 0.046278 | 0.022149 | 0.056452 | 0.034981 | 0.078391 | 0.037779 |
| 32 | 0.005534 | 0.005534 | 0.006242 | 0.006269 | 0.009735 | 0.008271 | 0.046278 | 0.022149 | 0.056452 | 0.034981 | 0.078391 | 0.037779 |
| 33 | 0.005619 | 0.005609 | 0.006355 | 0.006335 | 0.009765 | 0.008385 | 0.046278 | 0.022149 | 0.056452 | 0.034981 | 0.078391 | 0.037779 |
| 34 | 0.005704 | 0.005684 | 0.006468 | 0.006401 | 0.009796 | 0.008501 | 0.046278 | 0.022149 | 0.056452 | 0.034981 | 0.078391 | 0.037779 |
| 35 | 0.005789 | 0.00576 | 0.006583 | 0.006467 | 0.009827 | 0.008619 | 0.046278 | 0.022149 | 0.056452 | 0.034981 | 0.078391 | 0.037779 |
| 36 | 0.005875 | 0.005835 | 0.006698 | 0.006534 | 0.009858 | 0.008738 | 0.046278 | 0.022149 | 0.056452 | 0.034981 | 0.078391 | 0.037779 |
| 37 | 0.005961 | 0.005912 | 0.006814 | 0.006602 | 0.00989 | 0.008858 | 0.046278 | 0.022149 | 0.056452 | 0.034981 | 0.078391 | 0.037779 |
| 38 | 0.006048 | 0.005988 | 0.006931 | 0.006669 | 0.009922 | 0.00898 | 0.046278 | 0.022149 | 0.056452 | 0.034981 | 0.078391 | 0.037779 |
| 39 | 0.006135 | 0.006065 | 0.007049 | 0.006738 | 0.009955 | 0.009103 | 0.046278 | 0.022149 | 0.056452 | 0.034981 | 0.078391 | 0.037779 |
| 40 | 0.002239 | 0.002159 | 0.003184 | 0.002823 | 0.006004 | 0.005245 | 0.00595 | 0.0024 | 0.028699 | 0.007252 | 0.017079 | 0.037976 |
| 41 | 0.002327 | 0.002237 | 0.003304 | 0.002892 | 0.006037 | 0.005372 | 0.00595 | 0.0024 | 0.028699 | 0.007252 | 0.017079 | 0.037976 |
| 42 | 0.002416 | 0.002315 | 0.003424 | 0.002962 | 0.006071 | 0.005501 | 0.00595 | 0.0024 | 0.028699 | 0.007252 | 0.017079 | 0.037976 |
| 43 | 0.002505 | 0.002394 | 0.003546 | 0.003032 | 0.006105 | 0.005631 | 0.00595 | 0.0024 | 0.028699 | 0.007252 | 0.019538 | 0.037976 |
| 44 | 0.002595 | 0.002473 | 0.00367 | 0.003103 | 0.006139 | 0.005764 | 0.00595 | 0.0024 | 0.028699 | 0.007252 | 0.019538 | 0.037976 |
| 45 | 0.002685 | 0.002553 | 0.003794 | 0.003174 | 0.006174 | 0.005899 | 0.00595 | 0.0024 | 0.028699 | 0.007252 | 0.019538 | 0.037976 |
| 46 | 0.002776 | 0.002633 | 0.003919 | 0.003247 | 0.006209 | 0.006036 | 0.00595 | 0.0024 | 0.028699 | 0.007252 | 0.019538 | 0.037976 |
| 47 | 0.002868 | 0.002714 | 0.004046 | 0.003319 | 0.006245 | 0.006174 | 0.00595 | 0.0024 | 0.028699 | 0.007252 | 0.019538 | 0.037976 |
| 48 | 0.00296 | 0.002795 | 0.004175 | 0.003393 | 0.006281 | 0.006316 | 0.00595 | 0.0024 | 0.028699 | 0.007252 | 0.019538 | 0.037976 |
| 49 | 0.003053 | 0.002877 | 0.004304 | 0.003466 | 0.006318 | 0.006459 | 0.00595 | 0.0024 | 0.028699 | 0.007252 | 0.019538 | 0.037976 |
| 50 | 0.003147 | 0.00296 | 0.004436 | 0.003541 | 0.006355 | 0.006605 | 0.00595 | 0.0024 | 0.028699 | 0.007252 | 0.019538 | 0.037976 |
| 51 | 0.003242 | 0.003043 | 0.004568 | 0.003616 | 0.006392 | 0.006754 | 0.00595 | 0.0024 | 0.028699 | 0.007252 | 0.019538 | 0.037976 |
| 52 | 0.003337 | 0.003127 | 0.004703 | 0.003693 | 0.00643 | 0.006905 | 0.00595 | 0.0024 | 0.028699 | 0.007252 | 0.019538 | 0.037976 |
| 53 | 0.003433 | 0.003212 | 0.004839 | 0.003769 | 0.006468 | 0.007059 | 0.00595 | 0.0024 | 0.028699 | 0.007252 | 0.019538 | 0.037976 |
| 54 | 0.003531 | 0.003297 | 0.004977 | 0.003847 | 0.006507 | 0.007216 | 0.00595 | 0.0024 | 0.028699 | 0.007252 | 0.019538 | 0.037976 |
| 55 | 0.003629 | 0.003383 | 0.005117 | 0.003925 | 0.006547 | 0.007375 | 0.00595 | 0.0024 | 0.028699 | 0.007252 | 0.019538 | 0.037976 |
| 56 | 0.004326 | 0.004069 | 0.005857 | 0.004603 | 0.007185 | 0.008137 | 0.012844 | 0.001737 | 0.006563 | 0.009467 | 0.019686 | 0.010385 |
| 57 | 0.004426 | 0.004156 | 0.006001 | 0.004683 | 0.007225 | 0.008303 | 0.012844 | 0.001737 | 0.006563 | 0.009467 | 0.019686 | 0.010385 |
| 58 | 0.004527 | 0.004245 | 0.006147 | 0.004764 | 0.007266 | 0.008472 | 0.012844 | 0.001737 | 0.006563 | 0.009467 | 0.014297 | 0.010385 |
| 59 | 0.00463 | 0.004334 | 0.006295 | 0.004846 | 0.007307 | 0.008645 | 0.012844 | 0.001737 | 0.006563 | 0.009467 | 0.014297 | 0.010385 |
| 60 | 0.004733 | 0.004425 | 0.006445 | 0.004928 | 0.007349 | 0.008821 | 0.012844 | 0.001737 | 0.006563 | 0.009467 | 0.014297 | 0.010385 |
| 61 | 0.004837 | 0.004516 | 0.006597 | 0.005012 | 0.007392 | 0.009001 | 0.012844 | 0.001737 | 0.006563 | 0.009467 | 0.014297 | 0.010385 |
| 62 | 0.004943 | 0.004608 | 0.006752 | 0.005097 | 0.007435 | 0.009185 | 0.012844 | 0.001737 | 0.006563 | 0.009467 | 0.014297 | 0.010385 |
| 63 | 0.00505 | 0.004701 | 0.00691 | 0.005182 | 0.007479 | 0.009373 | 0.012844 | 0.001737 | 0.006563 | 0.009467 | 0.014297 | 0.010385 |
| 64 | 0.005158 | 0.004795 | 0.00707 | 0.005269 | 0.007523 | 0.009565 | 0.012844 | 0.001737 | 0.006563 | 0.009467 | 0.014297 | 0.010385 |
| 65 | 0.005267 | 0.00489 | 0.007233 | 0.005357 | 0.007568 | 0.009762 | 0.012844 | 0.001737 | 0.006563 | 0.009467 | 0.014297 | 0.010385 |
| 66 | 0.005378 | 0.004987 | 0.007399 | 0.005445 | 0.007613 | 0.009963 | 0.012844 | 0.001737 | 0.006563 | 0.009467 | 0.014297 | 0.010385 |
| 67 | 0.00549 | 0.005084 | 0.007568 | 0.005535 | 0.007659 | 0.010169 | 0.012844 | 0.001737 | 0.006563 | 0.009467 | 0.014297 | 0.010385 |
| 68 | 0.005603 | 0.005183 | 0.00774 | 0.005626 | 0.007706 | 0.01038 | 0.012844 | 0.001737 | 0.006563 | 0.009467 | 0.014297 | 0.010385 |
| 69 | 0.005719 | 0.005283 | 0.007915 | 0.005719 | 0.007753 | 0.010596 | 0.012844 | 0.001737 | 0.006563 | 0.009467 | 0.014297 | 0.010385 |
| 70 | 0.005835 | 0.005384 | 0.008093 | 0.005812 | 0.007801 | 0.010818 | 0.012844 | 0.001737 | 0.006563 | 0.009467 | 0.014297 | 0.010385 |
| 71 | 0.005954 | 0.005486 | 0.008275 | 0.005907 | 0.007849 | 0.011046 | 0.012844 | 0.001737 | 0.006563 | 0.009467 | 0.014297 | 0.010385 |
| 72 | 0.006074 | 0.005589 | 0.008461 | 0.006003 | 0.007899 | 0.011279 | 0.012844 | 0.001737 | 0.006563 | 0.009467 | 0.014297 | 0.010385 |
| 73 | 0.006195 | 0.005694 | 0.008651 | 0.006101 | 0.007949 | 0.011519 | 0.012844 | 0.001737 | 0.006563 | 0.009467 | 0.014297 | 0.010385 |
| 74 | 0.006319 | 0.005801 | 0.008844 | 0.006199 | 0.007999 | 0.011765 | 0.012844 | 0.001737 | 0.006563 | 0.009467 | 0.014297 | 0.010385 |
| 75 | 0.006444 | 0.005909 | 0.009042 | 0.0063 | 0.008051 | 0.012018 | 0.012844 | 0.001737 | 0.006563 | 0.009467 | 0.014297 | 0.010385 |
| 76 | 0.006572 | 0.006018 | 0.009244 | 0.006402 | 0.008103 | 0.012278 | 0.012844 | 0.001737 | 0.006563 | 0.009467 | 0.014297 | 0.010385 |
| 77 | 0.006701 | 0.006129 | 0.00945 | 0.006505 | 0.008156 | 0.012545 | 0.012844 | 0.001737 | 0.006563 | 0.009467 | 0.014297 | 0.010385 |
| 78 | 0.006832 | 0.006242 | 0.009662 | 0.00661 | 0.00821 | 0.012821 | 0.012844 | 0.001737 | 0.006563 | 0.009467 | 0.014297 | 0.010385 |
| 79 | 0.006966 | 0.006356 | 0.009878 | 0.006716 | 0.008264 | 0.013105 | 0.012844 | 0.001737 | 0.006563 | 0.009467 | 0.014297 | 0.010385 |
| 80 | 0.007102 | 0.006472 | 0.010099 | 0.006825 | 0.00832 | 0.013398 | 0.012844 | 0.001737 | 0.006563 | 0.009467 | 0.014297 | 0.010385 |
| 81 | 0.00724 | 0.006589 | 0.010326 | 0.006934 | 0.008376 | 0.013699 | 0.012844 | 0.001737 | 0.006563 | 0.009467 | 0.014297 | 0.010385 |
| 82 | 0.00738 | 0.006709 | 0.010559 | 0.007046 | 0.008433 | 0.014011 | 0.012844 | 0.001737 | 0.006563 | 0.009467 | 0.014297 | 0.010385 |
| 83 | 0.007523 | 0.00683 | 0.010797 | 0.00716 | 0.008491 | 0.014332 | 0.012844 | 0.001737 | 0.006563 | 0.009467 | 0.014297 | 0.010385 |
| 84 | 0.007669 | 0.006954 | 0.011041 | 0.007275 | 0.008549 | 0.014665 | 0.012844 | 0.001737 | 0.006563 | 0.009467 | 0.014297 | 0.010385 |
| 85 | 0.007817 | 0.007079 | 0.011292 | 0.007393 | 0.008609 | 0.015008 | 0.012844 | 0.001737 | 0.006563 | 0.009467 | 0.014297 | 0.010385 |
| 86 | 0.007968 | 0.007207 | 0.01155 | 0.007512 | 0.00867 | 0.015364 | 0.012844 | 0.001737 | 0.006563 | 0.009467 | 0.014297 | 0.010385 |
| 87 | 0.008122 | 0.007337 | 0.011815 | 0.007634 | 0.008731 | 0.015733 | 0.012844 | 0.001737 | 0.006563 | 0.009467 | 0.014297 | 0.010385 |
| 88 | 0.008279 | 0.007469 | 0.012088 | 0.007758 | 0.008794 | 0.016114 | 0.012844 | 0.001737 | 0.006563 | 0.009467 | 0.014297 | 0.010385 |
| 89 | 0.008439 | 0.007603 | 0.012368 | 0.007884 | 0.008858 | 0.01651 | 0.012844 | 0.001737 | 0.006563 | 0.009467 | 0.014297 | 0.010385 |
| 90 | 0.008602 | 0.00774 | 0.012657 | 0.008012 | 0.008922 | 0.016921 | 0.012844 | 0.001737 | 0.006563 | 0.009467 | 0.014297 | 0.010385 |

| **Table S12** – Values for assigning GFR used in S-GASM | | | | | | | | | | | | | | | | | | |
| --- | --- | --- | --- | --- | --- | --- | --- | --- | --- | --- | --- | --- | --- | --- | --- | --- | --- | --- |
| **Age** | **No albuminuria** | | | | | | **Microalbuminuria** | | | | | | **Macroalbuminuria** | | | | | |
|  | **No Diabetes, no hypertension** | | **No Diabetes, with hypertension** | | **With Diabetes** | | **No Diabetes, no hypertension** | | **No Diabetes, with hypertension** | | **With Diabetes** | | **No Diabetes, no hypertension** | | **No Diabetes, with hypertension** | | **With Diabetes** | |
|  | **Mean** | **Standard Deviation** | **Mean** | **Standard Deviation** | **Mean** | **Standard Deviation** | **Mean** | **Standard Deviation** | **Mean** | **Standard Deviation** | **Mean** | **Standard Deviation** | **Mean** | **Standard Deviation** | **Mean** | **Standard Deviation** | **Mean** | **Standard Deviation** |
| 18 | 101.9409 | 19.313 | 101.9409 | 19.313 | 101.9409 | 19.313 | 101.9409 | 19.313 | 101.9409 | 19.313 | 101.9409 | 19.313 | 101.9409 | 19.313 | 101.9409 | 19.313 | 101.9409 | 19.313 |
| 19 | 101.9409 | 19.313 | 101.9409 | 19.313 | 101.9409 | 19.313 | 101.9409 | 19.313 | 101.9409 | 19.313 | 101.9409 | 19.313 | 101.9409 | 19.313 | 101.9409 | 19.313 | 101.9409 | 19.313 |
| 20 | 101.9409 | 19.313 | 101.9409 | 19.313 | 101.9409 | 19.313 | 101.9409 | 19.313 | 101.9409 | 19.313 | 101.9409 | 19.313 | 101.9409 | 19.313 | 101.9409 | 19.313 | 101.9409 | 19.313 |
| 21 | 101.9409 | 19.313 | 101.9409 | 19.313 | 101.9409 | 19.313 | 101.9409 | 19.313 | 101.9409 | 19.313 | 101.9409 | 19.313 | 101.9409 | 19.313 | 101.9409 | 19.313 | 101.9409 | 19.313 |
| 22 | 101.9409 | 19.313 | 101.9409 | 19.313 | 101.9409 | 19.313 | 101.9409 | 19.313 | 101.9409 | 19.313 | 101.9409 | 19.313 | 101.9409 | 19.313 | 101.9409 | 19.313 | 101.9409 | 19.313 |
| 23 | 101.9409 | 19.313 | 101.9409 | 19.313 | 101.9409 | 19.313 | 101.9409 | 19.313 | 101.9409 | 19.313 | 101.9409 | 19.313 | 101.9409 | 19.313 | 101.9409 | 19.313 | 101.9409 | 19.313 |
| 24 | 101.9409 | 19.313 | 101.9409 | 19.313 | 101.9409 | 19.313 | 101.9409 | 19.313 | 101.9409 | 19.313 | 101.9409 | 19.313 | 101.9409 | 19.313 | 101.9409 | 19.313 | 101.9409 | 19.313 |
| 25 | 101.9409 | 19.313 | 101.9409 | 19.313 | 101.9409 | 19.313 | 101.9409 | 19.313 | 101.9409 | 19.313 | 101.9409 | 19.313 | 101.9409 | 19.313 | 101.9409 | 19.313 | 101.9409 | 19.313 |
| 26 | 101.9409 | 19.313 | 101.9409 | 19.313 | 101.9409 | 19.313 | 101.9409 | 19.313 | 101.9409 | 19.313 | 101.9409 | 19.313 | 101.9409 | 19.313 | 101.9409 | 19.313 | 101.9409 | 19.313 |
| 27 | 101.9409 | 19.313 | 101.9409 | 19.313 | 101.9409 | 19.313 | 101.9409 | 19.313 | 101.9409 | 19.313 | 101.9409 | 19.313 | 101.9409 | 19.313 | 101.9409 | 19.313 | 101.9409 | 19.313 |
| 28 | 101.9409 | 19.313 | 101.9409 | 19.313 | 101.9409 | 19.313 | 101.9409 | 19.313 | 101.9409 | 19.313 | 101.9409 | 19.313 | 101.9409 | 19.313 | 101.9409 | 19.313 | 101.9409 | 19.313 |
| 29 | 101.9409 | 19.313 | 101.9409 | 19.313 | 101.9409 | 19.313 | 101.9409 | 19.313 | 101.9409 | 19.313 | 101.9409 | 19.313 | 101.9409 | 19.313 | 101.9409 | 19.313 | 101.9409 | 19.313 |
| 30 | 101.9409 | 19.313 | 101.9409 | 19.313 | 101.9409 | 19.313 | 101.9409 | 19.313 | 101.9409 | 19.313 | 101.9409 | 19.313 | 101.9409 | 19.313 | 101.9409 | 19.313 | 101.9409 | 19.313 |
| 31 | 101.234739190161 | 19.2904954093282 | 101.225813585924 | 19.4297369034472 | 100.824826613691 | 18.9324693022665 | 100.771205262803 | 19.6603765031927 | 101.464818268867 | 19.6195927138787 | 97.2129619987276 | 21.4434656574862 | 100.591559682788 | 19.1604552200402 | 100.6025322022 | 19.1611389887226 | 100.6025322022 | 19.1611389887226 |
| 32 | 100.890884248229 | 19.2923578715294 | 100.880128217145 | 19.4613819271583 | 100.236283463057 | 18.9784997285104 | 100.299153188791 | 19.7696101885489 | 101.150714489209 | 19.6945774198815 | 95.5327811775642 | 21.9670070486375 | 100.019371905043 | 19.1639193653278 | 100.10146627557 | 19.3837333409161 | 100.10146627557 | 19.3837333409161 |
| 33 | 100.50477608315 | 19.2954783164649 | 100.497810685605 | 19.493694048992 | 99.5854794728901 | 19.0495194748815 | 99.7867744918176 | 19.8854137555446 | 100.782416469229 | 19.7709663528077 | 93.736546377208 | 22.4978017648376 | 99.4219968242339 | 19.1725053611012 | 99.5636447628988 | 19.6426794995084 | 99.5636447628988 | 19.6426794995084 |
| 34 | 100.082937919007 | 19.2999056699227 | 100.086259560114 | 19.5261967334921 | 98.8873509665441 | 19.1436517104679 | 99.244100521339 | 20.0062802161357 | 100.364632158359 | 19.8487607275411 | 91.8644707326617 | 23.0263926241878 | 98.8133628762636 | 19.1892976779521 | 98.9992993898731 | 19.9293202797265 | 98.9992993898731 | 19.9293202797265 |
| 35 | 99.6318238473715 | 19.305726335475 | 99.6523303902335 | 19.5586174231406 | 98.1561729067593 | 19.2586549566354 | 98.6801192349267 | 20.1312345172052 | 99.9027712208589 | 19.9284677510777 | 89.9540817991312 | 23.5454418060756 | 98.2037770090071 | 19.2182057165349 | 98.4172031532089 | 20.2353382160437 | 98.4172031532089 | 20.2353382160437 |
| 36 | 99.1575791359991 | 19.3130617088118 | 99.2021161801403 | 19.5908791069216 | 97.4050644915957 | 19.3920811998963 | 98.1025370551865 | 20.2598073765022 | 99.4026747962615 | 20.010996646362 | 88.0392294296654 | 24.0496861330697 | 97.5998982995022 | 19.2636685952377 | 97.8245221591636 | 20.5530589401417 | 97.8245221591636 | 20.5530589401417 |
| 37 | 98.6658669888899 | 19.3220621345546 | 98.7408180979833 | 19.6230811883145 | 96.64565122478 | 19.5414049584066 | 97.5176638715059 | 20.3919746316158 | 98.870378842165 | 20.0975391804189 | 86.1494608333575 | 24.5357781152767 | 97.0049645901246 | 19.3303313521115 | 97.2268024017966 | 20.8756461280091 | 97.2268024017966 | 20.8756461280091 |
| 38 | 98.1617518695762 | 19.33289845252 | 98.2726897046462 | 19.6554728260471 | 95.8878589112733 | 19.70412629857 | 96.9303962300364 | 20.5280731317378 | 98.3119098799673 | 20.1894442962569 | 84.3096980259904 | 25.0020458181267 | 96.4192031166636 | 19.4227160579652 | 96.6280577767197 | 21.1972081868729 | 96.6280577767197 | 21.1972081868729 |
| 39 | 97.6496303522394 | 19.3457521249143 | 97.8010396901652 | 19.6884215136055 | 95.1398172098554 | 19.8778506513311 | 96.34427646532 | 20.6687027044305 | 97.7331118338299 | 20.2880948540267 | 82.5401594407621 | 25.4482003428337 | 95.8403647930174 | 19.5449073614908 | 96.0309299034416 | 21.5128347095459 | 96.0309299034416 | 21.5128347095459 |
| 40 | 97.1332012840211 | 19.3608047888979 | 97.3282797324741 | 19.722379277389 | 94.4078524673423 | 20.0603481313421 | 95.7616081070142 | 20.8146223143494 | 97.1395025342549 | 20.3947929919048 | 80.8564737173073 | 25.8750156653313 | 95.2643300514791 | 19.7002682560232 | 95.4368943736981 | 21.8185786827228 | 95.4368943736981 | 21.8185786827228 |
| 41 | 96.6154678267213 | 19.3782279403518 | 96.8560056416549 | 19.757848508505 | 93.6965515636817 | 20.2495949162877 | 95.1836103452669 | 20.9666471978638 | 96.5361583502086 | 20.5106592211347 | 79.2699396326294 | 26.2840017643019 | 94.6857419142956 | 19.8911983710599 | 94.8464918543618 | 22.1113984967919 | 94.8464918543618 | 22.1113984967919 |
| 42 | 96.0987646969701 | 19.3981733245251 | 96.3851014234804 | 19.7953490995288 | 93.0088804136354 | 20.4437990982517 | 94.6105966584344 | 21.1255525067804 | 95.9276253176998 | 20.6365490726063 | 77.7878917777811 | 26.6770883820085 | 94.098629291335 | 20.1189438499758 | 94.2595660516588 | 22.389071967832 | 94.2595660516588 | 22.389071967832 |
| 43 | 95.5848046409029 | 19.4207644875198 | 95.9158572887214 | 19.8353882350987 | 92.3463425990305 | 20.6414132741054 | 94.0421648970232 | 21.2919878259052 | 95.3178550481381 | 20.7729899142723 | 76.4141369191736 | 27.0563334029944 | 93.4969903603164 | 20.3834648784591 | 93.675493888466 | 22.6500928476489 | 93.675493888466 | 22.6500928476489 |
| 44 | 95.0747388623722 | 19.4460898292978 | 95.4480939494789 | 19.8784338839843 | 91.7091653456602 | 20.8411359974958 | 93.4773881779698 | 21.466405842967 | 94.7101636266417 | 20.9201394596093 | 75.1494310132611 | 27.4236667053442 | 92.8753122901174 | 20.6833641760738 | 93.0933963584407 | 22.8935586649567 | 93.0933963584407 | 22.8935586649567 |
| 45 | 94.5692267727981 | 19.4741973940622 | 94.9812867806727 | 19.9248927602541 | 91.0965017108365 | 21.0419040711014 | 92.9149978736554 | 21.6490074454921 | 94.1072116487494 | 21.0777664872057 | 73.9919715680343 | 27.7806774370938 | 92.2290095141981 | 21.0158772560129 | 92.5123213996237 | 23.1190572101451 | 92.5123213996237 | 23.1190572101451 |
| 46 | 94.0685110458735 | 19.5050915374746 | 94.5146845837841 | 19.9750932623699 | 90.5066394113355 | 21.2428775144205 | 92.3535517803786 | 21.8397045975361 | 93.5110034937082 | 21.2452533904262 | 72.937884464266 | 28.1284499965414 | 91.5547682501462 | 21.3769229955544 | 91.931392775977 | 23.3265575474773 | 91.931392775977 | 23.3265575474773 |
| 47 | 93.5724945415248 | 19.5387315222258 | 94.0474187710015 | 20.0292736614659 | 89.9372081970377 | 21.443418899444 | 91.7915822213834 | 22.0381015097779 | 92.9229038936594 | 21.4216193739365 | 71.9816884637829 | 28.4674515496463 | 90.8507899927801 | 21.7612110419623 | 91.3499213680569 | 23.5163101120457 | 91.3499213680569 | 23.5163101120457 |
| 48 | 93.0808162107655 | 19.5750320139689 | 93.578599791068 | 20.0875755937097 | 89.3853790629487 | 21.6430686041558 | 91.2277203799585 | 22.2434938593302 | 92.3436698306347 | 21.6055624106965 | 71.1167244411921 | 28.797471700401 | 90.1169312821169 | 22.1624008055904 | 90.7674774536814 | 23.68875922437 | 90.7674774536814 | 23.68875922437 |
| 49 | 92.5929236063745 | 19.6138653785651 | 93.1073985433614 | 20.1500427165119 | 88.8480498914862 | 21.8415173918901 | 90.6607945685948 | 22.4548851407471 | 91.7734957772941 | 21.795517468824 | 70.335539879463 | 29.1176129410357 | 89.3547411638515 | 22.5733052637445 | 90.1839245060348 | 23.8444702331524 | 90.1839245060348 | 23.8444702331524 |
| 50 | 92.108140103687 | 19.6550656189728 | 92.6331113740666 | 20.216624214429 | 88.3220133279481 | 22.0385775831623 | 90.0899014206941 | 22.6710186370963 | 91.2120702927943 | 21.9897280125107 | 69.6302223685652 | 29.4263287481525 | 88.5674014187689 | 22.9861315154454 | 89.5994167501601 | 23.9840724764141 | 89.5994167501601 | 23.9840724764141 |
| 51 | 91.6257253811974 | 19.6984337359315 | 92.1552080177157 | 20.2871826879113 | 87.8041038149149 | 22.2341539456728 | 89.51445014189 | 22.8904229896157 | 90.6586419920679 | 22.1863273749291 | 68.9926787399798 | 29.7215046583952 | 87.7595778387564 | 23.3927489886025 | 89.0143641992113 | 24.1082183330176 | 89.0143641992113 | 24.1082183330176 |
| 52 | 91.1449281221455 | 19.7437442508597 | 91.6733625388798 | 20.3615058265674 | 87.2913217460112 | 22.4282152877673 | 88.9341809786402 | 23.1114689163965 | 90.1120929251197 | 22.3834262938024 | 68.4148590583424 | 30.0005763530363 | 86.9371935707671 | 23.7849754072605 | 88.4293701391919 | 24.2175578204379 | 88.4293701391919 | 24.2175578204379 |
| 53 | 90.6650302757846 | 19.790752592094 | 91.1874679423931 | 20.4393211583607 | 86.7809336459346 | 22.6207676007165 | 88.3491599524072 | 23.3324342847158 | 89.5710174327062 | 22.5792026920279 | 67.8889269747389 | 30.2606777049123 | 86.1071378362319 | 24.1548700775206 | 87.8451470451744 | 24.3127274805789 | 87.8451470451744 | 24.3127274805789 |
| 54 | 90.1853825606194 | 19.8392030167465 | 90.6976356561813 | 20.5203130771121 | 86.2705481419679 | 22.8118294557589 | 87.7597526684412 | 23.5515744780974 | 89.0338045859609 | 22.7719896764345 | 67.4073799242599 | 30.4988108934487 | 85.2769251620137 | 24.4950237464592 | 87.2624176931877 | 24.3943516834371 | 87.2624176931877 | 24.3943516834371 |
| 55 | 89.7054312015447 | 19.8888367200511 | 90.2041815485429 | 20.6041412828653 | 85.7581682623219 | 23.0014102229277 | 87.1665806389235 | 23.7671948178924 | 88.4987223701584 | 22.9603577174315 | 66.9631243233246 | 30.7120300741033 | 84.4543216300458 | 24.7988342288751 | 86.6818087800779 | 24.4630539685003 | 86.6818087800779 | 24.4630539685003 |
| 56 | 89.2247361685234 | 19.9393997721054 | 89.707600521603 | 20.6904597230709 | 85.2422212786013 | 23.1894915402592 | 86.5704640610229 | 23.9777217001558 | 87.9640018368745 | 23.1431870609559 | 66.5495122900131 | 30.8976296974059 | 83.6469555662986 | 25.0607571819851 | 86.1037446786799 | 24.5194756359264 | 86.1037446786799 | 24.5194756359264 |
| 57 | 88.7429814261988 | 19.9906505183925 | 89.2085320246205 | 20.778935097165 | 84.7215679034605 | 23.3760123240534 | 85.9723553612537 | 24.181769091843 | 87.4279195242994 | 23.3197266117586 | 66.1603474731989 | 31.0533284098961 | 82.8619305456769 | 25.276521838267 | 85.5283490376021 | 24.5642984937793 | 85.5283490376021 | 24.5642984937793 |
| 58 | 88.2599779116631 | 20.0423660853885 | 88.7077190548791 | 20.8692639829566 | 84.1954931601044 | 23.5608574734331 | 85.3732680584094 | 24.3781970958629 | 86.8888765323959 | 23.4896358136793 | 65.7898683336401 | 31.1774395346499 | 82.105458587854 | 25.4433021815067 | 84.9553617838025 | 24.5982684639102 | 84.9553617838025 | 24.5982684639102 |
| 59 | 87.7756601314757 | 20.0943476459248 | 88.2059633600353 | 20.9611876602958 | 83.6636816577043 | 23.7438502855159 | 84.7742056082772 | 24.5661594423066 | 86.3454727379648 | 23.6530064381504 | 65.4327176723869 | 31.2690194227437 | 81.3825309599159 | 25.5598349707532 | 84.3840787019404 | 24.6222166474548 | 84.3840787019404 | 24.6222166474548 |
| 60 | 87.2900774089681 | 20.1464241207781 | 87.7040796240281 | 21.0545037457753 | 83.1261803360296 | 23.9247484610862 | 84.1760948743168 | 24.7451369932137 | 85.7965747444808 | 23.8103616767396 | 65.0839073488744 | 31.3279854889326 | 80.6966430849979 | 25.626477181313 | 83.813320147213 | 24.6370744513762 | 83.813320147213 | 24.6370744513762 |
| 61 | 86.8033809188617 | 20.1984540232139 | 87.202851408978 | 21.1490738117109 | 82.5833519836507 | 24.1032434447201 | 83.5797287195091 | 24.9149546605511 | 85.2413762828046 | 23.9626305170872 | 64.7387869729088 | 31.3551964950178 | 80.0495886818682 | 25.6451968411311 | 83.2414345980313 | 24.6438794800179 | 83.2414345980313 | 24.6438794800179 |
| 62 | 86.3158077182821 | 20.250325191894 | 86.702991537917 | 21.2448262433666 | 82.0358229869451 | 24.2789627078837 | 82.9857219356525 | 25.0757795316547 | 84.6794499115474 | 24.111096064125 | 64.3930248914167 | 31.3524886228408 | 79.4413364286318 | 25.6194928949027 | 82.6663416714621 | 24.6437691002394 | 82.6663416714621 | 24.6437691002394 |
| 63 | 85.8276630213626 | 20.3019522046978 | 86.2051094376931 | 21.3417536903248 | 81.4844288318325 | 24.4514744485906 | 82.3944843175014 | 25.2280984732301 | 84.1107890099742 | 24.257317249965 | 64.0426090223022 | 31.3226620855768 | 78.8700001544004 | 25.5542416270448 | 82.0856179078503 | 24.6379588953955 | 82.0856179078503 | 24.6379588953955 |
| 64 | 85.3393019688002 | 20.3532713215788 | 85.7096877179905 | 21.4399045910574 | 80.9301608566827 | 24.6202940467657 | 81.8062141503036 | 25.3726740441129 | 83.5358392118747 | 24.4030242563378 | 63.6838740140677 | 31.2694144610036 | 78.3319108159052 | 25.455469318222 | 81.4966270814531 | 24.6277036321727 | 81.4966270814531 | 24.6277036321727 |
| 65 | 84.8511121139558 | 20.4042328685991 | 85.2170699410884 | 21.5393683941282 | 80.3741176431764 | 24.7848914805272 | 81.2209147105048 | 25.5104781883669 | 82.9555185958083 | 24.5499889529193 | 63.3135598309722 | 31.1972185936933 | 77.821795311603 | 25.3300531984755 | 80.8966950112483 | 24.6142388751408 | 80.8966950112483 | 24.6142388751408 |
| 66 | 84.3634977833757 | 20.4547910477425 | 84.727461137759 | 21.6402542650535 | 79.8174642320597 | 24.9446987751546 | 80.6384355806417 | 25.6426039039375 | 82.3712261255417 | 24.6998727333337 | 62.9289051794518 | 31.1111448046137 | 77.3330635238663 | 25.1853553931397 | 80.2833268303403 | 24.5987019967784 | 80.2833268303403 | 24.5987019967784 |
| 67 | 83.8768673719558 | 20.5048912380147 | 84.2409421475712 | 21.7426632546584 | 79.2614020627115 | 25.0991164235733 | 80.0585396507532 | 25.7701558879835 | 81.7848380243469 | 24.854055308039 | 62.5277772022944 | 31.0166292664373 | 76.8582008603273 | 25.0287974356521 | 79.6544624235645 | 24.5820320457242 | 79.6544624235645 | 24.5820320457242 |
| 68 | 83.3916245013753 | 20.5544549426797 | 83.7574983068226 | 21.8466541128021 | 78.7071521592424 | 25.2475175847396 | 79.4809956199817 | 25.8941220481804 | 81.1986919681071 | 25.0134492897088 | 62.1088365726517 | 30.9191927509327 | 76.3892589883898 | 24.8673860440671 | 79.0087632609915 | 24.564848753061 | 79.0087632609915 | 24.564848753061 |
| 69 | 82.9081638048827 | 20.6033626352749 | 83.27706237437 | 21.9522031605977 | 78.1559536214678 | 25.3892487343667 | 78.9056946234372 | 26.0152287397304 | 80.6155591948959 | 25.178306782099 | 61.6717345203833 | 30.8241165311604 | 75.9184324223116 | 24.7072042255777 | 78.3459211400587 | 24.5473118755783 | 78.3459211400587 | 24.5473118755783 |
| 70 | 82.4268719020414 | 20.6514348632757 | 82.799570874767 | 22.0591588847219 | 77.609078926536 | 25.5236253109868 | 78.3327882908376 | 26.1337836405114 | 80.0386048528466 | 25.3480256577555 | 61.2173354184628 | 30.7360850258103 | 75.4387031301082 | 24.5528853856193 | 77.6669764009978 | 24.528963096169 | 77.6669764009978 | 24.528963096169 |
| 71 | 81.9481338936164 | 20.6984120829535 | 82.3250322493452 | 22.1671921891058 | 77.0678669062607 | 25.6499207693926 | 77.7628440949243 | 26.2495113117715 | 79.4713371437156 | 25.5209647832543 | 60.747955347232 | 30.6588078059049 | 74.9445293778237 | 24.4070919751945 | 76.9746299990986 | 24.5085538248025 | 76.9746299990986 | 24.5085538248025 |
| 72 | 81.4723454394201 | 20.7439338220905 | 81.8536043391942 | 22.275743532205 | 76.5337735362865 | 25.7673473230579 | 77.1970122691924 | 26.3613877090062 | 78.9175460665633 | 25.694279122984 | 60.2676035391663 | 30.5946368452414 | 74.4325486214691 | 24.2700243118951 | 76.2735304031361 | 24.4838634688766 | 76.2735304031361 | 24.4838634688766 |
| 73 | 80.9999301806294 | 20.7875178987689 | 81.3856777794068 | 22.383968492179 | 76.0084398561238 | 25.8750265271782 | 76.6371968660503 | 26.4674812071595 | 78.3812328244325 | 25.863787423783 | 59.782209786516 | 30.5441983853725 | 73.9022573921296 | 24.1389895567337 | 75.5705126419995 | 24.4515140691886 | 75.5705126419995 | 24.4515140691886 |
| 74 | 80.5313619328397 | 20.8285405644556 | 80.9219608614538 | 22.490683637661 | 75.4937754338138 | 25.9719477245271 | 76.0862216881572 | 26.5648090860534 | 77.8665312267959 | 26.0238870530159 | 59.299816768709 | 30.5060625036144 | 73.3566237974029 | 24.0080654203032 | 74.8747629411955 | 24.4067886272776 | 74.8747629411955 | 24.4067886272776 |
| 75 | 80.0671907069279 | 20.8662185880484 | 80.4635603201453 | 22.5943149383663 | 74.9920547955334 | 26.0569122473623 | 75.5479798573549 | 26.6492198859835 | 77.3776227018676 | 26.1675325319334 | 58.8307118257436 | 30.4764774185289 | 72.8025804824377 | 23.8678980079843 | 74.1978782774544 | 24.3434619814742 | 74.1978782774544 | 24.3434619814742 |
| 76 | 79.608071211671 | 20.8995944544321 | 80.0120513233162 | 22.692851327578 | 74.5060231588149 | 26.1284611401653 | 75.0275536873375 | 26.7153135897169 | 76.9186468256356 | 26.286296374393 | 58.3874679679679 | 30.4491997415596 | 72.2513376564081 | 23.7056782949012 | 73.5537858331443 | 24.2536547226611 | 73.5537858331443 | 24.2536547226611 |
| 77 | 79.1547920549891 | 20.9275260164252 | 79.5695286861444 | 22.7838064275557 | 74.0390066382463 | 26.1847840400369 | 74.5312892979643 | 26.7564132156907 | 76.4936095786656 | 26.3705330082361 | 57.9848588716263 | 30.4154562859947 | 71.7184470952741 | 23.5053470470941 | 72.9584827525969 | 24.1277223764793 | 72.9584827525969 | 24.1277223764793 |
| 78 | 78.7083033896683 | 20.9486821127679 | 79.1386299978754 | 22.864191869141 | 73.5950208345251 | 26.2236067241118 | 74.0668080509384 | 26.7646031180397 | 76.1062918573564 | 26.4096688228056 | 57.6396072633671 | 30.3640776751974 | 71.2235388791485 | 23.2480835759247 | 72.4295517897718 | 23.954193916532 | 72.4295517897718 | 23.954193916532 |
| 79 | 78.2697402444644 | 20.9615458470136 | 78.7225199366763 | 22.9305060782808 | 73.1788703715725 | 26.2420547063975 | 73.6429343984318 | 26.7308510821762 | 75.7601610933929 | 26.3926437512669 | 57.3699204455319 | 30.2818488510806 | 70.7896430125137 | 22.9131395280711 | 71.9854033909295 | 23.7197756130234 | 71.9854033909295 | 23.7197756130234 |
| 80 | 77.8404382425863 | 20.9644274128473 | 78.3248235583895 | 22.9787428648552 | 72.796230512063 | 26.2364901399759 | 73.2695171181439 | 26.6452331800071 | 75.4582891736394 | 26.308532260543 | 57.1947607575052 | 30.1541266711059 | 70.4419980079069 | 22.479084973584 | 71.6441894771477 | 23.4094382632327 | 71.6441894771477 | 23.4094382632327 |
| 81 | 77.421937836721 | 20.9554885504478 | 77.9494947770882 | 23.0044246330834 | 72.4537004591988 | 26.2023191545448 | 72.9571181602361 | 26.4972823064977 | 75.2032802026516 | 26.1473741848074 | 57.132792506673 | 29.9657800976569 | 70.206237988521 | 21.9255403593857 | 71.4223286796024 | 23.0066079942619 | 71.4223286796024 | 23.0066079942619 |
| 82 | 77.01597258298 | 20.932780926055 | 77.6006046095605 | 23.002665537885 | 72.1588163418498 | 26.1337666338089 | 72.7165404535802 | 26.2764833591859 | 74.9972120113539 | 25.9012485005893 | 57.2009403356404 | 29.7025140274842 | 70.1058388824515 | 21.2354744452768 | 71.3325770355482 | 22.4934830746106 | 71.3325770355482 | 22.4934830746106 |
| 83 | 76.6244363354204 | 20.894300942887 | 77.2820320321672 | 22.9682704379018 | 71.9200101812972 | 26.0236153122644 | 72.5581630108077 | 25.9729401444088 | 74.8415956882308 | 25.5656259026348 | 57.412487120273 | 29.3526435810228 | 70.1586928410517 | 20.3981551339948 | 71.3815721748599 | 21.8515015193158 | 71.3815721748599 | 21.8515015193158 |
| 84 | 76.2493235681339 | 20.838063715977 | 76.997038496919 | 22.8958760434249 | 71.7464993507503 | 25.8629059464495 | 72.4910485337379 | 25.5782412984249 | 74.7373577936217 | 25.1410388997364 | 57.7746323158707 | 28.9093916717676 | 70.3726681160499 | 19.4128471461237 | 71.5667728158533 | 21.0619867236621 | 71.5667728158533 | 21.0619867236621 |
| 85 | 75.8926363232839 | 20.7621991763701 | 76.747705274122 | 22.7801412262403 | 71.6480891635641 | 25.6405951917643 | 72.5217854529015 | 25.0865547983092 | 74.6848503133789 | 24.6351111087907 | 58.2854241863426 | 28.3737889045366 | 70.7399992747215 | 18.2933577746973 | 71.8727089448777 | 20.1070009128055 | 71.8727089448777 | 20.1070009128055 |
| 86 | 75.5562505409273 | 20.6650735114305 | 76.53421083152 | 22.6159940483848 | 71.6348692626623 | 25.3431676924916 | 72.6530229370616 | 24.4959830064485 | 74.683893815257 | 24.0649904833726 | 58.9299715636377 | 27.7582613083706 | 71.230339819501 | 17.0735394831667 | 72.2664513768708 | 18.9704388490648 | 72.2664513768708 | 18.9704388490648 |
| 87 | 75.2417337499639 | 20.5454393977642 | 76.3539234245466 | 22.3989436780108 | 71.7167824320672 | 24.9541987696739 | 72.8816538808655 | 23.8102126436911 | 74.7338596899458 | 23.4602343701197 | 59.6758326918907 | 27.090998095002 | 71.7822960079711 | 15.8138658840135 | 72.692202483687 | 17.6393969951297 | 72.692202483687 | 17.6393969951297 |
| 88 | 74.9501052891248 | 20.4026187414578 | 76.2002819590639 | 22.1254659929761 | 71.9030423116574 | 24.4538639690292 | 73.1965972210396 | 23.0404966196868 | 74.8337977886323 | 22.8661975422054 | 60.467470312772 | 26.4211985472808 | 72.2932469411604 | 14.6092056536878 | 73.0649027335487 | 16.1058561899931 | 73.0649027335487 | 16.1058561899931 |
| 89 | 74.6815293825374 | 20.2367219069746 | 76.0614359968247 | 21.7934723264206 | 72.2013742683131 | 23.8183916091085 | 73.5761271438625 | 22.2080072647102 | 74.9826162103942 | 22.3479777141452 | 61.2196534453805 | 25.8253042836692 | 72.607241802538 | 13.5979272027299 | 73.2627403084385 | 14.3687188550585 | 73.2627403084385 | 14.3687188550585 |
| 90 | 74.434930516089 | 20.0489076911284 | 75.9186135048442 | 21.4028714834483 | 72.6170513604711 | 23.0194543484207 | 73.9846928290241 | 21.3466032052748 | 75.179320445569 | 21.9949764961232 | 61.8096773056875 | 25.4143305149058 | 72.5007504869995 | 12.9724754279 | 73.1184434566314 | 12.4362448105864 | 73.1184434566314 | 12.4362448105864 |

## **Quality of Life**

For taking into account the quality of life with CKD, the model assigns a value for quality-adjusted life-year (QALY) depending on the GFR [14], using EuroQol measurement (EQ-5D) [15]. **Table S13** shows the values used for assigning QALY in the S-GASM.

| **Table S13** – QALY depending on GFR[14] | |
| --- | --- |
| GFR | QALY |
| >=90 | 0,94 |
| >=60 <90 | 0,918 |
| >=30 <60 | 0,883 |
| >=15 <30 | 0,839 |
| <15 | 0,798 |

## **Testing**

The target group for the testing can be selected from all the parameters used in the S-GASM. The model uses two-time urinary albumin-creatinine for testing, as recommended in guidelines, with the sensitivity being 87 percent and the specificity being 88 percent for one test [16]. In our model, the two-time control differs from one-time control only in sensitivity, specificity, and costs. Both take place on the same annual follow-up in the simulation. We can implement other testing options later if comparison should be a goal.

## **Therapy caused by testing and adherence**

For therapy, the model assigns everyone who tested positive or false-positive Angiotensin-converting-enzyme inhibitors (ACEI) because they are considered to be advantageous over Angiotensin-II-receptor blockers (ARB) [8, 17].

The therapy initiated after the testing subsequently affects the loss of GFR [7], the progression from micro to macroalbuminuria, and the risk of death [8]. **Table S6** shows the values used for these effects. Though, these effects only take place with consideration of randomly assigned adherence, with 91 percent of individuals being adherent [18]. If individuals have already been assigned ACEI or ARB through anti-hypertensive therapy, they will receive the same effects regardless of adherence. We can implement other therapy options later if comparison should be a goal.

| **Table S6** – Effects of Medication used in the S-GASM | |
| --- | --- |
| Reduced GFR loss due to ACEI or ARB[7] | 36% |
| The relative risk for the transition from micro- to macroalbuminuria due to ACEI or ARB[8] | 45% |
| The relative risk for death due to ACEI[8] when an individual has albuminuria | 79% |

## **Costs**

We simplified the costs of renal replacement therapy by only using the annual costs of dialysis therapy and associated costs (54,777 € in 2006) [19]. We estimated them to be 63,000 € in 2016 due to inflation.

We calculated the cost of the urinary albumin-creatinine-based testing procedure using the Physicians Fee Regulations 2015.[20] For a consultation (No. 1) and two-time measurement of albumin (No. 3735) and creatinine (No. 3585.H1) in a urine sample, this adds up to 36.18 € per testing for the first year. We chose the middle of three tariffs for each.

We estimated the costs of the ACEI prescribed after the testing by research in online pharmacies and set them to 15 € for 100 days in the first year.[21]

We decided to use high costs of testing and therapy to obtain conservative estimates in case of positive effects of testing.

In the simulation, we use a yearly discount rate of 3.5 percent for costs and benefits according to the NICE guidelines [22].

## **Death**

The model calculates the risk of death for an individual with the official German mortality table [23] and relative risks, according to GFR and Albuminuria [24]. **Table S14** shows the relative risks used in the S-GASM. Coincident albuminuria and ACEI therapy reduce this risk to 79%[8]. We lowered the baseline mortality rates so that the final mortality rate again matches the mortality table despite being influenced by other factors. We applied the same considerations we described in the diabetes section of this supplemental material to adjust the risks. **Table S15** shows the baseline risks of death the model uses. **Fig S9** shows the risk of death in a simulation of 1 million individuals compared to published data.

**Fig S9** – Risk of death (all causes) for females (left) and males (right) with published and measured data out of a simulation of 1 million individuals


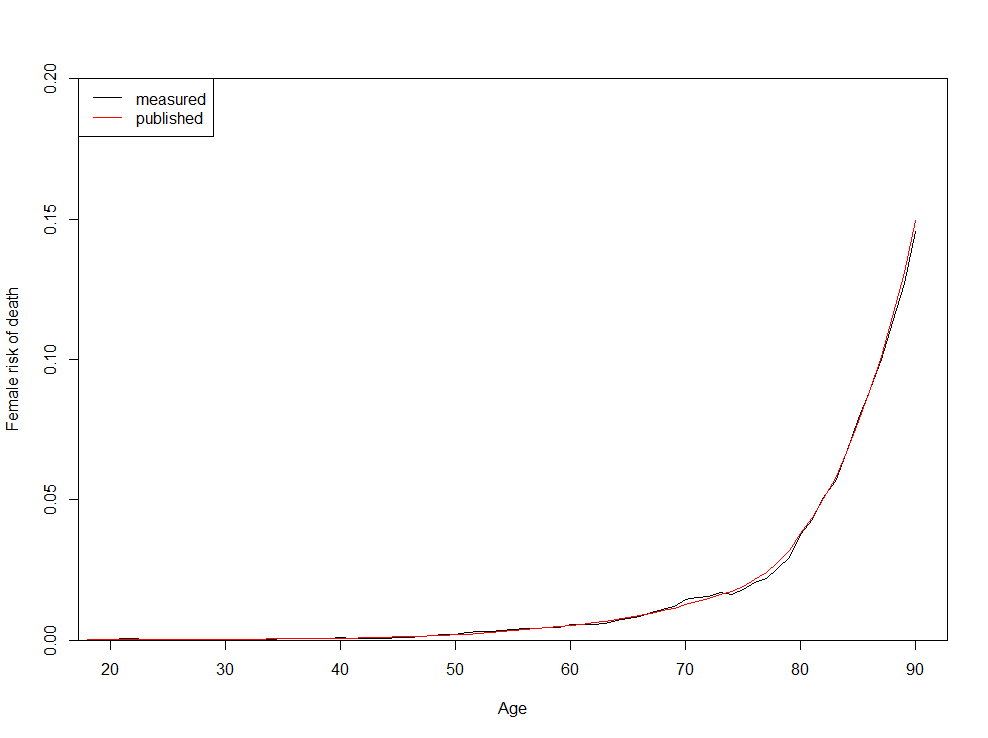

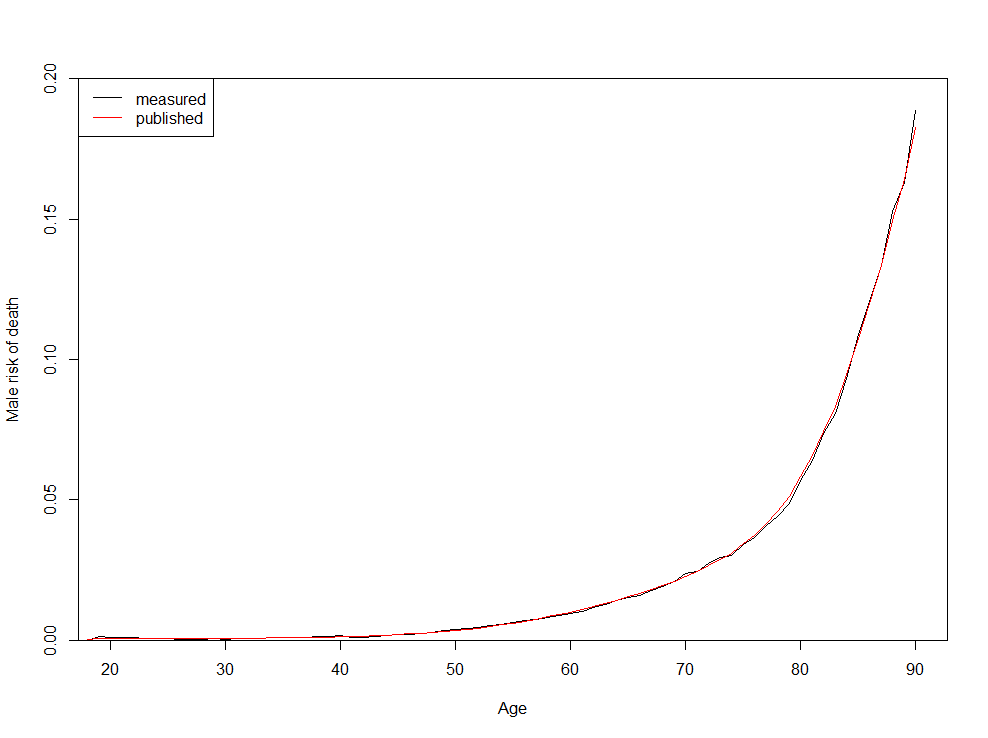


| **Table S14** – Relative risks for Death depending on GFR and Albuminuria [24] | | | |
| --- | --- | --- | --- |
| GFR | Albuminuria | | |
|  | none | micro | macro |
| >105 | 1,23 | 2,88 | 7,63 |
| 90-104 | 1 | 1,74 | 3,97 |
| 75-89 | 0,91 | 1,61 | 2,07 |
| 60-74 | 0,99 | 1,78 | 2,49 |
| 45-59 | 1,09 | 1,94 | 2,92 |
| 30-44 | 1,51 | 2,58 | 3,58 |
| 15-29 | 3,22 | 2,99 | 5,75 |
| <15 | 3,81 | 6,22 | 9,68 |

| **Table S15** – Risks for death used in S-GASM | | |
| --- | --- | --- |
| Age | Females | Males |
| 18 | 0.000132867835115628 | 0.000279161643229993 |
| 19 | 0.000125036195947992 | 0.000388340656092201 |
| 20 | 0.0001326586885918 | 0.000388999048830103 |
| 21 | 0.000124674666320106 | 0.000391789897366873 |
| 22 | 0.000123613392926887 | 0.00038409737071011 |
| 23 | 0.000142251895104124 | 0.00037237137863114 |
| 24 | 0.000129075676020916 | 0.000362090972247625 |
| 25 | 0.000149082872776688 | 0.000330867560489919 |
| 26 | 0.000151993466513137 | 0.000316093768235825 |
| 27 | 0.000156204471227249 | 0.000318161153021713 |
| 28 | 0.000158401715766508 | 0.000356215588615611 |
| 29 | 0.000160444012570508 | 0.00039192416258615 |
| 30 | 0.000227430431142663 | 0.000368683477987625 |
| 31 | 0.000215817029523165 | 0.000519764239374067 |
| 32 | 0.000228951092544256 | 0.000495930696958961 |
| 33 | 0.000221894222173216 | 0.000549664614150987 |
| 34 | 0.000314812664070667 | 0.000633667988238323 |
| 35 | 0.00028606014810029 | 0.000728164503279711 |
| 36 | 0.000325522008027822 | 0.000753047650590958 |
| 37 | 0.00033960266908992 | 0.000986574821255525 |
| 38 | 0.000619197841117459 | 0.00100536355558293 |
| 39 | 0.00056212200169867 | 0.00111251179641655 |
| 40 | 0.000861738795412173 | 0.00156085237147622 |
| 41 | 0.000351505243245022 | 0.000911109588328185 |
| 42 | 0.000406877773074931 | 0.000991224092860303 |
| 43 | 0.000501649486162552 | 0.00111835317152608 |
| 44 | 0.000611772391427616 | 0.00141763584922997 |
| 45 | 0.000790603159471117 | 0.0016823274710487 |
| 46 | 0.00095112107443296 | 0.00186616723987787 |
| 47 | 0.00119092233014704 | 0.00209303999435029 |
| 48 | 0.00156984000281828 | 0.00245578639739145 |
| 49 | 0.00185373705702434 | 0.00278302104196772 |
| 50 | 0.00188207379938853 | 0.00311608872261147 |
| 51 | 0.00223283536824093 | 0.00336058942602706 |
| 52 | 0.00254628084896616 | 0.00390784083528129 |
| 53 | 0.00273884785417953 | 0.00436091267849982 |
| 54 | 0.00301746582032362 | 0.00497731191887137 |
| 55 | 0.00321254192970241 | 0.00526926497048129 |
| 56 | 0.00333172426187583 | 0.00578496265063692 |
| 57 | 0.00350360229711208 | 0.00601511356074638 |
| 58 | 0.00380018982657493 | 0.00688857444100598 |
| 59 | 0.00373985438938082 | 0.00735110183132679 |
| 60 | 0.00447362732705625 | 0.00769626466306342 |
| 61 | 0.00452027569782856 | 0.00843247616077913 |
| 62 | 0.00461530170587059 | 0.00930401760334992 |
| 63 | 0.00501019896835845 | 0.010174007168297 |
| 64 | 0.00563402748428103 | 0.0111049010499234 |
| 65 | 0.00621535302052268 | 0.0120800684067565 |
| 66 | 0.0069936491498217 | 0.0124565601112857 |
| 67 | 0.00800700477415919 | 0.0138056365129826 |
| 68 | 0.00910624737083458 | 0.015159555307651 |
| 69 | 0.00922706242307257 | 0.0161133456196359 |
| 70 | 0.0113104808809482 | 0.0180554244575872 |
| 71 | 0.0117107284507706 | 0.0189881776621135 |
| 72 | 0.0123181014463164 | 0.0205786284546186 |
| 73 | 0.0131788151516207 | 0.0226006352038541 |
| 74 | 0.0128335230431175 | 0.0227284418863777 |
| 75 | 0.0139451987493126 | 0.025851899384473 |
| 76 | 0.015212187107909 | 0.0273593280555323 |
| 77 | 0.0165677002703607 | 0.0300038684904205 |
| 78 | 0.0188914372992256 | 0.0326435755668009 |
| 79 | 0.0219601844439234 | 0.0356893844550381 |
| 80 | 0.0273930156818294 | 0.0416237993601528 |
| 81 | 0.0311042634878721 | 0.0466500861673741 |
| 82 | 0.0360631033131038 | 0.0531015688014184 |
| 83 | 0.0400115579828011 | 0.0579249248911926 |
| 84 | 0.0472899099026803 | 0.0668175808880798 |
| 85 | 0.0558709899228372 | 0.077444656783425 |
| 86 | 0.062833064098344 | 0.0856492075666371 |
| 87 | 0.0701185958125871 | 0.0945466711045953 |
| 88 | 0.0793408279831212 | 0.107315465623532 |
| 89 | 0.0890072990688581 | 0.115979900926629 |
| 90 | 0.101781627840517 | 0.132601941891117 |

# **Sensitivity Analysis**

We performed a sensitivity analysis with 1 million individuals and a gradually 5 percent increase from minus 20 to plus 20 percent in BMI, SBP, diabetes prevalence and incidence, albuminuria incidence, and GFR loss. After that, we compared the incidence of RRT and the lifetime costs of RRT per individual.

**
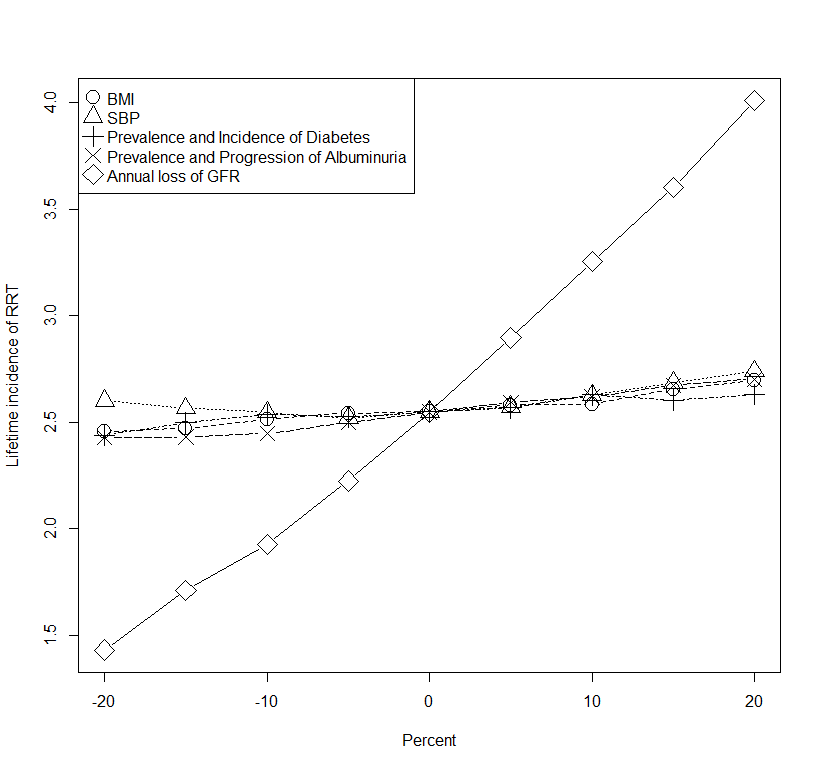
**
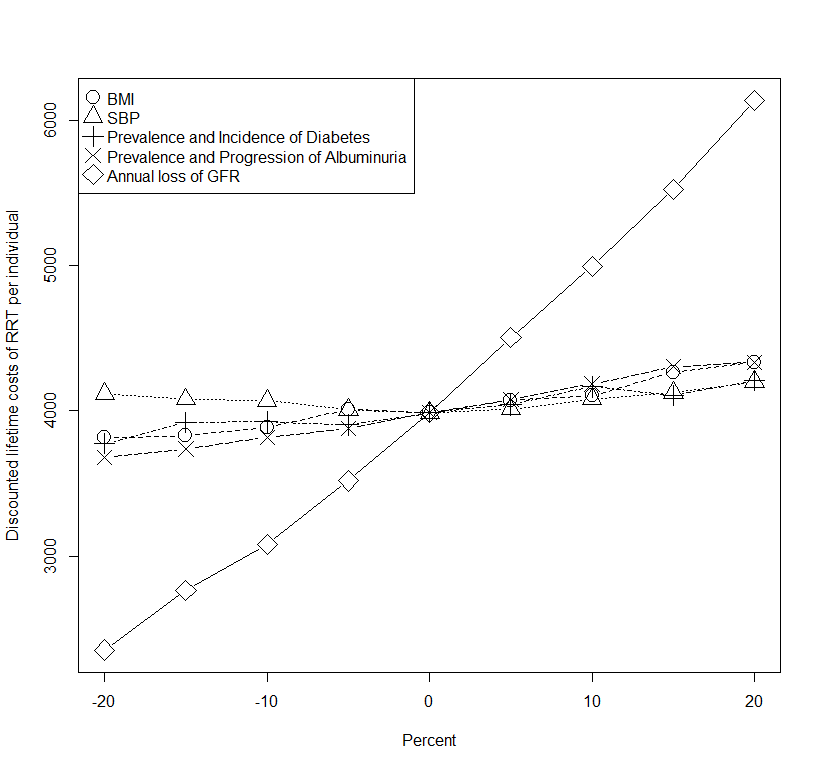

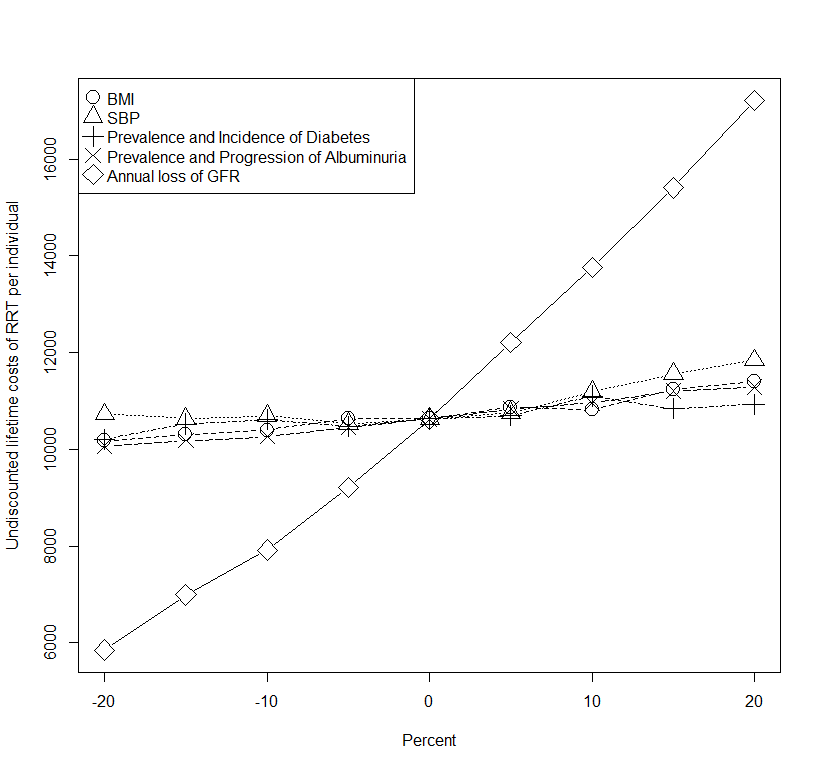
Figs S10, S11, and S12 show the results of the performed sensitivity analysis. While most of the parameters had only minor influence, there was a strong effect of the assumed loss of GFR on both the incidence of RRT and the lifetime costs per individual.

**Fig S10. Results of sensitivity analysis with 1 million individuals.** A gradually 5 percent relative increase from minus 20 to plus 20 percent for each parameter shown regarding lifetime incidence of RRT

**Fig S11 (left) and Fig S12 (right). Results of sensitivity analysis with 1 million individuals.** A gradually 5 percent relative increase from minus 20 to plus 20 percent for each parameter shown regarding lifetime costs of RRT per individual, undiscountend (left) and discounted (right)

We observe a slight increase in the incidence of RRT and their costs on reducing the SBP. We believe this to happen because hypertension is the possibility of getting ACEI which lowers the annual loss of GFR.

# **Validation Analysis**

In terms of validation, we compared the distribution of age, sex, BMI, SBP, diabetes, and the risk of death with the underlying data and presented the results in their corresponding section of this file.


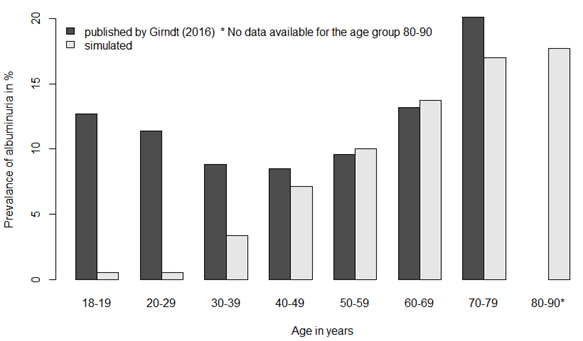
Fig S13 demonstrates the prevalence of albuminuria in a simulation of 10 million individuals compared to published data by Girndt et al. [25]. The simulated prevalence of albuminuria increases from 0.57% in the youngest age group to 17.77% for ages above 80.

***Fig S13.*** *- Prevalence of albuminuria in a simulation with 10 million individuals compared to published data [25].*

The model predicts a considerably lower prevalence of albuminuria at younger ages than published by Girndt et al. [25].

Our model utilizes data based on NHANES III, and comparing the simulated with these values, the difference is not that big. The values published by Girndt et al. differ significantly from this, and we are unsure which values to rely on. Especially since a probably comparable population from the Netherlands in the PREVEND study provides data that match the values from NHANES III, but also Zhou et al., a more recent study from the US, provides even higher values than Girndt et al.

| **Table S16** – Prevalence of albuminuria in younger Age groups | | | | |
| --- | --- | --- | --- | --- |
| **Study** | **Country** | **18-19** | **20-29** | **30-39** |
| Garg [26]  NHANES III 1988-1994 | USA |  | 5.7%  (n=3071) | 5.5%  (n=2928) |
| Gracchi [27]  PREVEND 1997-1998  (*N=40000*) | Groningen, NL |  | ≈ 5.8% | ≈ 5.7% |
| Girndt [25]  2008-2011 | Germany | 12.7%  (n=205) | 11.9%  (n=781) | 8.8%  (n=830) |
| Zhou [28]  2011-2018 | USA + Canada | 24.7%  (n =506) | | |

This increased prevalence of albuminuria in younger ages should not affect the testing costs. Solely the costs of subsequent therapy would be higher. If we assume that these cases of “young albuminuria” have no impact on the incidence of renal replacement therapy in the later course, from a strict economic cost-effectiveness point of view, the influence from the younger age groups on the overall results is relatively small because the costs of an annual test (36,18€) are very similar to the costs of therapy for one year (54,74€) and due to the cohort-setting, 11% of all simulated person-years fall within the ages under 40. However, changing the period between the tests from one or two years increases this influence.

In addition, the S-GASM is based on the concept of a steady progression of albuminuria and CKD. In reality, albuminuria is reversible, and evidence exists that ACEI increases regression rate [8]. This could improve even more the cost-effectiveness of testing.

Also, the prevalence of RRT could be lower if more treatments in younger ages were performed, and the cost-effectiveness of testing could be increased. Though the data published by the authors mentioned are not detailed enough to map these aspects in the S-GASM as no relative risks, according to risk factors, and no incidence and regression rates are provided.

With a higher prevalence of albuminuria in younger age groups, the rate of individuals tested positive would be much higher, which could have effects for the individuals, such as the risk of adverse clinical effects, costs of medication, and nonclinical costs for transportation, childcare, or lost wages for additional consultations. Also, the psychological effects of being labeled at risk for chronic disease are not taken into account.

We would like to encourage further research into the field of albuminuria in childhood and adolescence and its effects on the individual course of albuminuria and GFR in adulthood, as this has potentially a significant influence on our topic.

Until we are able to engage this topic with further insights, it is possible to limit the testing to individuals older than 40 to reach maximum conservativeness. We decided not to limit the testing for our exemplary results because we assess the S-GASM to be working as intended with the data it is based on, there is no final evaluation on the topic of albuminuria in younger individuals possible, and the changes in the results are to be estimated as minor.

Fig S14 shows the simulated incidence of RRT compared to published data by Icks et al. [12]. The measured incidence ranges from 5.4 per 100,000 individuals for the age group of 30-49 to 147 per 100,000 individuals for the age of 80 and above, while published numbers for these age groups are 5.3 per 100,000 and 169.8 per 100,000. In the no-testing condition, the lifetime prevalence of RRT has been 2.55%.


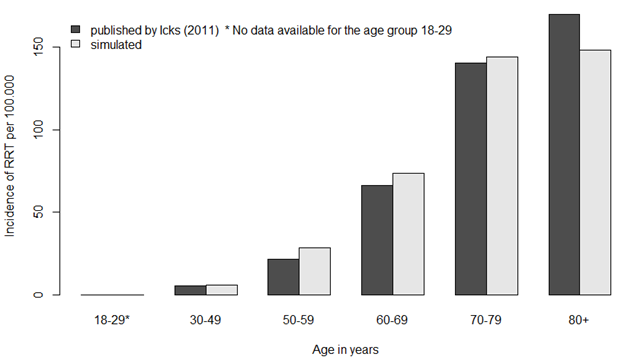


**Fig S14 -** Incidence of RRT in a simulation with 10 million individuals compared to published data [12].

The incidence of RRT in the simulation is close to published data and also reflects the age-related increase. We assume that the slight overestimation in the prior age groups is the reason for the higher difference in the oldest age group.

As we could not find up-to-date data for Germany, we could not compare the simulated lifetime prevalence of the dialysis treatment of 2.55%. Nevertheless, the simulated lifetime prevalence is close to data published for other European countries given as up to 2.05% [29]. We see the cause of this overestimation in the fact that, unlike in the model, in reality, the decision for dialysis treatment is not solely made on GFR. It is conceivable that patients do not get treatment due to other individual aspects. Along with the expectation of increasing numbers of dialysis patients [30], this could partly explain the overestimation.

Table S16 shows the times spent in CKD stages 3a to 5 compared to published data by Ku et al. [31].

| **Table S16 -** Times spent in CKD stages in a simulation of 10 million individuals compared to published data [31] | | |
| --- | --- | --- |
| Stage of CKD | Simulated in the S-GASM  Median time (25%– 75% quantile) in years | Published by Ku et al. [31]  Median time (25% – 75% quantile) in years |
| Stage 3a | 7  (4-14) | 7.9  (2.3 to >12) |
| Stage 3b | 5  (3-9) | 5.0  (1.8 to 11.5) |
| Stage 4 | 4  (2-6) | 4.2  (2.4 – 6.6) |
| Stage 5 | 2  (1-3) | 0.8  (0.3-1.6) |

The simulated times spent in the later stages of CKD are close to the times published by Ku et al. In our opinion, the differences are mainly based on the concept of the S-GASM, with annual follow-ups and where no other events are possible in-between.

# **Citations**

1. Statistisches Bundesamt. *Bevölkerung und Erwerbstätigkeit 2016 - Bevölkerungsfortschreibung auf Grundlage des Zensus 2011*. 2019; Available from: <https://www.destatis.de/DE/Themen/Gesellschaft-Umwelt/Bevoelkerung/Bevoelkerungsstand/Publikationen/Downloads-Bevoelkerungsstand/bevoelkerungsfortschreibung-2010130167004.pdf?__blob=publicationFile&v=4>.

2. Hemmelmann, C., et al., *[Percentiles of body mass index of 18-80-year-old German adults based on data from the Second National Nutrition Survey].* Dtsch Med Wochenschr, 2010. **135**(17): p. 848-52.

3. Cole, T.J. and P.J. Green, *Smoothing Reference Centile Curves - the Lms Method and Penalized Likelihood.* Statistics in Medicine, 1992. **11**(10): p. 1305-1319.

4. Balijepalli, C., et al., *Percentile distribution of blood pressure readings in 35683 men and women aged 18 to 99 years.* J Hum Hypertens, 2014. **28**(3): p. 193-200.

5. Adler, C., et al., *Change in the association of body mass index and systolic blood pressure in Germany - national cross-sectional surveys 1998 and 2008-2011.* BMC Public Health, 2015. **15**: p. 705.

6. Sarganas, G., et al., *Trends in Antihypertensive Medication Use and Blood Pressure Control Among Adults With Hypertension in Germany.* Am J Hypertens, 2016. **29**(1): p. 104-13.

7. Agodoa, L.Y., et al., *Effect of ramipril vs amlodipine on renal outcomes in hypertensive nephrosclerosis: a randomized controlled trial.* JAMA, 2001. **285**(21): p. 2719-28.

8. Strippoli, G.F., et al., *Effects of angiotensin converting enzyme inhibitors and angiotensin II receptor antagonists on mortality and renal outcomes in diabetic nephropathy: systematic review.* BMJ, 2004. **329**(7470): p. 828.

9. Wilke, T., et al., *[Incidence and prevalence of type 2 diabetes mellitus in Germany: an analysis based on 5.43 million patients].* Dtsch Med Wochenschr, 2013. **138**(3): p. 69-75.

10. Rathmann, W. and C. Meisinger, *How prevalent is type 2 diabetes in Germany? Results from the MONICA/KORA studies.* Diabetologe, 2010. **6**(3): p. 170-+.

11. Yarnoff, B.O., et al., *Modeling the impact of obesity on the lifetime risk of chronic kidney disease in the United States using updated estimates of GFR progression from the CRIC study.* PLoS One, 2018. **13**(10): p. e0205530.

12. Icks, A., et al., *Incidence of renal replacement therapy (RRT) in the diabetic compared with the non-diabetic population in a German region, 2002-08.* Nephrol Dial Transplant, 2011. **26**(1): p. 264-9.

13. Chen, T., V.W. Lee, and D.C. Harris, *When to initiate dialysis for end-stage kidney disease: evidence and challenges.* Med J Aust, 2018. **209**(6): p. 275-279.

14. Tajima, R., et al., *Measurement of health-related quality of life in patients with chronic kidney disease in Japan with EuroQol (EQ-5D).* Clin Exp Nephrol, 2010. **14**(4): p. 340-8.

15. EuroQol Group, *EuroQol--a new facility for the measurement of health-related quality of life.* Health Policy, 1990. **16**(3): p. 199-208.

16. Wu, H.Y., et al., *Diagnostic performance of random urine samples using albumin concentration vs ratio of albumin to creatinine for microalbuminuria screening in patients with diabetes mellitus: a systematic review and meta-analysis.* JAMA Intern Med, 2014. **174**(7): p. 1108-15.

17. Xie, X., et al., *Renin-Angiotensin System Inhibitors and Kidney and Cardiovascular Outcomes in Patients With CKD: A Bayesian Network Meta-analysis of Randomized Clinical Trials.* Am J Kidney Dis, 2016. **67**(5): p. 728-41.

18. Schweitzer, R.D., K. Head, and J.W. Dwyer, *Psychological factors and treatment adherence behavior in patients with chronic heart failure.* J Cardiovasc Nurs, 2007. **22**(1): p. 76-83.

19. Icks, A., et al., *Costs of dialysis--a regional population-based analysis.* Nephrol Dial Transplant, 2010. **25**(5): p. 1647-52.

20. Verband der Privatärztlichen Verrechnungsstellen e.V. *Gebührenordnung für Ärzte*. 2015; Available from: <https://www.pvs-se.de/fileadmin/user_upload/redakteure/pvs-se/dokumente/Gebuehrenordnungen/GOAE_Auflage_2015_WEB.pdf>.

21. Centalus Media GmbH. *Preisvergleich Ramipril 100 St.* 08.09.2019]; Available from: <https://www.medipreis.de/suchen?q=ramipril&p%5Bsize%5D=100+St%C3%BCck>.

22. Excellence, N.I.f.H.a.C., *CHTE Methods Review: Discounting*. 2020: <https://www.nice.org.uk/Media/Default/About/what-we-do/our-programmes/nice-guidance/chte-methods-consultation/Discounting-task-and-finish-group-report.docx#:~:text=NICE%20has%20recommended%20a%203.5,for%20health%20effects%20was%20recommended>.

23. Statistisches Bundesamt. *Sterbetafel 2015/2017 (Periodensterbetafel): Deutschland, Jahre, Geschlecht, Vollendetes Alter*. 2019 [cited 2019 03.29.]; Available from: <https://www-genesis.destatis.de/genesis/online/data?operation=previous&levelindex=2&step=2&titel=Ergebnis&levelid=1574180339459&acceptscookies=false>.

24. Wen, C.P., et al., *Relative risks of chronic kidney disease for mortality and end-stage renal disease across races are similar.* Kidney Int, 2014. **86**(4): p. 819-27.

25. Girndt, M., et al., *The Prevalence of Renal Failure. Results from the German Health Interview and Examination Survey for Adults, 2008-2011 (DEGS1).* Dtsch Arztebl Int, 2016. **113**(6): p. 85-91.

26. Garg, A.X., et al., *Albuminuria and renal insufficiency prevalence guides population screening: results from the NHANES III.* Kidney Int, 2002. **61**(6): p. 2165-75.

27. Gracchi, V., et al., *Prevalence and distribution of (micro)albuminuria in toddlers.* Nephrol Dial Transplant, 2016. **31**(10): p. 1686-92.

28. Zhuo, M., et al., *High Prevalence and Low Awareness of Albuminuria in the Community Setting in the KDSAP.* Kidney Int Rep, 2020. **5**(4): p. 475-484.

29. van den Brand, J., et al., *Lifetime risk of renal replacement therapy in Europe: a population-based study using data from the ERA-EDTA Registry.* Nephrol Dial Transplant, 2017. **32**(2): p. 348-355.

30. Medical Netcare GmbH. *Jahresbericht 2016 zur Qualität in der Dialyse*. 2017; Available from: <https://www.g-ba.de/downloads/17-98-4424/2017-07-20_QSD-RL_MNC-Jahresbericht-2016_Bericht.pdf>.

31. Ku, E., K.L. Johansen, and C.E. McCulloch, *Time-Centered Approach to Understanding Risk Factors for the Progression of CKD.* Clin J Am Soc Nephrol, 2018. **13**(5): p. 693-701.
