# Supplementary material for: Development of the simulation-based German albuminuria screening model (S-GASM) for estimating the cost-effectiveness of albuminuria screening in Germany
Source: PLoS One. 2022 Jan 5;17(1):e0262227. doi: 10.1371/journal.pone.0262227 (PMC8730388; doi:10.1371/journal.pone.0262227)
Supplement: Attachment — Submitted filename: response_to_reviewers.docx [file pone.0262227.s002.docx]

*1. The manuscript would have been stronger if it adhered to reporting guidelines for economic evaluations, such as the CHEERS statement.*

We restructured our manuscript according to the CHEERS criteria.

*2. The manuscript seems to confuse case-finding (testing for albuminuria in a population with a risk factor such as diabetes) with population-based screening (testing for albuminuria in the general population)*

We implemented the linguistic differentiation between “testing” and “screening”. “Screening” is only used for testing in the general population.

*3. Abstract: the last sentence about possible applications of the GASM model are not supported by the results presented, and should be deleted.*

We hope and firmly believe that the changes made to the manuscript justify keeping the statement.

*4. Lines 68-69: the authors seem to suggest that the decision to test/screen for albuminuria is solely driven by its effectiveness or cost-effectiveness. This is overly simplistic, especially for screening, which by definition is done in people who consider themselves healthy. No mention is made of labeling effects, harms due to false positives, patient preferences etc.*

We added a section on possible labeling effects and harms to false positives to the discussion section of the manuscript and added the rate of individuals tested false-positive for every scenario of testing.

*5. Lines 72-73: the authors critique prior work by stating that it mostly focused on testing in groups at risk….but then focus their analyses on people with diabetes.*

We wrote the first version of our manuscript focusing on the model development and less on concrete results. That is why the first version only contained an exemplary analysis. We added two more examples to show possibilities and compare results. We still believe a complete analysis of all possible scenarios using the model should be part of further work, as this involves variations in the risk factors to be tested, the age of entry for testing, the interval of tests, and methods of testing. A corresponding matrix with four dimensions would be too extensive to be presented, evaluated, and discussed as part of this work.

*6. The authors do not explain why it is important to repeat this analysis for a German population. What characteristics of the population or health system are unique such that results from other countries cannot be used?*

We are still convinced that the approach of our model has not yet been published in this form. An analysis of a large proportion of the total population with multiple risk factors, specific test methods, specific therapeutic options, and the possibility of comparing the results justifies, in our opinion, a renewed study of this topic.

We updated our introduction with current German guidelines considering testing for albuminuria. As these guidelines contradict each other in part, it seems necessary to devote renewed attention to this topic.

*7. The comparator for testing (which the authors call screening) is not explicitly stated. It seems to be the absence of testing. But of course, many people with diabetes are tested for albuminuria in the absence of a formal recommendation. The proportion who undergo ad hoc testing is not provided and seems to be 0. This will greatly exaggerate the estimated benefit of testing. Similarly, repeating the tests among those who were already tested will waste resources, and not capturing this also inflates the benefit of testing. Perhaps one or both of these were addressed, but because the comparator is not stated it is impossible to be sure. The manuscript would be stronger if it explicitly stated the comparator and addressed these issues.*

With the restructuring according to the CHEERS criteria, we have added the comparator section.

We added a section on the assumption we made in creating the model regarding testing for albuminuria in the base-case population without additional testing through our intervention. “We also assume the subgroup of individuals with diabetes already receiving ACEI or ARB to contain the individuals tested for albuminuria without additional testing through our intervention.” This subgroup will not be tested in our interventions because it already has the potential therapy.

*8. Table 1 should show not only the source of the model inputs, but the inputs themselves so that readers can judge whether they are reasonable.*

For reasons of space, we cannot display all values directly. The model uses large table data such as distributions, percentiles, and the mortality table. For us, it seems impossible to transform page-filling tables into a single field of a table.

We present all input values in the S1 File.

*9. Line 125: since the model was not well calibrated, an arbitrary adjustment was made so that the outputs better fit the data. This is not a well-accepted practice and calls the validity of the results into question. Similar “adjustments” are made in lines 136-137 and again on 164-165.*

While calibrating the model, we had to adjust multiple data. We explain the adjustments and show figures of the results in the S1 File.

For us, it seems reasonable to adjust input data when combining multiple data for one parameter when they are published separately. For example, upon combining an incidence for diabetes published with relative risks published elsewhere, we adjusted the risks that both values are represented in the end. This means the overall incidence matches its source while the relative risks for subgroups also apply. We provide a mathematical approach to these adjustments in the S1 File.

We also provide a figure for adjusting the annual loss of GFR in our S1 File comparing different levels to published data for the incidence of renal replacement therapy. We are aware that this approach can not be considered optimal. However, the lack of published data for the annual loss of GFR in Germany required taking data from the US. Nevertheless, still, we believe calibrating the model and data while considering published data is not arbitrary.

We also provide a validity analysis in our S1 File.

*10. Lines 157-158: a second issue with the comparator is that the paper does not report the proportion of people with diabetes (with or without albuminuria) who use ACEI/ARB in the absence of systematic testing. If most people with diabetes already use ACE/ARB or will do so in future, then there is little benefit of systematic testing. Again, this likely inflates the modeled benefit of testing and highlights the importance of systematic reporting.*

We assume individuals with diabetes to possibly receive ACEI/ARB only when they are also hypertensive. Individuals with hypertension receive their therapy according to a distribution where receiving ACEI/ARB is one of the multiple options. If an individual already receives ACEI/ARB, it is not tested in any scenario.

We present the values for the distribution of the therapy of hypertension in the S1 File. For reasons of space, we cannot display all values directly.

*11. I did not see information on discounting or opportunity costs.*

We added discounting of costs of benefits according to nice guidelines. Because there is a controversial discussion about discounting, we decided to present discounted and undiscounted values.

*12. The perspective taken in the economic analysis is not stated. It seems to be the perspective of a government payor.*

We added a statement about the healthcare system perspective.

*13. Lines 281-283: the authors close by stating that their model is suitable for evaluating (?comparing) different strategies for albuminuria testing and to develop recommendations. This statement is not supported by the data they present, and (given comment #9 above), there is some evidence that it may not be suitable for this purpose.*

We hope that the adjustments we made to the manuscript now allow this statement to be part of the manuscript still.

As on writing this response, we are unsure whether the criticism about the word “evaluating” is professionally justified or personal preference as for us evaluate means:

evaluate: to judge or calculate the quality, importance, amount, or value of something [dictionary.cambridge.org]

*Reviewer #2: In this manuscript, Kairys et al presented an interesting simulation-based analysis on the effects of albuminuria screening, subsequent treatment, and impact on adverse health outcome in people with diabetes. The built model consisted of an individual-based simulation of disease progression adjusting for several demographic, clinical and lab (eGFR) factors.

Comments
1. The study design: It stated a cost-effectiveness analysis but remains unclear if this was simulated from a societal or health system perspective.*

We added a statement about the healthcare system perspective.

*2. Model framework: Was this based on the state-transition Markov analytic model or other approaches were leveraged? This unclear in the manuscript. The statistics, and other analyses are not presented in sufficient details.*

We added a statement about the model being based on a Markov decision process. The model was implemented as an R-script, and we decided to present the code for detailed analysis.

*3. What published data informed the allocation of transition probabilities upon screening to the development of outcomes? For example, how did you model simulate the clinical path of patients from mild to moderate albuminuria to incident kidney failure (ESRD).*

The model is based on the assignment of an initial GFR and the annual loss of GFR. Both are dependent on multiple risk factors (age, sex, BMI, SPB, diabetes, albuminuria, therapy with ACEI/ARB), which also influence each other.

The model uses data for prevalence, incidence, and the progression of albuminuria based on data from Yarnoff et al. We derived values for the prevalence of albuminuria (and the initial GFR) for older age groups because the simulation of Yarnoff et al. only started with a cohort of individuals aged 30.

We reworked the methods section to clarify these aspects. *4. Lack of novelty. The screening (risk identification) for people with type 2 diabetes is already a widely adopted practice clinical practice and recommended by clinical practice guidelines (eg. KDIGO). It is difficult to see an incremental value of the present analysis on practice and/or policy implications.*

*5. Lack of structure for the manuscript: Please follow a standard framework in detailing the methods, presentation of results and discussion (e.g., Siegel et al. Recommendations for reporting cost-effectiveness analyses. Panel on Cost-Effectiveness in Health and Medicine; JAMA 1996 Oct 23-30;276(16):1339-41).*

We restructured our manuscript according to the CHEERS criteria.
