# Supplementary material for: Development of the simulation-based German albuminuria screening model (S-GASM) for estimating the cost-effectiveness of albuminuria screening in Germany
Source: PLoS One. 2022 Jan 5;17(1):e0262227. doi: 10.1371/journal.pone.0262227 (PMC8730388; doi:10.1371/journal.pone.0262227)
Supplement: Attachment — Submitted filename: Response_to_Reviewers.docx [file pone.0262227.s003.docx]

*Reviewer #1: This cost-effective analysis of the estimated effects of albuminuria screening seems to be rigorously scrutinized by the authors. The responses to the previous reviewers seem to be addressed appropriately. Although there are certainly several limitations to this estimation, those limitations were addressed in the literature.
I have just a few more questions.*

*1) I am concerned about the large gap in the presence of albuminuria and assumption in the analysis, as shown in the validation analysis, in the younger population. (To begin with, could such prevalence of albuminuria be really true in the general young population?) And as the authors mentioned, although the testing costs may not be affected, several burdens such as psychological and cost will greatly affect this population. I feel it is important to be aware of any evidence on the prevalence of albuminuria in the young general population and its pathological significance. The relevance of this estimation analysis would differ according to the pathological and treatment related significance (with ACEI/ARBs) in this population. How do the authors relate to this? Would the evidence really suggest a conservative assumption for this model (eg if treating young people with ACEI/ARBs have no influence on future outcomes) ? Maybe to be more conservative, it could be suggested that this model be better use for those above 40 years old?*

Our model utilizes data based on NHANES III, and if comparing the simulated with the published values, the difference is not big. The values published by Girndt et al. differ significantly from this, and we are unsure which values to rely on. Especially since a probably comparable population from the Netherlands in the PREVEND study provides data that match the values from NHANES III, but also Zhou et al., a more recent study from the US, provides even higher values than Girndt et al.

|  |  | **18-19** | **20-29** | **30-39** |
| --- | --- | --- | --- | --- |
| Garg [1]  NHANES III 1988-1994 | USA |  | 5.7% (n=3071) | 5.5% (n=2928) |
| Gracchi [2]  PREVEND 1997-1998 | Groningen, NL |  | ≈ 5.8% | ≈ 5.7% (*N=40000*) |
| Girndt [3]  2008-2011 | Germany | 12.7% (n=205) | 11.9% (n=781) | 8.8% (n=830) |
| Zhou [4]  2011-2018 | USA + Canada | 24.7%  (n =506) | | |

We are not really able to solve this problem but decided to emphasize the importance of new research in this direction, as there is a considerable influence on our topic.

If we assume that these cases of “young albuminuria” have no impact on the incidence of renal replacement therapy in the later course, from a strict economic cost-effectiveness point of view, the influence from the younger age groups on the overall results is relatively small because the costs of an annual test (36,18€) are very similar to the costs of therapy for one year (54,74€) and due to the cohort-setting, 11% of all simulated person-years fall within the ages under 40. However, changing the period between the tests from one or two years increases this influence.

Until we are able to engage this topic with further insights, it is possible to limit the testing to individuals older than 40 to reach maximum conservativeness. We decided not to limit the testing for our exemplary results because we assess the S-GASM to be working as intended with the data it is based on, there is no final evaluation on the topic of albuminuria in younger individuals possible, and the changes in the results are to be estimated as minor.

We decided to add a section about this problem to the manuscript and also added this discussion to the supplementary material.

*2) Line 163-166 the authors describe the factors integrated in the model but should not BMI be in there to?*

This section (Line 159-163 in the revised manuscript) was meant to describe the parameters directly influencing the prevalence, incidence, and progression of albuminuria, the initial GFR, and annual GFR loss. BMI is influencing indirectly through higher risks for hypertension and diabetes but not directly. We added the word „directly“ to make this clearer.

Line 117-121 of the revised manuscript describe all factors integrated into the model.

1.	Garg, A.X., et al., *Albuminuria and renal insufficiency prevalence guides population screening: results from the NHANES III.* Kidney Int, 2002. **61**(6): p. 2165-75.

2.	Gracchi, V., et al., *Prevalence and distribution of (micro)albuminuria in toddlers.* Nephrol Dial Transplant, 2016. **31**(10): p. 1686-92.

3.	Girndt, M., et al., *The Prevalence of Renal Failure. Results from the German Health Interview and Examination Survey for Adults, 2008-2011 (DEGS1).* Dtsch Arztebl Int, 2016. **113**(6): p. 85-91.

4.	Zhuo, M., et al., *High Prevalence and Low Awareness of Albuminuria in the Community Setting in the KDSAP.* Kidney Int Rep, 2020. **5**(4): p. 475-484.
